# Mental health outcomes associated with military sexual trauma in serving and ex-servicewomen: A systematic review

**DOI:** 10.1017/S003329172510175X

**Published:** 2025-09-29

**Authors:** Tamara Obradovic, Sarah Rabin, Dominic Murphy, Nicola T. Fear, Marie-Louise Sharp

**Affiliations:** 1 King’s Centre for Military Health Research, Department of Psychological Medicine, King’s College London, London, UK; 2 Research Department, Combat Stress, Leatherhead, UK; 3 Academic Department of Military Mental Health, King’s College London, London, UK; 4 School of Psychology, College of Life and Environmental Sciences, The University of Birmingham, Birmingham, UK

**Keywords:** Military sexual trauma, Sexual assault, Posttraumatic stress disorder, Depression, Social support, Systematic review, Women

## Abstract

Military sexual trauma (MST) (sexual harassment or sexual assault experienced during military service) is associated with adverse mental health outcomes. This systematic review assessed international, published, peer-reviewed academic literature and aimed to (1) identify the mental health outcomes of MST for serving and ex-servicewomen, (2) understand whether sexual harassment and sexual assault impact mental health differently, and (3) identify individual differences that may influence mental health outcomes. Included sources were peer reviewed, primary research, which investigated MST as a predictor of mental health outcome(s) in women. Database searches (June 2023, May 2024, and March 2025) yielded 63 studies, most of which (*n* = 58) were conducted in the United States and used quantitative methods (*n* = 60). A narrative synthesis approach facilitated data synthesis. Quantitative studies identified associations between MST and adverse mental health outcomes, with qualitative studies providing further context to these associations. Military sexual assault appeared to have a stronger relationship with adverse mental health than other MST experiences. Posttraumatic stress disorder and depression symptoms were associated with further outcomes, such as suicidality, disordered eating, and substance use. Some additional trauma exposures exacerbated the impacts of MST on mental health, whilst social support mitigated negative mental health outcomes. This review identifies significant mental health impacts of MST and highlights the importance of formal and informal support for serving and ex-servicewomen with MST experiences.

## Introduction

Military sexual trauma (MST), defined as sexual harassment or assault during military service (Galovski et al., 2022), predicts poor mental health (Surís & Lind, 2008). Whilst sexual trauma in non-military contexts is similarly detrimental to mental health (Dworkin, Menon, Bystrynski, & Allen, 2017), unique features of sexual violence during military service warrant consideration. Survivors may live, work, and socialize alongside perpetrators (Herriott, Campbell, Godier-McBard, Wood, & Murphy, 2024), and support pathways are often institutionally linked.

Such circumstances may contribute to Institutional Betrayal, where an institution an individual feels dependent on betrays their trust (Smith & Freyd, 2014). Institutional Betrayal may exacerbate the mental health impacts of MST (Smith & Freyd, 2013) through mechanisms like betrayal-based moral injury, arising from violations of trust by institutions or their members (Frankfurt et al., 2018; Lopes, McKinnon, & Tam-Seto, 2023). Other associated experiences include victim blaming (Lopes et al., 2023) and disengagement with military-associated healthcare services due to institutional distrust (Holliday & Monteith, 2019; Kelly, 2021).

Though MST is experienced by men and women, a meta-analysis of MST prevalence rates found 38.4% of women and 3.9% of men reported MST (Wilson, 2018). Women with MST experiences may experience higher risk of posttraumatic stress disorder (PTSD) and depression compared to men with MST experiences (Tannahill et al., 2020). The disproportionate exposure rates, coupled with distinct psychological sequalae, motivates elucidating women’s distinct needs.

The formal recognition of the term MST by the United States (US) Department of Veterans Affairs (VA) in 1992 prompted an influx of research, highlighting outcomes, including PTSD, harmful alcohol use, depression, and eating disorders (Allard, N, Gregory, Klest, & Platt, 2011; Surís & Lind, 2008). To our knowledge, there have not been any recent reviews of this literature.

This systematic review examines international research investigating mental health outcomes associated with experiencing MST in serving and ex-servicewomen to answer the following research questions:

1. What are the mental health outcomes of MST in serving and ex-servicewomen?
2. Do different MST experiences (e.g. experiencing military sexual harassment [MSH] or military sexual assault [MSA]) impact mental health differently?
3. Do individual differences (e.g. sociodemographic characteristics, military characteristics, and other trauma experiences) influence mental health outcomes following MST?

## Methods

### Study design

This systematic review followed the Preferred Reporting Items for Systematic Reviews and Meta-Analyses (PRISMA) guidelines (McGowan et al., 2016). An a priori registration of the review was submitted to PROSPERO (CRD42023429284).

### Searches

The search strategy was developed with guidance from the university librarian and the Peer Review of Electronic Search Strategies (PRESS) 2015 Checklist (McGowan et al., 2016). Search terms were related to (1) women/females (e.g. ‘Women’, ‘Female’, ‘Servicewoman’), (2) military (e.g. ‘Military Personnel’, ‘Veteran’, ‘Soldier’) (3) sexual trauma (e.g. ‘Sexual Assault’, ‘Sexual Violence’, ‘MST’), and (4) mental health (e.g. ‘Wellbeing’, ‘Distress’, ‘Mental Disorder’). An example of the search strategy, with a complete list of search terms, is available in the Supplementary Materials (S1).

Databases (CINAHL, EMBASE, MEDLINE, PILOTS, PsycINFO, Scopus, and Web of Science) were searched three times (June 2023 [Search 1], May 2024 [Search 2], and March 2025 [Search 3]), using free-text, subject heading searching using the explode function, plus reference, and citation searching. Searches were limited to studies published in English, with no restrictions on publication period or country.

### Eligibility

Included studies were required to:

- Be original, published, peer-reviewed, primary research, written in English and freely accessible to the review team.
- Include serving or ex-servicewomen in the sample.
- Present results for women separately.
- Asses mental health as a primary outcome of MST and include a comparison group who reported not experiencing MST (quantitative studies) or explore mental health in the context of MST (qualitative studies).

Case studies, gray literature, study registrations, protocols, and studies not specifically investigating MST were excluded.

### Study selection

Search 1 (June 2023) yielded 7928 papers, 5041 of which were duplicates and removed. Four studies were identified in a follow-up database search (Search 2; May 2024) and three through reference/citation searching. No additional papers were identified in Search 3 (March 2025). After screening 2891 titles/abstracts, 382 papers were assessed for eligibility.

**Figure 1.**
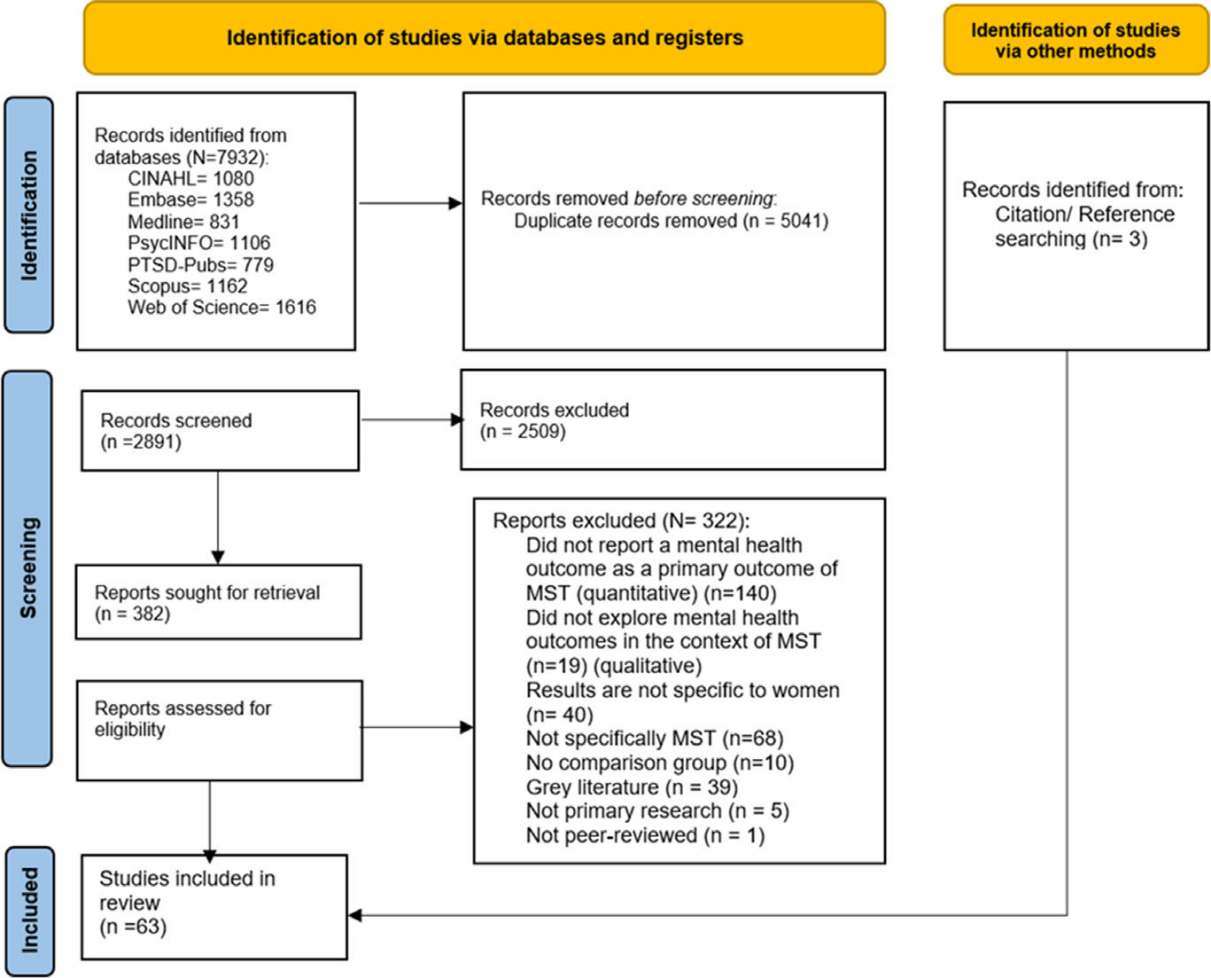
PRISMA flowchart.

The PRISMA (2020) flowchart (Figure 1) displays the selection process.

### Study quality

The National Heart, Lung and Blood Institute (NHLBI) Quality Assessment Tool for Observational Cohort and Cross-Sectional Studies (NHLBI, 2014) was used for the quality assessment of quantitative studies and the Critical Appraisal Skills Programme (CASP) Qualitative Studies Checklist (CASP, 2018) was used for qualitative studies. One author (TO) rated all studies. A random sample (10%) of studies were assessed by the second reviewer (SR) for validation. Discrepancies were resolved collaboratively. ‘Poor’-quality studies were included, but quality was considered when synthesizing findings.

### Data extraction

The following were extracted from all studies:

- Source.
- Study design.
- Aims.
- Participant eligibility criteria.
- Sample size.
- Participant demographic information.

#### Quantitative studies

The following were extracted from quantitative studies:

- Exposure to MST
  (definitions, measures used, and reported psychometric properties).
- Mental health outcome(s).
- Data analysis method.
- Participant response rate.
- Reported statistics.

#### Qualitative studies

For a meta-aggregative approach to qualitative synthesis (Lockwood, Munn, & Porritt, 2015), data extraction followed the Joanna Briggs Institute (JBI) Qualitative Data Extraction Tool (JBI, 2014) guidance. The following were extracted from qualitative studies:

- Population.
- Phenomena of interest.
- Context.
- Methodological framework.
- Methods.
- Findings and supporting evidence.

Extracted findings (themes, sub-themes, and authors’ analyses) were assigned one of three plausibility ratings based on supporting evidence from participant quotations: (1) unequivocal (fully supported by evidence), (2) equivocal (contestable considering the evidence presented), and (3) unsupported (not supported by evidence). Unsupported findings were excluded from data synthesis, but unequivocal and equivocal findings were considered equally. Well-supported findings, whether deemed fully supported or open to interpretation, were compared across studies, grouped into categories based on similarity, and refined into broader synthesized findings.

### Data synthesis

Heterogeneity in the MST-mental health literature (Surís & Lind, 2008) motivated a narrative synthesis approach (Popay et al., 2006). Quantitative and qualitative findings were considered collectively, with qualitative findings providing depth to quantitative results. The potential role of bias was considered when interpreting findings.

### Terminology

Included studies used the terms ‘female’ and ‘woman’ interchangeably. Though we acknowledge these distinct terms relate to biological sex and gender identity, respectively (Heidari, Babor, De Castro, Tort, & Curno, 2016), we mirror each study’s language when presenting study information to accurately reflect participant inclusion criteria. To promote clarity, the terms serving woman or ex-servicewoman are used to encapsulate both ‘female’ and ‘woman’ when presenting synthesized findings.

Given variation in how sexual harassment and sexual assault are defined in both the MST and wider sexual violence literature (Dworkin et al., 2017), we adopt broad definitions to maximize study inclusion. Sexual harassment is defined as unwanted behavior of a sexual nature, intended to upset, humiliate, offend, or scare another person (Equality and Human Rights Commission, 2010). Sexual assault is defined as non-consensual sexual touching (Sexual Offences Act, 2003).

Because terminology related to MST varied across studies, tables mirror terminology used within studies. For clarity in the synthesis, ‘MST’ is used to describe any experience capturing sexual harassment and/or assault during military service, ‘MSA’ to describe sexual assault during miliary service, and ‘MSH’ to describe sexual harassment during military service.

## Results

### Study characteristics

Sixty-three studies met the inclusion criteria (Table 1). Most used quantitative methods (*n* = 60) with cross-sectional study designs (*n* = 54). The remainder employed longitudinal study designs (*n* = 5), a nested case–control analysis (*n* = 1) and qualitative methods (*n* = 3). Most studies were US-based (*n* = 58). The remaining were conducted in the United Kingdom (UK) (*n* = 1), France, (*n* = 1), Israel (*n* = 1), Norway (*n* = 1), and the Republic of Korea (*n* = 1). Most studies (*n* = 52) only included ex-servicewomen in the sample; four studies included both serving and ex-servicewomen, and seven included only serving women. Studies varied in how they defined and measured MST (see Supplementary Materials [S2]).

**Table 1.** Characteristics of included studies: author details, study design, sample description, and quality assessment

(Author, year) C*ountry*	Study design	Study information	N (R.R.)	*n* Female/Women (%)	Mean age of sample in years (SD)	Sample description	Study quality
(Banducci, McCaughey, Gradus, & Street, 2019) *United States*	Cross-sectional	Male and female veterans were recruited from the VHA Environmental Epidemiology Service’s roster of OEF/OIF veterans. Participants completed self-report surveys.	2344 (48.6%)	1207 9(51.5)	35.64 (9.53)	OEF/OIF veterans. 9.2% Marines, 53.6% Army, 14.0% Navy, 0.2% Coast Guard, 0.4% multiple branches. 74.4% Caucasian/ White, 10.6% Hispanic/Latinx, 4.6% Native American/Alaskan Native/Pacific Islander, 3.1% Asian American, 15.1% Black/African American	Fair
(Blais & Geiser, 2019) *United States*	Cross-sectional	Data were extracted from a larger dataset of partnered serving and ex-serving personnel.	1190 (N.R.)	1190 (100)	32.18 (7.58)	Female serving and ex-serving personnel who were partnered at time of participation	Fair
(Blais, Brignone, Fargo, Livingston & Andresen, 2019) *United States*	Cross-sectional	Data were extracted from a larger dataset of partnered female serving and ex-serving military personnel, who had complete data for MST experiences, dependent variables (PTSD, depression, sexual satisfaction and function, suicidal ideation), and covariates. Recruitment for this was through Facebook advertisements and online listservs.	656 (78.8% of original data set)	656 (100)	N.R.	Female serving and ex-serving personnel who were partnered at time of participation	Fair
(Blais et al., 2023) *United States*	Cross-sectional	Data for participants who were aged 18–65 years and partnered at the time of participation were extracted from two gender-specific parent studies with samples of partnered men and women service-members/veterans. These parent studies employed convenience sampling using Facebook advertisements and online listservs.	1389 (N.R.)	833 (59.97)	32.10 (7.44)^a^	Veterans (74.43%) and servicemembers. (76.93% White, 4.68% Black, 0.48% Native American, 6.00% Latina, 9.60% biracial)^a^	Fair
(Breland, Donalson, Yongmei, et al., 2018) *United States*	Cross-sectional	Women veterans were recruited via an urban outpatient VA medical center and associated community outpatient clinics. Eligible participants (aged 18–70, no history of a psychotic disorder or suicide attempt in the past 5 years) completed self-report surveys.	407 (38%)	407 (100)	49 (13)	Veterans. 22% Air Force, 44% Army, 6% Marines, 24% Navy, 4% Other	Fair
(Brownstone, Gerber, Holliman, & Monteith, 2018) *United States*	Qualitative; Semi-structured interviews	Eligible participants (cisgender females with a validated self-reported history of MST (as defined by the VA), aged 18–65 years and eligible to receive care in the local VA healthcare system, able to provide informed consent, not currently experiencing highly severe psychiatric symptoms or significant cognitive impairment) were recruited from local VA medical centers and outpatient clinics to participate in semi-structured interviews. A phenomenological, data-driven methodology was adopted, and data were analyzed using thematic analysis.	32 (N.R.)	32 (100)	N.R.	Cisgender female veterans. 59.4% Caucasian, 18.8% African American, 6.3% Native American, 15.6% Multiracial	Fair
(Bryan, Bryan, & Clemans, 2015) *United States*	Cross-sectional	Eligible participants (military personnel or veteran enrolled in college or university classes in the US) completed an online survey.	464 (N.R.)	115 (29.3)	36.17 (10.25)	Military personnel (35.1%) and veterans (64.9%) who were enrolled in university/college classes. 40.1% Army, 30.6% Air Force, 19.2% Navy, 7.5% Marines, 1.3% Coast Guard. 83.0% Caucasian, 6.3% African American, 3.2% Native American, 2.6% Asian, 1.1% Pacific Islanders, and 10.8% Hispanic/Latinx	Fair
(Chang, Skinner & Boehmer, 2001) *United States*	Cross-sectional	A random sample of eligible participants (women veterans who had received ambulatory care from any of the 158 included hospitals between July 1, 1994, to June 30, 1995) were recruited from the VA Women’s Health Project and completed self-report surveys.	3543 (N.R.)	3543 (100)	**MSA:** 42.7 (11.5) **No MSA:** 48.8 (17)	Women veterans. 51% Army, 21% Navy, 24% Air Force, 6% Marines	Fair
(Cobb Scott et al., 2014) *United States*	Cross-sectional	Eligible participants (females who had separated from OEF/OIF military service and enrolled in VA health care between October 1, 2001, and April 30, 2008, and who lived within 300 miles of the VA New England and VA Indiana regions) were recruited from an ongoing prospective cohort study of OEF/OIF veterans and completed self-report surveys.	365 (11.4%)	365 (100)	**PTSD:** 32.4 (10.5) **No PTSD:** 30.5 (10.3)	Female OEF/OIF veterans. **PTSD:** 75.7% White, 10.0% Hispanic, 10.0% Black, 4.3% Asian/Pacific Islanders. **No PTSD:** 85.9% White, 6.9% Hispanic, 6.1% Black, 1.1% Asian/Pacific Islanders	Fair
(Decker et al., 2021) *United States*	Cross-sectional	Data were extracted from the OEF/OIF/ OND Roster. Eligible participants were OEF/OIF/OND era veterans who first used the VHA between 2008 and 2013, completed the PHQ–9, who did not have evidence of a mental health condition on or before the PHQ–9 administration and who receive care at VHA facilities which reported depression screening rates below 75%.	41,658 (56%)	5116 (12.3)	N.R.	OEF/OIF/OND veterans. 61.3% Army, 13.5% Navy, 7.6% Air Force, 17.6% Marines. 60.8% White, 13.5% Black, 11.7% Hispanic, 13.9% unknown	Fair
(Dutra et al., 2011) *United States*	Cross-sectional	Participants were recruited using convenience sampling in waiting rooms of an Army medical clinic during a post-deployment wellness visit in Oahu, Hawaii following deployment to Iraq. Consenting participants completed assessment interviews before or after their medical appointments.	54 (N.R.)	54 (100)	27.5 (5.8)	Servicewomen. 100% Army. 35.8% Caucasian, 34.0% African American, 15.1% Hispanic, 5.7% Asian/Pacific Islander, 1.9% Native American, 7.5% other	Poor
(Esopenko et al., 2023) *United States*	Cross-sectional	Data were extracted from a larger study of clinical outcomes of trauma-focused care within a 4-week VA acute psychiatric inpatient program (participants were enrolled between December 2009 and March 2013). Eligible participants were female veterans with PTSD diagnoses who had been identified as having limited progress following a less intensive outpatient PTSD treatment.	308 (N.R.)	308 (100)	43.0 (10.5)	Female veterans 13.6% Air Force, 63.3% Army, 0.6% Coast Guard, 11.4% Marines, 11.0% Navy. 45.8% Black, 45.5% White, 3.2% Latina, 1.0% Asian, 4.2% Multiracial, 0.3% other	Fair
(Fillo, Goodell, Homish & Homish, 2023) *United States*	Longitudinal	Data were extracted from an ongoing longitudinal study (Soldiers and Families Excelling Through the Years [SAFETY]), which recruited participants from units across New York State and involved a baseline survey and two annual follow-up surveys. To be eligible, participants were required to be currently serving (at baseline) US Army Reserves or National Guard personnel, part of a couple (married/ living as married) in which one partner was current US Army Reserve or National Guard personnel, who was 18–45 years old. Both partners were required to speak and understand English and to have had at least one alcoholic drink in the previous year.	404 (N.R.)	70 (17.33)	29.9 (5.3)	Army Reserves and National Guard personnel who were currently serving at baseline. 89.0% White, 2.9% Black, 7.1% Hispanic, 7.1% other, 29% no response	Fair
(Fontana & Rosenheck, 1998) *United States*	Cross-sectional	Participants were recruited from VA women’s stress disorder treatment outpatient clinics between May 1, 1994 and January 30, 1997. Eligible participants were women veterans treated in one of the four selected VA clinical programs for women with stress disorders who had complete data for the variables included in the model.	327 (N.R.)	327 (100)	N.R.	Women veterans 50% Army, 21.7% Air Force, 17.4% Navy, 9.3% Marines, 1.6% Coast Guard. 62% European American, 33% African American, 2% Latin American, 3% other	Fair
(Gibson et al., 2019) *United States*	Cross-sectional	Analyses used ICD–9 CM and VA MST codes from clinical visits, obtained from the VA National Patient Care Database electronic medical records. Eligible participants were women veterans aged 55 years or older, enrolled in the VA since 2005, with at least one VA clinical encounter and documented response to MST screening in fiscal years 2005–2015. Only those whose frequency of VA encounters was in the top 10% of the sample were included in analyses.	70,864 (N.R.)	70,864 (100)	65.8 (10.4)	Women veterans. 64% White	Fair
(Gorman et al., 2021) *United States*	Cross-sectional	Participants were recruited from a longitudinal study (Project Veterans After-Discharge Longitudinal Registry [VALOR]). Data were collected using self-report surveys and telephone structured clinical interviews. Eligible participants were OIF/OEF-deployed Army or Marines veterans, who were deployed to combat and who received a VHA mental health evaluation between June 2008 and December 2009.	699 (49.85%)	699 (100)	36.9 (9.6)	Heterosexual (*n* = 647) and sexual minority (*n* = 52) female Army and Marine OIF/OEF-deployed combat veterans. 74.3% White; 23.5% Black, 2.8% Asian, 13.9% Hispanic, 0.7% other/unknown	Fair
(Gradus, Street, Kelly & Stafford, 2008) *United States*	Cross-sectional	Former reservists were recruited via the Defense Manpower Data Center and completed telephone interviews.	3,946 (74.4%)	2328 (59)	39.1 (9.5)	Former reservists. 65% White, 25% Black, and 5% Hispanic	Fair
(Gradus, King, Galatzer-Levy & Street, 2017) *United States*	Cross-sectional	Participants were recruited from the VHA Environmental Epidemiology Service roster and completed self-report surveys. Eligible participants were those deployed in support of the conflicts in Iraq and Afghanistan. Participants were excluded if they reported suicidal ideation with no or few current mental health symptoms or reported more severe suicidal behaviour.	2,161 (48.6%)	1,099 (50.86)	**Suicidal ideation**: 32.97 (8.42)^a^ **No Suicidal ideation:** 34.93 (9.01)^a^	Veterans who deployed in in support of the conflicts in Iraq and Afghanistan. **Suicidal ideation:** (69.8% Army or Marines)^a^ **No suicidal ideation:** (56.2% Army or Marines)^a^	Fair
(Gross et al., 2018) *United States*	Cross-sectional	Data were collected as part of the VA Mid-Atlantic Mental Illness Research, Education and Clinical Center Post Deployment Mental Health Study. Participants were recruited from four VA sites in North Carolina and Virginia and completed self-report surveys and diagnostic interviews. Eligible participants were female Iraq/Afghanistan veterans who completed the MSA assessment.	330 (N.R.)	330 (100)	N.R.	Female Iraq/Afghanistan veterans. 61.5% African American, 38.5% White	Fair
(Gross, Kroll-Desrosiers & Mattocks, 2020) *United States*	Longitudinal	Secondary data analysis of a 3-year longitudinal study. Eligible participants were pregnant women veterans enrolled at one of 15 selected VA medical centers. Data were collected using telephone interviews. Prenatal interviews took place from January 2016. Postnatal interviews took place from July 2016.	363 (38%)	363 (100)	33.22 (4.60)	Female veterans. 59.8% White, 24.7% Black or African American, 2.7% Asian, 1.0% Native Hawaiian or other Pacific Islander, 1.6% American Indian or Alaska Native, 11.6% other	Fair
(Hankin et al., 1999) *United States*	Cross-sectional	Participants were recruited using a random sampling frame from lists provided by 158 VA hospitals. Eligible participants (female veterans, VA patients who had made at least one outpatient visit between July 1, 1994, and June 30, 1995, resided in the community, and had complete addresses) completed self-report surveys.	3632 (56%)	3632 (100)	**Sexual assault:** 42.6 (11.5) **No sexual assault:** 48.8(16.9)	Female veterans. **Sexual assault:** 54.2% Army, 18.5% Navy, 24.1% Air Force, 4.8% Marines, 0.5% Coast Guard. **No sexual assault:** 49.5% Army, 20.6% Navy, 24.0% Air Force, 6.6% Marines, 1.3% Coast Guard	Fair
(Harned & Fitzgerald, 2002) *United States*	Cross-sectional	Eligible participants (active-duty military women, who were posted at one of two US military installations and selected to participate in a DoD gender issues pilot survey) completed self-report surveys.	419 (89%)	419 (100)	27.56 (7.19)	Women veterans. 48% White, 42% Black/African American, 10% Hispanic, 4% Asian/ Pacific Islander, 2% Native American	Fair
(Harned, Ormerod, Palmieri, Collinsworth & Reed, 2002) *United States*	Cross-sectional	Data from the 1995 DoD Gender Issues Survey. Eligible participants were women DoD and Coast Guard members, who were not flag rank officers, with at least 6 months of active-duty service.	22372 (61%)	22372 (100)	31.66 (N.R.)	Servicewomen. 61% White, 27% Black/African American, 8% Hispanic, 3% Asian/Pacific Islander, 1% American Indian	Fair
(Hendrikx, Williamson & Murphy, 2023) *United Kingdom*	Cross-sectional	Surveys were completed by women connected with a women veterans’ charity between August and October 2020. Eligible participants were veterans connected with the charity, who consented to be contacted and provided an email address.	750 (4.60%)	750 (100)	N.R.	Women Army veterans	Fair
(Himmelfarb, Yaeger & Mintz, 2006) *United States*	Cross-sectional	Eligible participants (female veterans) were recruited via the Women’s Comprehensive Healthcare Program at the VA West Los Angeles Healthcare Center or via postal invite to female veterans in the Los Angeles metropolitan area between December 2000 and December 2002. Participants completed self-report questionnaires and structured interviews.	196 (25%)	196 (100)	48 (15)	Female veterans. 43.9% Army, 25%, 24% Navy, 6.6% Marines. 39.5% White, 39.0% African American, 11.7% Latina, 3.1% Asian, 2.6% Aleutian, Eskimo/American Indian	Fair
(Hoffmire et al., 2021) *United States*	Cross-sectional	Analyses used baseline data from a longitudinal study (collected between 2012 and 2015) focused on post-deployment experiences and coping among previously deployed post–9/11 era veterans. Recruitment targeted those who had separated from the military in the past 5 years. Participants completed self-report surveys.	809 (N.R.)	330 (40.79)	N.R.	Previously deployed post–9/11 era veterans. 61.5% Army, 18.6% Air Force, 19.8% Marines/ Navy; 30.2% reserves and 20.1% were National Guard. 67.8% White, 16.9% Black, 15.4% other	Good
(Kang, Dalager, Mahan & Ishii, 2005) *United States*	Nested case–control analysis	Analysis of data collected for the National Health Survey of Gulf War Era Veterans and Their Families (a population-based survey collected in 1996). Eligible participants were US veterans who served during the Gulf War. Gulf War veterans with current PTSD (*n* = 1381) were compared to Gulf War veteran controls without PTSD (*n* = 10,060).	11, 441 (38.14%)	2131 (18.63)	**PTSD:** 39.1 (N.R.) **No PTSD:** 38.1 (N.R.)	Gulf War Veterans. **PTSD:** 46.7% White. **No PTSD:** 63.2% White	Good
(Katz, Huffman & Cojucar, 2017) *United States*	Qualitative; Semi-structured interviews	Eligible participants (women veterans with a history of MSA whilst on active duty, who were engaged in psychotherapy and had a psychotherapist available post-interview, and who had no active psychosis, substance abuse, suicidal ideation or hospitalisation for suicidal ideation in the past 6 months) self-selected to participate in a semi-structured interview about MSA, consisting of structured and open-ended questions. Structured data was analysed via summation of similar responses and open-ended data was analysed using content analysis using coding categories	21 (N.R.)	21 (100)	21.05 (3.18)	US women veterans who served during 1966–2001 in the Army (*n* = 8), Navy (*n* = 6), Air Force (*n* = 4), and the Marines (*n* = 3). Caucasian (*n* = 10), African American (*n* = 6), Hispanic (*n*–3), American Indian (*n* = 1), mixed heritage (*n* = 1)	Poor
(Kearns et al., 2016) *United States*	Cross-sectional	Self-report questionnaires and interviews completed as follow up for Project Veterans After-Discharge Longitudinal Registry (VALOR). Eligible participants (Army or Marines veterans, who had completed a mental health evaluation at a VA facility) completed self-report questionnaires. Participants were recruited from a roster provided by the VA Environmental Epidemiology Service. Veterans with probable PTSD were oversampled.	673 (60.8%)	673 (100)	36.9 (9.6)	Female Marine/Army veterans. 74.3% White, 23.5% Black, 13.9% Hispanic, 2.8% Asian, 0.7% other/unknown	Good
(Kim, Lee, Lee, Han & Park, 2017) *Republic of Korea*	Cross-sectional	Data from a military health survey conducted in 2014.	228 (N.R.)	228 (100)	N.R.	Female military personnel, currently serving. 65.8% Army, 23.7% Navy, 10.5% Air Force	Poor
(Kimerling, Gima, Smith, Street & Frayne, 2007) *United States*	Cross-sectional	Data analysis of VHA administrative data (ICD–9 codes) from a national sample of VHA outpatients. Eligible participants were VHA outpatients who had at least one outpatient visit to a VHA facility that reported valid MST monitoring data during fiscal year 2003.	3035000 (N.R.)	134894 (4.5)	N.R.	Veterans who were VHA outpatients. **MST:** (49.1% White, 15.9% Black, 2.2% Hispanic, 0.8% other, 32.0% unknown)^a^. **No MST:** (39.9% White, 13.3% Black, 1.5% Hispanic, 0.5% other, 44.8% unknown)^a^	Fair
(Kimerling et al., 2010) *United States*	Cross-sectional	Data were extracted from VA electronic medical records. Eligible participants were veterans deployed on OEF/OIF and separated from military service by September 30, 2006, who used VHA mental health or primary care services between October 1, 2001 and September 30, 2007 and were screened for MST.	125, 729 (80.5%)	17580 (13.98)	N.R.	OEF/OIF veterans. **MST:** (59.3% White, 25.5% Black, 10.0% Hispanic, 5.2% other/unknown)^a^. **No MST:** (49.6% White, 34.6% Black, 10.0% Hispanic, 5.7% other/unknown)^a^	Fair
(Laws, Mazure, McKee, Park & Hoff, 2016) *United States*	Cross-sectional	Data from the Survey of Experiences of Returning Veterans’ structured phone interviews collected within 5 years of separation from service.	818 (N.R.)	328 (40.1)	35.10 (8.78)	Veterans. 62% Army, 14% Air Force, 13% Marines, 11% Navy. 78% White, 15% Black or African American, 4% Asian, 1% Native Hawaiian or other Pacific Islander, 5% American Indian or Alaska Native; 14% Hispanic/Latinx	Good
(Lindsay et al., 2016) *United States*	Cross-sectional	Analysis of VA administrative data (ICD–9 codes) from the VHA Medical Statistical Analysis System file, covering VHA inpatient and outpatient medical and mental health treatment (from October 1, 2000, to September 30, 2013). Eligible participants were transgender veterans in the VHA, who served during OEF/OIF conflicts.	332 (N.R.)	254 (76.5%)	33.98 (8.41)^a^	Transgender veterans. (79.9% White, 19.7% ethnic minority, 0.4% declined to answer)^a^	Fair
(Luterek, Bittinger & Simpson, 2011) *United States*	Cross-sectional	Participants were recruited from the VA Puget Sound Health Care System outpatient general mental health clinic and specialized mental health clinic for PTSD. Eligible participants (enrolled in VA Puget Sound Health Care System for at least 12 months, had at least two mental health visits in the past year, were actively followed in the general mental health or PTSD clinic at the time of assessment, were determined as stable by clinicians, and were not engaged in addictions treatment or the outpatient clinic serving veterans with severe chronic mental illness) completed self-report surveys and interviews.	208 (42%)	104 (50%)	48.6 (9.8)^a^	Veterans. (74% Caucasian, 10.6% African American, 2.9% Asian American, 1.9% Hispanic)^a^	Fair
(Maguen et al., 2012) *United States*	Cross-sectional	Retrospective data analyses conducted using VA administrative data. Eligible participants were Iraq and Afghanistan veterans, with at least one primary care or mental health visit to a VA facility between April 1, 2002 and October 1, 2008 and who were new users of VA health care	213,803 (N/A)	26527 (12.41)	N.R.	Iraq and Afghanistan veterans. 65% Army, 4% Marines, 16% Navy, 15% Air Force. 49% White, 25% Black, 15% Hispanic, and 11% other	Fair
(Mahoney, Shayani & Iverson, 2024) *United States*	Longitudinal	Self-report surveys completed at Time 1 and Time 2 (12 months later). Participants were recruited from a larger survey study. To be eligible, participants had to be female, aged at least 18 years of age, and use VHA care in the New England region within the prior year.	198 (79.8%)	198 (100)	51.01 (16.93)	Female veterans. 46.1% Army, 20.2% Navy, 6.7% Marines, 1.6% Coast Guard. 77.8% White, 9.1% Black/ African American, 4.6% American Indian/Alaska Native, 0.5% Native Hawaiian/Pacific Islander, 0.5% Asian; 3.5% Hispanic/Latina/Spanish origin	Good
(Mercado, Ming Foynes, Carpenter & Iverson, 2015) *United States*	Cross-sectional	Participants were recruited (~ fiscal year 2011) from a random sample of female veterans who were VA patients in the New England region. Eligible participants (women, aged at least 18 years, enrolled as a veteran patient (not a dependent) in the VA New England Healthcare System, and had attended one or more VA medical or mental health appointments within the last year) completed self-report paper-and-pencil mail survey	369 (63.5%)	369 (100)	**No MST:** 58.4 (18.9) **Non-IPV-MST**: 52.1 (14.8) **IPV-MST:** 54.0 (12.4)	Women veterans. **No MST:** 46.9% Army, 2.8% Marines, 28.3% Navy, 19.2% Air Force 2.8% Coast Guard. **Non-IPV-MST:** 49.0% Army, 6.7% Marines, 20.8% Navy, 22.2% Air Force, 1.3% Coast Guard. **IPV-MST:** 44.0% Army, 8.0% Marines, 28.0% Navy, 20.0% Air Force, 0% Coast Guard	Fair
(Monteith et al., 2018) *United States*	Cross-sectional	Secondary analysis of baseline interview data from the Survey of Experiences of Returning Veterans. Eligible participants were veterans separated from the US military, who served in OEF/OIF/OND, aged 18 or older, English-speaking, and currently living in the US.	824 (N.R.)	344 (41.7)		Veterans. 73.2% White, 12.7% Black, 14.1% other	Fair
(Monteith et al., 2023) *United States*	Cross-sectional	Secondary analysis of survey data. Eligible participants were women veterans, separated from military service between October 1, 2009 and September 30, 2018, of reproductive age (18–44 years) at separation, who had used VHA provided reproductive healthcare in fiscal year 2018.	352 (17.9%)	352 (100)	34.07 (6.69)	Women veterans . 46.86% Army, 24.57% Air Force, 19.60% Navy/Coast Guard (19.60%), 10.86% Marines. 66.38% White, 15.67% Black, 1.99% Native American/Alaskan Native, 3.13% Asian, 0.85% Pacific Islander, 8.55% Multiracial 3.42% other	Fair
(Moreau et al., 2022) *France*	Cross-sectional	Analysis used data from a national sexual health survey in the French military (conducted in 2014–2015). Recruitment employed two-stage random sampling strategy, where 18 military units were randomly selected. 120 individuals per unit were randomly selected and invited by the unit commander to attend an information session.	1500 (76%)	232 (15.47)	30.6 (N.R.)^a^	Currently serving personnel. 39.6% Army, 43.6% Air Force, 16.8% Navy	Fair
(Murdoch et al., 2006) *United States*	Cross-sectional	Veterans who had previously filed VA PTSD disability benefit claims were sent self-report surveys approximately 2 years after filing claims.	3337 (68%)	1682 (50.4)	48.4 (12.6)	Veterans. ~75% Caucasian	Good
(Murdoch et al., 2010) *United States*	Cross-sectional	Participants were recruited from the VA enrolment database and invited to complete self-report questionnaire. Eligible participants were those confirmed as active duty between January 1998 and June 2002 in a VA database.	611 (84%)	(38)	N.R.	Veterans. 52% Army, 32% Air Force, 11% Navy, 4% Marines. 66% White, 20%, Black or African American; 6% other race; 12% Hispanic/ Latinx	Fair
(Murray-Swank, Dausch, & Ehrnstrom, 2018) *United States*	Cross-sectional	Eligible participants (women veterans who had a ‘rural’ or ‘highly rural’ zip code, availability to attend one wellness retreat, and psychological capacity to participate in a residential, wellness-based program, with no acute medical health conditions (e.g. need for oxygen, severe heart condition), acute suicidality (within past month), and current drug and/or alcohol abuse) were sent self-report surveys to complete.	101 (18%)	101 (100)	48.6 (9.26)	Women veterans. 80% Caucasian/White, 10% Hispanic or Latina, 9% Black or African American 9%, 1% Native, American/Alaskan Native, 1%, Asian/Pacific Islander	Poor
(Newins et al., 2021) *United States*	Cross-sectional	Participants were recruited for the Post-Deployment Mental Health Study via mailings, VA clinician referrals, and advertisements. Participants completed self-report surveys, which were completed in four VA medical centers in Virginia and North Carolina between 2005 and 2015. Eligible participants were Iraq/Afghanistan-era US military veterans, active-duty military personnel, and members of the Reserves and National Guard who served post–9/11, whose primary language was English, who could comprehend an informed consent form and process, and could travel to one of the data-collection sites. Individuals were not required to be seeking mental health treatment (or any health care services).	3114 (N.R.)	611 (19.6)	N.R.	Military personnel and veterans. 70.5% Army, 17.3% Navy, 2.3% Air Force, 6.1% Coast Guard; 34.7% were in the Reserves/ National Guard, 6.1% Active Duty. 33.9% White, 63.3% Black, 2.8% other	Fair
(Reinhardt, McCaughey, Vento & Street, 2023) *United States*	Qualitative; Semi-structured interviews	Eligible participants (women-identifying veterans who had experienced sexual harassment or sexual assault during military service) were recruited via flyers in a VHA located in a Northeastern US city. Participants completed semi-structured interviews, which were analyzed using a grounded theory-informed thematic analytic approach.	23 (N.R.)	23 (100)	51 (N.R.)	Women veterans. 13% Air Force, 9% Army National Guard, 4% Coast Guard, 39% Army, 26% Navy. White (*n* = 14), African American (*n* = 3), Native American (*n* = 2), Mexican American (*n* = 1), Multiracial (*n* = 1), other (*n* = 1)	Excellent
(Rønning et al., 2024) *Norway*	Cross-sectional	Secondary analysis using data from a post-deployment survey. Eligible participants were Norwegian military personnel who had deployed to Afghanistan between 2001 and 2020.	6205 (67.7%)	512 (8.25)	42.0 (10.0)^a^	Veterans	Fair
(Sandhu, Dougherty & Haedt-Matt, 2022) *United States*	Cross-sectional	Self-report surveys. Recruitment conducted via Prolific. Eligible participants were US veterans who identified as female.	98 (N.R.)	98 (100)	39.03 (10.19)	Female veterans. 69.4% White/Caucasian, 17.3% Black/African American, 4.1% Hispanic/Latino, 7.1% Biracial, 2.0% other	Poor
(Skinner et al., 2000) *United States*	Cross-sectional	Data extracted from the VA Women’s Health Project. Eligible participants (female veterans who had at least one ambulatory visit at any VA facility between July 1, 1994 and June 30, 1995) were recruited via postal invite.	3632 (58.40%)	3632 (100)	**Sexual harassment:** 42.8 (12.5) **No sexual harassment:** 53.0 (8.0), **Sexual assault:** 42.6 (11.5) **No sexual assault:** 48.8 (16.9)	Female veterans. **Sexual harassment:** 75.3% White, **No sexual harassment:** 76.7% White **Sexual assault:** 76.8% White **No sexual assault** 75.8% White	Fair
(Smith, Wang, Vaughn-Coaxum, Di Leone & Vogt, 2017) *United States*	Cross-sectional	Participants were recruited from a roster from the Defense Manpower Data Center, stratified by component of service (50% active-duty component and 50% National Guard/Reservist) and gender, with oversampling of women. Eligible participants (veterans who had returned from deployment to Iraq or Afghanistan within the previous 2 years [2007–2009]) competed self-report surveys.	469 (47%)	(59.1)	35.27 (10.81)	Veterans. 67.0% Army, 12.3% Navy, 14.0% Air Force, 6.7% Marines; 53.4% active duty, 26.5% National Guard, 20.2% Reserves. 72.7% White, 15.8% African-Americam,3.6% Asian, 2.6% American Indian/ Alaskan Native, 1.3% Pacific Islander/ Native Hawaiian; 11.9% Hispanic	Fair
(Smith, Brady, Hammer, Carlson & Mohr, 2020) *United States*	Longitudinal	Participants were recruited from a larger Randomized Control Trial Eligible participants worked at one of the 35 organisations participating in the Randomized Control Trial for at least 20 hours per week and had served in the US military post–9/11 era. Self-report surveys were completed at baseline and two additional follow-ups (3 months and 9 months).	67 (N.R.)	67 (100)	37.98 (9.50)	Veterans. 15% Army national guard, 10% Air Force National Guard, 13% Army Reserves, 7% Marine Reserves, 22% Navy Reserves, 8%, Air Force reserves, 1% Coast Guard Reserves, 16% Army, 8% Air Force. 77% White, 3% Black/ African American, 3% Asian, 3% American Indian/ Alaska Native, 1% Hispanic, 15% multiple racial/ethnic identities	Good
(Stefanovics, Potenza, Tsai, Nichter & Pietrzak, 2023) *United States*	Cross-sectional	Eligible participants (US veterans) were recruited from the National Health and Resilience in Veterans Study and completed self-report surveys.	4069 (N.R.)	505 (9.8)	62.2 (15.7)	Veterans. 49.7% Army, 21.2% Navy, 19.6% Air Force, 6.5% Marines. 78.1% White	Fair
(Street, Stafford, Mahan & Hendricks, 2008) *United States*	Cross-sectional	Participants were recruited through a stratified (based on gender and reserve component) random sampling design from a list of former reservists provided by the Defense Manpower Data Center. Eligible participants (former reservists who had not also served in the active-duty forces) completed self-report, computer-assisted telephone interviews.	3,946 (74.4%)	2318	N.R.	Former reservists. **Sexual harassment/ assault:** (67.9% White, 22.5% Black/ African American, 5.4% Hispanic/Latino, 4.2% other)^a^. **No sexual harassment/ assault:** (63.7% White, 26.7% Black/ African American, 4.3% Hispanic/Latino, 5.3% other)^a^	Fair
(Sumner et al., 2021) *United States*	Cross-sectional	Analyses of VHA electronic medical records. Eligible participants were women veterans aged 18 years or older, who enrolled in the VHA between January 1, 2000 and December 31, 2017, had at least one VHA clinical encounter during the observation period, and completed the MST screen.	502,199 (N.R.)	502199 (100)	40.0 (15.6)	Women veterans 20.5% Air Force, 49.2% Army, 6.4% Marine Corps, 21.0% Navy, 1.1% other. 58.8% White, 27.5% Black, 1.8% Asian, 1.4% American Indian or Alaska Native, 1.2% Native Hawaiian or other Pacific Islander, 9.2% Unknown, 7.8% Hispanic/ Latina, 84.8% not Hispanic/ Latina, 7.4% unknown	Fair
(Surís, Lind, Kashner, Borman & Petty, 2004) *United States*	Cross-sectional	Eligible participants (female veterans enrolled in a medical and/or mental health clinic within the VA North Texas Health Care System who were seen for at least one outpatient appointment during the 5 years before contact) were recruited via the Dallas VA Medical Center and mental health clinical within the center (1997–2000). Analyses used data from participant interviews and medical records.	270 (70%)	270 (100)	46.69 (11.52)	Female veterans. 46.7% Army, 30.4% Air Force, 17.4% Navy, 5.2% Marines, 1.04% Coast Guard	Fair
(Surís, Lind, Kashner & Borman, 2007) *United States*	Cross-sectional	Eligible participants (female veterans who were enrolled in a medical or mental health clinic within the North Texas VA and attended at least one outpatient appointment during the 5 years before contact) were recruited through a VA outpatient medical center via clinician referrals, responses to in-hospital advertisements and appointment schedules. Participants completed self-report questionnaires and interviews.	270 (70%)	270 (100)	46.7 (11.5)	Female veterans. 64.1% Caucasian	Fair
(Webermann, Relyea, Portnoy, Martino, Brandt & Haskell, 2023) *United States*	Longitudinal	Participants were recruited from a DoD roster between February 11, 2016 and October 28, 2019, and completed self-report surveys. Eligible participants were veterans with English literacy and who served in post–9/11 conflicts, who completed both baseline and follow-up surveys and had no missing data for the primary outcome variable (PTSD symptoms at follow-up).	825 (72.0%)	428	41.61 (10.27)^a^	Veterans. 62.0% Army, 17.4% Air Force, 13.8% Navy, 6.8% Marines. 84.9% White, 8.4% Black or African American, 3.2% multiracial, 2.4% Asian, 0.9% American Indian/ Alaskan Native, 0.2% Native Hawaiian/Pacific Islander	Fair
(Wilson et al., 2020) *United States*	Cross-sectional	Data from the VA Mid-Atlantic Post-Deployment Mental Health study. Eligible participants (veterans who served in the US military post–9/11 and completed MSA measures) completed self-report surveys and clinician interviews.	1571 (N.R.)	330 (21)	N.R.	Veterans. 48.9% Black, 49.9% White; 93.6% non-Hispanic/Latinx ethnicity	Fair
(Wolfe et al., 1998) *United States*	Cross-sectional	Participants were recruited from the Ft. Devens Operation Desert Storm Reunion Survey and completed additional self-report surveys.	160 (66.7%)	160 (100)	28.2 (6.8)	Women Army veterans. 74.4% Caucasian, 14.4% African American, 2.5% Hispanic, 8.8% other	Good
(Yaeger, Himmelfarb, Cammack & Mintz, 2006) *United States*	Cross-sectional	Participants were recruited from the Women’s Comprehensive Healthcare Center at the West Los Angeles VA or via letter (between December 2000 and December 2002). Eligible participants (veteran presenting to the clinic for treatment, who did not have dementia, psychosis or suicidality) completed self-report surveys and interviews.	196 (N.R.)	196 (100)	47.8 (14.5)	Women veterans. Most of the sample had served in the Army	Fair
(Yalch, Hebenstreit & Maguen, 2018) *United States*	Cross-sectional	Participants were recruited from a VHA hospital and surrounding outpatient clinics as part of a larger study of female veterans’ health. Eligible participants (female with an address within VHA catchment area, aged between 18 and 70 years, no current psychosis or history of psychosis, and no suicide attempt within the last 5 years) completed self-report surveys.	407 (N.R.)	407 (100)	49 (13)	Female veterans. 43% Army, 24% Navy, 22% Air Force, 6% Marines, 4% other 59% Caucasian/White, 14% Black/African American, 8% Latina, 8% multi-racial, 7% Asian, and 3% other	Fair
(Zelkowitz, Sienkiewicz, Vogt, Smith & Mitchell, 2022) *United States*	Cross-sectional	Eligible participants (veterans with valid addresses) were recruited from the VA DoD Identity Repository and completed self-report surveys.	1187 (29%)	594 (50)	47.44 (13.38)^a^	Veterans (70.67% White, 21.26% Black/ African American, 3.79% American Indian/ Alaskan Native, 2.58% Asian American, 1.49% Hawaiian/ Pacific Islander, 5.39% other; 8.37% Hispanic)^a^	Fair
(Zerach, 2023) *Israel*	Cross-sectional	Eligible participants (18 years of age, served mandatory service in the Israeli Defense Force, and released from military service within the last 20 years) completed self-report surveys. Participants recruited between January and April 2022 via combat veterans’ websites and communities, Ariel University, and online adverts and social media campaigns.	1613 (81%)	1613 (100)	**Combat veterans:** 26.00 (4.80) **Non-combat veterans:** 26.06 (4.63)	Women combat (*n* = 885) and non-combat (*n* = 728) veterans	Fair

*Note*: DoD, Department of Defense; ICD-9, International Classification of Diseases 9th Revision; ICD-9 CM, International Classification of Diseases 9th Revision, Clinical Modification; IPV-MST, Intimate Partner Violence- related MST; Non-IPV-MST, Non-Intimate Partner Violence-related MST; N/A, not applicable; N.R., not reported; OEF, Operation Enduring Freedom; OIF, Operation Iraqi Freedom; OND, Operation New Dawn; PHQ-9, Patient Health Questionnaire; SD, standard deviation; VA, Department of Veterans Affairs; VHA, Veterans Health Administration.

a Values specific to women/ females in mixed samples.

### Quality assessment

Most studies were rated as ‘fair’ quality (*n* = 49), nine as ‘good’/ ‘excellent’ and five as ‘poor’. Reasons for lower quality ratings often included not accounting for covariates in analyses, not presenting clear study aims, and not clearly defining exposure measures.

### Quantitative studies

Table 2 presents the main findings from quantitative studies. Information on descriptive statistics and mental health measures is available in the Supplementary Materials (S3).

**Table 2.** Quantitative mental health findings

Reference	Outcome(s)	Covariates	Main findings
(Banducci et al., 2019)	PTSD symptom severity, alcohol use severity	N/A	MST severity was significantly associated with PTSD (Est. *= 2.63*, SE *= 0.20, p < .001*) and alcohol use severity (Est. *= 0.04*, SE *= 0.01, p < .002*). The association between MST and alcohol use severity was mediated by PTSD symptom severity (*b = 0.038*, CI *= 0.027, 0.051).* Life disruptions at home during deployment increased the impact of MST on PTSD severity (Est. *= 2.08*, SE *= 0.2, p < .001)* and the pathway between MST and alcohol use (via PTSD) was exacerbated by life disruptions *(*Est. *= 0.0007*, SE *= 0.0001*, CI *= 0.0002, 0.0013*).
(Blais & Geiser, 2019)	SI	Age, race, marital status, army service, veteran status and deployment history	Significant indirect paths between MST-assault and SI via depression severity *(*Est. *= 0.19, 95%* CI *= [0.12, 0.28], Standardized* Est. *= 0.11)*, and avoidance *(*Est. *= − 0.12, 95%* CI *= [−0.23, −0.02], Standardized* Est. *= − 0.07)*, and anhedonia (Est. *= 0.10, 95%* CI *= [0.01, 0.20], Standardized* Est. *= 0.06)* symptom clusters were observed. The avoidance symptom cluster variable was interpreted to have acted as a suppressor. No significant mediated paths between MST harassment and SI were found. The path model could explain 29% of the variance.
(Blais et al., 2019)	PTSD severity and probable PTSD, depression severity and probable depression, SI	Marital status, history of deployment, white race, service in the army	Harassment-only MST was significantly associated with higher PTSD symptoms *(β = 5.69*, SE *= 2.29, p < .05)* but not probable PTSD. Assault-MST was significantly associated with PTSD severity (*β = 24.93*, SE *= 2.42, p < .001*). and probable PTSD *(*OR *= 6.66*, CI *= 3.86–11.49, p < .001).* Harassment-only MST was not significantly associated with depression severity or probable depression. Assault MST was associated with depression severity *(β = 5.07*, SE *= .80, p < .001*) and probable depression *(*OR *= 3.67*, CI *= 2.26–5.94, p < .001).* Assault-MST, but not Harassment-only MST, was associated SI (OR *= 3.62*, CI *= 1.93–6.78, p < .001).*
(Blais et al., 2023)	PTSD severity and probable PTSD, depression severity and probable depression, SI	Marital status, age, and discharge status	MSV *(*OR *= 3.86, p = .001)* and revictimisation *(*OR *= 3.67, p = .001)* were each significantly associated with presence of PTSD symptoms compared to no sexual trauma. MSV (*B = 13.21, p = .002)* and revictimisation *(B = 10.03, p = .016)* were each significantly associated with higher PTSD symptoms compared to no sexual trauma. Revictimisation compared to no sexual trauma was significantly associated with >1 depression symptoms *(*OR *= 2.45, p = .047).* MSV-only was not significantly associated with probable depression. MSV compared to no sexual trauma was significantly associated with higher depression symptoms *(B = 2.61, p = .047).* MSV (OR *= 1.49, p = .047)* and revictimisation *(*OR *= 1.41, p = .030*) compared to no sexual trauma were each associated with SI. MSV (OR *= 3.10, p = .005)* and revictimisation (OR = 2.94, p = .005) were associated with risk of probable PTSD compared to pre-military sexual violence. No other comparisons were significant.
(Breland, Donalson, Yongmei, et al., 2018)	Probable eating disorder	Age, race and service branch	MST was associated with meeting criteria for a probable eating disorder (bulimia nervosa, anorexia nervosa or binge eating disorder, grouped as a single outcome variable) *(*OR *= 2.03; 95%* CI *[1.03–3.98]).*
(Bryan et al., 2015)	Suicidal ideation, suicide planning, suicide attempts	Age	MST did not have significant associations with any of the outcome variables.
(Chang et al., 2001)	General mental health status, depressive symptoms	Social support, age and race	Women with experiences of MSA had significantly lower general mental health *(M = 35.5*, SD *= 12.5)* than women who did not report experiences of MSA *(M = 42.6*, SD *= 12.6, p < .001)* and higher depressive symptoms. *(M = 10.7*, SD *= 5.5)* than women who did not report experiences of MSA *(M = 7.2*, SD *= 5.3, p < .001).* There was a significant interaction, where religious attendance decreased the impact of MSA on general mental health *(F = 2.89*, DF *= 4, p = .02)* and depression *(F = 3.95*, DF *= 4, p = .003).* The interactions between religious strength/ comfort and MSA were not significant.
(Cobb Scott et al., 2014)	Military-related PTSS	Age, ethnicity, education, marital status and duty type (active-duty vs National Guard/Reserve)	MST significantly predicted combat related PTSS S *(β = .27, t = 5.89, p < .001).* MST and combat exposure significantly interacted to predict greater combat-related PTSS *(β = .14, t = 2.18, P = .030).*
(Decker et al., 2021)	SI	Race, age, marital status, and branch of service	MST increased risk of SI *(*RR *= 1.65, 95%* CI *[1.35, 2.00]).*
(Dutra et al., 2011)	Past week depression symptoms, past week PTSD symptoms	N/A	Sexual harassment and PTSD were significantly correlated (*r = .38, p < .05).* No significant correlation between sexual harassment and depression was observed. Sexual harassment significantly predicted PTSD symptoms *(β = .39, p < .01)* but did not account for significant variance in depression symptoms.
(Esopenko et al., 2023)	PTSD symptoms, depressive symptoms, suicidality in the week prior to admission	General linear modeling analyses controlled for combat deployment experiences and analysis of covariance controlled for adverse childhood experiences	Exposure to MST and IPV was associated with higher PTSD symptoms *(M = 65.6*, SD *= 12.8)* compared to exposure to only IPV *(M = 57.4*, SD *= 18.0, p = .019)* or no trauma exposure *(M = 58.7*, SD *= 12.6; p = .017).* Those who reported MST showed greater symptoms of PTSD than those with no trauma *(M = 65.0*, SD *= 13.2, p < .05).* Exposure to both MST and IPV showed significantly higher depression symptoms (*M = 37.2*, SD *= 12.1)* compared to participants who reported no trauma *(M = 30.3*, SD *= 11.5, p = .013).* Current suicidal ideation and past suicide attempts did not differ by interpersonal trauma types.
(Fillo et al., 2023)	Past-year alcohol problems	Age, PTSD symptoms, and alcohol consumption	No significant relationship was observed between MST history and alcohol problems.
(Fontana & Rosenheck, 1998)	Current PTSD symptom severity	Education, age of entry into military service, service era, duty-related stress, pre-military sexual stress, African American ethnicity, post-military social support, and post-military sexual stress	Sexual stress during military service was 3.5 times more influential in the development of PTSD than duty-related stress and could explain 34.96% of variance in the model.
(Gibson et al., 2019)	PTSD, depressive disorders, anxiety disorders, AUD, SUID, opioid use disorder, and SI	Age, race/ethnicity, and marital status	MST was associated with higher odds of: PTSD (OR *= 7.25*, CI *= (6.84, 7.68), p < .001*). Anxiety (OR *= 1.99*, CI *= (1.89, 2.09), p < .001).* Depression (OR *= 2.39*, CI *= (2.28, 2.50), p < .001).* AUD (OR *= 1.71*, CI *= (1.57, 1.86), p < .001).* SUD *(*OR *= 1.88*, CI *= (1.69, 2.10), p < .001).* SI *(*OR *= 2.42*, CI *= (2.08, 2.82), p < .001).*
(Gorman et al., 2021)	PTSD and MDD diagnosis over the last 30 days	Age, gender and race/ethnicity	MSA was significantly associated with MDD diagnostic status *(*AOR *= 1.33, p < .005)* and MDD *(b = .177, p < .001)* symptom severity amongst heterosexual female participants. MSA was associated with higher PTSD symptom severity *(b = .237, p < .001)*, but not PTSD diagnostic status amongst heterosexual female participants. MSA was not associated with any of the mental health outcomes in sexual minority female participants.
(Gradus et al., 2008)	Current problem drinking behavior, current depression symptoms	N/A	Sexual harassment predicted increased risk of harmful alcohol use *(B = 0.02*, Wald *= 6.62, p < .01)* and depression symptoms *(B = 0 .11, t = 12.33, p < .001).* The association between MST and harmful alcohol use was mediated by depression symptoms *(B = 0 .087*, Wald *= 38.99, p < .001).*
(Gradus et al., 2017)	Post-military SI	N/A	Classification tree analyses found that the highest probability of SI was amongst women who had probable depression, had been sexually harassed during deployment, and had probable PTSD. Women with probable depression and who were sexually harassed during deployment had a moderate probability of SI.
(Gross et al., 2018)	PTSD symptom severity, lifetime and current PTSD diagnoses	N/A	MSA significantly increased PTSD symptom severity (*b = .28, p < .001).* MSA predicted lifetime PTSD diagnosis *(*OR *= 6.48, p < .001, 95%* CI *[2.57, 16.32])* and current PTSD diagnosis *(*OR *= 6.46, p < .001, 95%* CI *[3.01, 13.86]).* The interaction effects between MSA and combat exposure were not significant.
(Gross et al., 2020)	Pre- and postnatal depression symptom severity, prenatal, and postnatal SI	Mediation analyses controlled for prenatal stress, race (white vs minority), age, sexual orientation (heterosexual vs minority sexual orientation), marital status, disability status)	MSH predicted higher postnatal depression *(B = 1.89 (*SE *= 0.48), p < .001).* The relationship between MSH and postnatal depression was mediated by prenatal depression, *(B = 1.11 (*SE *= 0.26), p < .001, 95%* CI *[0.65–1.67], Sobel test: z = 4.44, p < 0.001).* MSA predicted postnatal depression *(B = 1.66 (*SE *= 0.55), p < .01).* The relationship between MSA and postnatal depression was mediated by prenatal depression *(B = 1.50 (*SE *= 0.35), p < .001, 95%* CI *[0.89, 2.22], Sobel test: z = 5.08, p < 0.001).* MSH was associated with significantly higher prenatal depression (M = 8.42, SD = 6.61 vs. M = 4.55, SD = 4.83; p ≤ .001), post-natal depression (M = 6.38, SD = 6.22 vs M = 3.49, SD = 4.25; p ≤ .001), and prenatal SI *(M = 0.72*, SD *= 0.88 vs M = 0.34*, SD *= 0.61; p ≤ .001)* but not postnatal SI. MSA was associated with significantly higher prenatal depression *(M = 9.55*, SD *= 7.10 vs M = 5.27*, SD *= 5.12; p ≤ .001)*, postnatal depression (M = 7.14, SD = 6.82 vs M = 4.09, SD = 4.60; p ≤ .001), and prenatal SI *(M = 0.82*, SD *= 0.94 vs M = 0.42*, SD *= 0.67; p ≤ .001)* but not postnatal SI.
(Hankin et al., 1999)	Current symptoms (prior month) of depression and current alcohol use	Analyses controlled for age and educational level	Experiencing sexual assault during military service was associated with probable depression *(*OR *= 3.16, 95%* CI *= 2.68, 3.72)* and alcohol abuse (OR *= 1.89, 95%* CI *= 1.27, 2.60*).
(Harned & Fitzgerald, 2002)	Eating disorder symptoms	N/A	The link between workplace sexual harassment and eating disorders was mediated by psychological distress. The direct pathway between sexual harassment and eating disorder symptoms was not significant. The total effect of sexual harassment on eating disorder symptoms in this model was .03.
(Harned et al., 2002)	Psychological wellbeing	N/A	Sexual assault *(r = −.29, p < .01)* and sexual harassment *(r = −.27, p < .01)* were negatively correlated with psychological well-being. Cross validation models found that sexual harassment *(LISREL path coefficient = −.06, p < .05) and* sexual assault *(LISREL path coefficient = −.18, p < .05)* were associated with poorer psychological wellbeing.
(Hendrikx et al., 2023)	CMD, PTSD, somatisation, harmful alcohol use	Sociodemographic and military factors	Sexual harassment was associated with meeting the criteria for PTSD *(*OR *= 2.30, p = 0.007, 95%*CI *1.25 to 4.21)* and high physical somatisation *(*OR *= 2.58, p = 0.003, 95%* CI *1.38 to 4.81).* Sexual assault was associated with meeting the criteria for PTSD (OR *= 2.73, p = 0.026, 95%* CI *1.13 to 6.61)* and harmful alcohol use *(*OR *= 2.88, p = 0.016, 95%* CI *1.22 to 6.80).* Neither sexual harassment not sexual assault was significantly associated with CMD.
(Himmelfarb et al., 2006)	PTSD	N/A	MST was significantly associated with PTSD, (OR *= 4.30, 95%* CI *= 2.30–8.00, p < .001).*
(Hoffmire et al., 2021)	Recent active suicidal ideation (past 3 months)	Age, race and marital status.	Deployment Sexual Trauma was significantly associated with suicidal ideation (PR *= 1.47, 95%* CI *[1.24, 1.75], p < .05).*
(Kang et al., 2005)	PTSD	Age, race, military rank, unit type, branch of service, and combat exposure	Sexual harassment *(*aOR*, 2.52; 95%* CI*, 1.91–3.33)* and assault *(*aOR*, 5.41; 95%* CI*, 3.19–9.17)* were significantly associated with PTSD.
(Kearns et al., 2016)	PTSD and MDD diagnostic statuses (past 30 days), PTSD symptom severity, MDD symptom severity	Demographic variables	MSA did not significantly predict PTSD diagnostic status or PTSD severity. MSA was associated with MDD symptom severity *(β = .17, p < .05)* and MDD diagnostic status *(*AOR *= 1.30, p < .001, 95%* CI *[1.08–1.58]).* The logistic regression model with MDD diagnostic status as the outcome variable was significant (*χ^2^ = 34.68, p < .001)* and explained ~4.0% of the variance.
(Kim et al., 2017)	Psychological distress	Age, education level, marital status, dependent children, military type, branch, rank, service classification, length of service, workplace, number of working hours per week, stress from an immediate superior, physical activity, and weight fluctuation	MSH was associated with significantly higher psychological distress *(β = 3.486, p = 0.0397).*
(Kimerling et al., 2007)	Adjustment disorders, anxiety disorders, PTSD, impulse-control disorders, dissociative disorders, eating disorders, psychogenic disorders, somatoform disorders, bipolar disorders, depressive disorders, schizophrenia and psychoses, alcohol disorders, drug use, suicide, and intentional self-inflicted injury	Adjusted odds ratios controlled for age and race	MST was significantly associated with: PTSD *(*OR *= 11.82, 99%* CI *[11.18, 12.50], p < .01;* AOR *= 8.83, 99%* CI *[8.34, 9.35], p < .01)* Impulse-control disorders *(*OR *= 3.40 99%* CI *[2.39, 4.84], p < .01)* Dissociative disorders *(*OR *= 7.47 99%* CI *[5.29, 10.54], p < .01;* AOR *= 4.99, 99%* CI *[3.50, 7.07], p < .01)* Eating disorders *(*OR *= 4.13 99%* CI *[3.30, 5.15], p < .01;* AOR *= 3.05 99%* CI *[2.43, 3.83], p < .01)* Bipolar disorders *(3.12, 99%* CI *[2.92, 3.33], p < .01;* AOR *= 2.25, 99%* CI *[2.10, 2.41], p < .01)* Personality disorders *(4.60, 99%* CI *[4.21, 5.01], p < .01;* AOR *= 3.11, 99%* CI *[2.84, 3.40], p < .01)* Alcohol disorders *(OR = 3.28, 99%* CI *[3.03, 3.55], p < .01; AOR = 2.33, 99%* CI *[2.15, 2.53], p < .01).* MST was significantly associated with Anxiety disorders *(*OR *= 2.20, 99%* CI *[2.10, 2.32], p < .01)*, drug use *(*OR *= 2.97, 99%* CI *[2.73, 3.23], p < .01)*, and suicide and intentional self-inflicted injury *(*OR *= 2.96, 99%* CI *[2.01, 4.37], p < .01)*, but not when adjusting for age and race
(Kimerling et al., 2010)	PTSD, depressive disorders, other anxiety disorders, alcohol and substance use disorders, adjustment disorders	ORs adjusted for age, race/ethnicity, marital status, health insurance, service connection > than 50%, VHA services use before OEF/OIF, > 12 months in VHA care, component, branch, multiple deployments, and recent deployment >6 months’ duration	MST increased likelihood of: any mental health condition *(*OR *= 3.57, 95%* CI *[3.25, 3.92]*, AOR *= 3.28, 95%* CI *[2.97, 3.62])* Depressive disorders *(*OR *= 2.96, 95%* CI *[2.72, 3.22]*, AOR *= 2.64, 95%*CI *[2.41, 2.88])* PTSD *(*OR *= 3.82, 95%* CI *[3.51, 4.16]*, AOR *= 3.83, 95%* CI *[3.49, 4.21])* other anxiety disorders *(*OR *= 2.05, 95%* CI *[1.87, 2.26]*, AOR *= 1.80, 95%* CI *[1.64, 1.99])* alcohol and substance use disorders *(*OR *= 2.89, 95%* CI *[2.53, 3.29]*, AOR *= 1.93, 95%* CI *[1.62, 2.30])* adjustment disorders *(*OR *= 1.68, 95%* CI *[1.51, 1.86]*, AOR *= 1.68 95%* CI *[1.50, 1.87])*
(Laws et al., 2016)	PTSD symptoms in the past 30 days	Combat exposure, age and minority status	There was a significant indirect effect of MST on PTSS via unit relationship quality *(B = 1.08*, SE *= .38, p = .004).*
(Lindsay et al., 2016)	Depressive disorder, bipolar disorder, anxiety disorder, schizophrenia, PTSD, AUD, DUD, personality disorder	Race/ethnicity and age	MST predicted increased risk of depressive disorder (OR *= 3.33, 95%* CI *[1.12, 9.93], p < .05*), bipolar disorder (OR *= 2.87, 95%* CI *[1.12, 7.44], p < .05)*, PTSD *(*OR *= 2.42, 95%* CI *[1.11, 5.24], p < .05)* and personality disorder *(*OR *= 4.61, 95%* CI *[2.02, 10.52], p < .001).* The relationships with anxiety disorder, schizophrenia, AUD, and DUD were not significant.
(Luterek et al., 2011)	PTSD, and DESNOS/CPTSD	Regressions controlled for childhood and other adulthood interpersonal trauma experiences	MSA explained could uniquely 5.9% of the variance in PTSD symptoms and 6.6% in DESNOS symptoms.
(Maguen et al., 2012)	PTSD, depression, anxiety, adjustment disorders, alcohol use disorders, substance use disorders, eating disorders	ORs are adjusted for demographic and military service characteristics	MST predicted increased likelihood of PTSD *(*OR *= 4.17, p < .001, 95%* CI *[3.82–4.56]).* Amongst participants with PTSD, MST was associated with comorbidity of depression (aOR *= 1.54, p < .001, 95%* CI*[1.34–1.77]*), anxiety (aOR *= 1.30, p < .001, 95%* CI*[1.14–1.47])*, AUD (a*OR = 1.39, p < .001, 95%* CI*[1.16–1.65]*), eating disorders (aOR *= 2.61, p < .001, 95%* CI*[1.76–3.88])*, and having 3 or more comorbid mental health diagnoses *(*aOR *= 1.48, p < .001, 95%* CI*[1.31–1.67]).*
(Mahoney et al., 2024)	PTSD	Childhood physical and sexual abuse, age, and minority status	MST (at Timepoint 1 [T1]) was directly associated with intrusion *(β = 3.25*, SE *= .76, p < .001; β = .30*, SE *= .07, p < .001)*, avoidance (*β = 1.59*, SE *= .34, p < .001; β = .31*, SE *= .06, p < .001)*, cognitive/mood (*β = 3.64*, SE *= .78, p < .001; β = .32*, SE *= .07, p < .001)*, and hyperarousal *(β = 3.35*, SE *= .76, p < .001; β = .31*, SE *= .07, p < .001)* symptoms (at Timepoint 2 [T2]). Past-year IPV experiences significantly mediated the association between MST and avoidance symptoms (*β = .20, 95%* CI *[.05, .43], p < .05; β = .08, 95%* CI *[.03, .10], p < .05*) and negative cognitions/mood symptoms (*β = .46, 95%* CI *[.04, .97], p < .05; β = .07*, CI *[.02, .10], p < .05)*, but not intrusions or hyperarousal.
(Mercado et al., 2015)	Overall mental health status, past week depressive symptoms, past month PTSD symptoms	Age	MST was associated significantly with overall mental health status (*F (2, 3215) = 9.60, <.0001)*, depressive symptoms *(F (2, 4255) = 12.27, p < .0001))*, PTSD symptoms (*F (2,11,618) = 21.58, P < .0001).* Compared to women with no MST, women who experienced non-IPV-MST had significantly lower overall mental health (*M = 41.7, 95%* CI *[39.5–43.9] vs M = 48.2, 95%* CI *[46.2–50.2]), p < .001*,), higher depressive symptoms *(M = 21.9, 95%* CI *[19.8–24.1] vs M = 14.5, 95%* CI *[12.6–16.5], p < .001)*, and PTSD symptoms *(M = 42.0, 95%* CI *[39.2–44.8] vs M = 29.7 95%* CI *[27.2–32.3], p < .001).* The mental health status and level of depressive symptoms of women who experienced IPV-MST did not differ significantly from those of women who did not experience MST or those who experienced non-IPV-MST. Women who had experienced IPV- MST had significantly higher levels of PTSD symptoms compared to women with no MST history *(M* = *41.7 vs. 29.7, p < .004).* No significant differences in PTSD symptoms between women who had experienced IPV-MST and women who had experienced non-IPV-MST.
(Monteith et al., 2018)	Suicidal ideation and behavior in the past 3 months	Age, PTSD symptom severity, lifetime suicide attempt history and possible depression	Deployment sexual trauma did not have a significant direct effect on suicidal ideation. Perceived post-deployment support mediated the relationship between deployment sexual trauma and suicidal ideation *(−.01; p < .05).* Perceived unit support did not significantly mediate the relationship between deployment sexual trauma and suicidal ideation.
(Monteith et al., 2023)	Past month and post-military suicidal ideation, post-military suicide attempt	Sensitivity models were adjusted for demographic and military characteristics, time since separation from the military), PTSD, depression, problematic alcohol use in past year)	Crude models: MST predicted post-military suicidal ideation (PR *= 3.21, 95%* CI *[1.79, 5.73], p < .0001).* MSH (PR *= 2.10, 95%* CI *[1.06, 4.14], p = .033)* and MSA (PR*- = 3.80, 95%* CI *[2.12, 6.83], p < .0001)* examined separately predicted post-military suicidal ideation. MST *(*PR *= 2.97, 95%* CI *[1.20, 7.36], p = .018)* predicted suicide attempt. Examined separately, MSA (PR*- = 3.87, 95%* CI *[1.55, 69.62], p = .004*), but not MSH, predicted suicide attempt. MST predicted past-month suicidal ideation *(*PR *= 4.05, 95%* CI *[1.26, 13.01], p = .019*). Examined separately, MSA (PR *= 4.86, 95%* CI *[1.49, 15.77, p = .009)*, (but not MSH) predicted past-month suicidal ideation. Sensitivity models*:* MST predicted post-military suicidal ideation *(*PR *= 2.60, 95%* CI *[1.46, 4.64], p = .001).* Examined separately, both MSH *(*PR *= 2.01, 95%* CI *[1.46, 4.64], p = .001)* and MSA *(*PR*– 2.98, 95%* CI *[1.64, 5.42], p < .001)* predicted post-military suicidal ideation but not suicide attempt. None of the sensitivity models significantly predicted past-month suicidal ideation.
(Moreau et al., 2022)	Depression symptoms, PTSD symptom severity	Age, cohabitation status, education, financial situation, sexual orientation, army rank, military branch, deployment in the last 12 months, military branch, low acceptance of increasing female representation in the army, low social cohesion, higher female representation.	Experiencing MST involving several sexual stressors was associated with increased risk of depressive symptoms (aOR *= 6.5, 95%* CI *[2.6, 16.0], p < .001)*, subthreshold PTSD screening score *(*aRRR *= 3.0, 95%* CI *[2.6, 16.0], p < .001*), and positive PTSD screening score *(*aRRR *= 11.3, 95%* CI *[2.3, 55.6], p < .01*). Experiencing MST involving one form of sexual oppression was associated with depressive symptoms *(*aOR *= 5.2, 95%* CI *[1.9, 13.9], p < .001)*, subthreshold PTSD screening score *(*aRR *= 3.9, 95%* CI *[1.6, 96], p < .01)*, but not significantly associated with a positive PTSD screening score. Repeated sexual comments alone were associated with increased risk of depressive symptoms *(*aOR *= 3.1, 95%* CI *[1.7,5.5], p < .001)* and subthreshold PTSD screening score *(*aRR *= 4.5, 95%* CI *[2.89, 7.4], p < .001)*, but not significantly associated with positive PTSD score. Any sexual assault was associated with increased risk of depressive symptoms *(*aOR *= 2.6, 95%* CI *[1.1, 6.0], p < .05)* but not significantly associated with subthreshold PTSD or positive PTSD score. Sexual harassment was associated with increased risk of depressive symptoms *(*aOR *= 3.7, 95%* CI *[2.5, 5.5], p < .01)* and subthreshold PTSD screening score *(*aRRR *= 3.5, 95%* CI *[2.3, 5.3], p < .01).*
(Murdoch et al., 2006)	Current PTSD symptom severity	History of in-service sexual assault, combat exposure, adult exposures to serious post service trauma and hardships, age, race, education, marital status, employment status, social adjustment	In-service sexual harassment severity was associated with higher PTSD symptom severity (*adjusted r^2^ = 0.238, p < .0001)* and could explain 3.2% of additional variance in PTSD symptom severity.
(Murdoch et al., 2010)	Psychiatric symptoms	The full model adjusted for high magnitude stressors (childhood maltreatment, combat exposure, other high magnitude stressors since entering the military), sociodemographic variables (age, race/ethnicity, gender, education, married status, rank, and years in the military)	Military sexual stressor category was significantly associated with psychiatric symptoms (PTSD, depression, anxiety, and somatic symptoms modeled as a single latent variable) in the reduced model (*B^reduced^ = 0.20, p < .01*) but not the full model.
(Murray-Swank et al., 2018)	PTSD, psychological distress, somatisation, depression, anxiety, suicidality	N/A	Women who reported MST reported significantly higher levels of psychological distress *(t = −3.37, p < .01)*, somatisation *(t = −3.08, p < .01)*, depression *(t = −2.35, p < .05)*, anxiety (*t* = −3.04, *p* < .01), more symptoms of PTSD (*t* = −3.61, *p* < .001), and suicidality (*t* = −3.45, *p* < .001) than those without MST.
(Newins et al., 2021)	PTSD symptom severity, depression symptoms, past week suicidal ideation, alcohol misuse, drug use in last 12 months	N/A	Group differences were observed on all four outcomes (PTSD, depression, alcohol use, and drug use) *6.54, ps = .038).* Women with experiences of MSA had higher scores than those with no history of adult sexual assault group on all four outcomes (*zs − 2.55, ps .011).* MSA was associated with higher depression and PTSD symptoms than Civilian Adulthood Sexual Assault (*zs ≤ 2.78, ps ≤ .005).* Women who experienced MSA were more likely to report clinically significant suicidal ideation compared to women who did not report experiences of sexual assault *(χ ^2^ (1) = 5.90, p = .015)*, but did not differ significantly from women who experienced Civilian Adulthood Sexual Assault.
(Rønning et al., 2024)	Past-week PTSD and current anxiety and depression symptoms	Age, having a spouse/intimate partner, total international deployment time, rank, time since deployment, war zone exposure, childhood sexual harassment, and bullying	Sexual harassment predicted PTSD *(B = 4.77*, SE *= 1.18, p = .000*), anxiety (*B = 1.49*, SE *= 0.44, p = .001)*, and depression (*B = 1.23*, SE *= 0.43, p = .004)* symptom severity.
(Sandhu et al., 2022)	Disordered eating	N/A	MSA did not directly significantly predict total disordered eating and did not indirectly predict disordered eating via PTSD symptoms. There was no significant direct effect of MSA on dieting or indirect effect via PTSD on dieting. There was no significant direct effect of MSA on oral control or indirect effect via PTSD. MSA uniquely predicted PTSD symptoms (*path a; β = 14.04, 95%* CI *[7.80, 20.29], p < .0001)* and PTSD symptoms uniquely predicted bulimia and food preoccupation *(path b; β = .05, 95%* CI *[.02, .09], p = .01).* MSA predicted higher symptoms of bulimia and food preoccupation *(β = 1.44, 95%* CI *[.28, 2.60], p = .02).* PTSD fully significantly mediated the relationship between MSA and bulimia and food preoccupation.
(Skinner et al., 2000)	Depression, general mental health	Age	Compared to no sexual harassment *(M = 65.3*), sexual harassment (*M = 59.5*) significantly associated with lower mental health *(t = 53.1, p < .0001*) and depression (*X ^2^ = 100.3, p < .001*). Compared to no sexual assault (*M = 64.6*), sexual assault (*M = 53.7)* was significantly associated with lower mental health *(t = 144.0, p < .0001)* and depression (*X ^2^ = 200.9, p < .001*).
(Smith et al., 2017)	Current PTSD symptom severity	N/A	Sexual harassment was directly associated with PTSD symptoms (*r = .28, p < .05)* and indirectly via social reintegration difficulty (*β = .09, p = .002).*
(Smith et al., 2020)	PTSD	N/A	MSA at Timepoint 1 (T1) was associated with PTSD symptoms at T1 (*β = 0.84*, SE *= 0.33, p = .011)*, Timepoint 2 (T2) *(β = 1.05*, SE *= 0.34, p = .002*), and Timepoint 3 (T3) *(β = 1.11*, SE *= 0.43, p = .009).* MSH did not significantly predict PTSD symptoms. Coworker support significantly moderated the association between MSA and PTSD symptoms at T2 *(β = − 1.34*, SE *= 0.39, p = .001*) and T3 *(β = − 0.70*, SE *= 0.33, p = .032)*, but not at T1.
(Stefanovics et al., 2023)	Past year suicidal ideation	N/A	MST predicted increased risk of past year suicidal ideation *(*OR *= 4.32, 95%* CI *[2.16, 8.63], p < .0001).*
(Street et al., 2008)	Current symptoms of depression, current (past month) and lifetime PTSD, and past 6-month somatic symptoms	Age, race and reserve component status	MSH (not including MSA) predicted probable depression *(*aOR *= 1.75; 95%* CI *= [1.37–2.24])* and somatic symptoms *(*aOR *= 1.77, 95%* CI *= [1.48, 2.12]).* Reporting both MSH and MSA predicted increased risk of depression *(*aOR *= 4.51, 95%* CI *[3.30–6.16])* and somatic symptoms *(*aOR *= 2.83, 95%* CI *= [2.74, 4.87]).* Experiencing both MSH and MSA was associated with greater risk of current PTSD (aOR *= 7.15, 95%* CI *[4.03, 12.69])* and lifetime PTSD (aOR *= 7.03, 95%* CI *[5.05, 9.79])* than MSH only.
(Sumner et al., 2021)	PTSD, depression, bipolar disorder, anxiety, suicidal ideation, alcohol dependence, substance dependence	ORs adjusted for age, race, branch of service, and years enrolled in the VHA	MST increased odds of PTSD *(*OR *= 5.41, 99%* CI *[5.28–5.56]*), depression *(*OR *= 2.35, 99%* CI *[2.28–2.40])*, bipolar disorder *(*OR *= 2.51, 99%* CI *[2.39–2.64])*, anxiety *(*OR *= 1.85, 99%* CI *[1.79–1.88])*, suicidal ideation *(*OR *= 2.98, 99%* CI *[2.89–3.05])*, alcohol dependence *(*OR *= 2.74, 99%* CI *[2.66–2.82])*, substance dependence (OR *= 2.59, 99%* CI *[2.45–2.72]).*
(Surís et al., 2004)	PTSD	Age, ethnicity (African American versus other), education (in years), marital status (divorced/never married versus other), and employment status, childhood sexual assault, civilian adulthood sexual assault	MSA significantly increased risk of PTSD *(*OR *= 9.27, 95%* CI *= [3.75–22.95], Wald [1] = 23.18, p < .0001*). This association remained significant after adjusting for covariates *(*aOR *= 3.87, 95%* CI *= [2.09–7.17]*, Wald *[1] = 18.5, p < .0001).* MSA significantly increased risk of PTSD when compared with civilian sexual assault *(*aOR *= 3.26, 95%* CI *= [1.04–10.20]*, Wald *[1] = 4.11, p < .05)*, without childhood sexual assault (i.e. civilian sexual assault only) *(*aOR *= 5.06, 95%* CI *= [2.34–10.93]*, Wald *[1] = 17.03, p < .0001*), civilian sexual assault (with or without childhood sexual assault) (aOR *= 4.15, 95%* CI *= [1.72–10.01]*, Wald *[1] = 9.98, p < .01).* Participants who had experienced MSA (and any other sexual assault) had higher risk of PTSD (aOR *= 3.88, 95%* CI *= [1.64–9.23]*, Wald *= 9.47, p < .005).*
(Surís et al., 2007)	Psychological symptom patterns, depression, harmful alcohol use, general mental health	Marital status, age, years of education, ethnicity, and employment status	MSA was associated with significantly higher depression symptoms *(M = 9.9*, SD *= 3.2)* than civilian sexual assault *(M = 8.9, SD = 3.1)*, and no assault groups (*M = 8.1*, SD *= 3.3); (p < .01).* Mean harmful alcohol use scores were not significantly different between groups, but MSA group had marginally higher harmful alcohol use scores and higher screening prevalence for harmful alcohol compared to those with no sexual assault histories *(14% vs. 5.5%).* MSA was associated with significantly higher global severity of psychiatric symptoms, positive symptom distress, hostility and phobic anxiety than civilian sexual assault.
(Webermann et al., 2023)	Past month PTSD symptom severity	Combat trauma, childhood abuse, age, race.	Unit support and interpersonal support mediated the relationship between MST and PTSD symptoms. MST was negatively associated with Time 1 unit support (*β = −.23, 95%* CI *[−0.33, −0.13], p < .001)*, which was negatively associated with Time 2 PTSD symptoms *(β = −.13, [−0.24, −0.03], p = .013).* MST was negatively associated with T1 interpersonal support *(β = −.16, [−0.27, −0.06], p = .002*), which was negatively associated with Time 2 PTSD symptoms (*β = −.25, [−0.35, −0.15], p < .001).* When controlling for both unit and interpersonal support, in addition to the model covariates, MST was directly associated with PTSD *(β = .14, [0.04, 0.24], p = .004).*
(Wilson et al., 2020)	Self-directed violence	Childhood sexual abuse, combat trauma, PTSD, MDD	MSA was associated with a significant increase in risk for attempting suicide *(*aOR *= 3.599, p = .002).*
(Wolfe et al., 1998)	PTSD symptomology	Combat exposure, leadership support, unit cohesion, coping style, intervening life events	Experiencing sexual assault was associated with higher mean PTSD symptomology *(M = 91.83*, SD *= 22.69*) compared to no sexual harassment/ assault experiences (*M = 71.35*, SD *= 17.35)*, physical sexual harassment *(M = 77.80*, SD *= 24.21)* and verbal sexual harassment *(M = 73.39*, SD *= 16.17).* More frequent physical sexual harassment was associated with significantly increased PTSD symptomatology 18–24 months post-deployment *(B = 2.43*, SE *= 1.22, β = .16, p < .05)*, but lost significance after controlling for intervening life events. The relationship between physical sexual harassment and PTSD symptomology was mediated by intervening life events (*β = .332, p < .001).*
(Yaeger et al., 2006)	Current PTSD	ORs adjusted for age at time of study, age at time of entry to service, education, work status, marital status, years of active-duty service, race, branch of service	MST predicted risk of PTSD *(*OR *= 4.4, 95%* CI *[2.4 to 8.2]).*
(Yalch et al., 2018)	Hazardous alcohol use, hazardous drug use, PTSS	N/A	MSA significantly predicted increase in drug use *(β = 0.12, p < .05, 95%* CI *[0.02, 0.22])*, and PTSS (*β = .36, p < .05, 95%* CI *[0.27, 0.44]).* MSA predicted risk of drug use *(*OR *= 1.91, p < .05, 95%* CI *[1.01, 3.62])* and PTSS (OR *= 4.96, p < .05, 95%*CI *[3.05, 8.08]).* Participants in the low trauma exposure group were more likely to not meet diagnostic levels of comorbid SUD and PTSS and less likely to meet diagnostic levels of SUD symptoms. Participants in the high trauma exposure group were more likely to exhibit probable SUD and comorbid SUD and PTSS, and less likely to exhibit no diagnostic-level symptoms. Participants in the MSA only group were less likely to exhibit probable SUD.
(Zelkowitz et al., 2022)	Disordered eating symptoms, PTSD	Age and Body Mass Index	MST was significantly directly associated with both PTSD (*β = 0.16*, SE *= 0.02, p < .05, 95%* CI *[0.12, 0.19]* and shape/weight concerns (*β = 0.21*, SE *= 0.03, p < .05, 95%* CI *[0.15, 0.27]*, but not with disordered eating. MST was significantly associated with disordered eating indirectly via shape/ weight concerns *(β = 0.06*, SE *= 0.01, p < .05, 95%* CI *[0.04, 0.08]).* There was not a significant indirect association between MST and disordered eating symptoms via PTSD symptoms.
(Zerach, 2023)	PTSD and CPTSD	Combat experiences, ACEs and potentially morally injurious events	MSH was associated with higher levels of PTSD symptoms *(M = 8.84*, SD *= 6.54)* than no MST (*M = 6.61*, SD *= 6.43); F (1, 579) = 14.63, p = .00, ηp2 = .05).* MSA was associated with higher levels of PTSD symptoms (*M = 11.90*, SD *= 7.00*), as compared to those not reporting any MST *(M = 6.83*, SD *= 6.32); F (1, 579) = 30.64, p, .00, ηp2 = .05).* MSA (*M = 9.65*, SD *= 6.43)* was associated with higher CPTSD symptoms than no MSA *(M = 7.40*, SD *= 6.07); F (1, 579) = 4.16, p, .05, ηp2 = .01.).* MSA was associated with higher levels of PTSD symptoms *(B = 1.53, p, .05)* (but MSH was not) and was not associated with increases in CPTSD symptoms.

*Note:* ACE, adverse childhood experience; AUD, alcohol use disorder; aOR, adjusted odds ratio; aRR, adjusted risk ratio; CI, confidence interval; CMD, common mental disorders; CPTSD, complex posttraumatic stress disorder; DESNOS, disorders of extreme stress not otherwise specified; DUD, drug use disorder; IPV, intimate partner violence; IPV-MST, intimate partner violence- related military sexual trauma; M, mean, MDD, major depressive disorder; MSA, military sexual assault, MSH, military sexual harassment; MST, military sexual trauma; MSV, military sexual violence; N/A, not applicable; Non-IPV-MST, non-intimate partner violence-related MST; OEF, operation enduring freedom; OIF, Operation Iraqi Freedom; OR, odds ratio, PR, prevalence ratio; PTSD, posttraumatic stress disorder; PTSS, posttraumatic stress symptoms; RR, risk ratio; SD, standard deviation; SE, standard error: SI, suicidal ideation, SUD, substance use disorder; VHA, Veterans Health Administration.

#### PTSD outcomes (n = 33)

Studies examining MST dichotomously (as an umbrella term for a range of behaviours) found associations with probable PTSD (Blais, Livingston, Barrett, & Tannahill, 2023; Fontana & Rosenheck, 1998; Gibson et al., 2019; Himmelfarb, Yaeger, & Mintz, 2006; Kimerling et al., 2010; Kimerling, Gima, Smith, Street, & Frayne, 2007; Lindsay et al., 2016; Maguen et al., 2012; Yaeger, Himmelfarb, Cammack, & Mintz, 2006; Zelkowitz, Sienkiewicz, Vogt, Smith, & Mitchell, 2022) and higher PTSD symptom scores (Banducci, McCaughey, Gradus, & Street, 2019; Blais et al., 2023; Cobb Scott et al., 2014; Decker et al., 2021; Dutra et al., 2011; Esopenko et al., 2023; Luterek, Bittinger, & Simpson, 2011; Mercado, Ming Foynes, Carpenter, & Iverson, 2015; Murdoch et al., 2006; Murray-Swank, Dausch, & Ehrnstrom, 2018; Rønning et al., 2024; Wolfe et al., 1998) across intrusion, avoidance, cognitive/mood, and hyperarousal symptom clusters (Mahoney, Shayani, & Iverson, 2024).

When MST subtypes were examined, MSA more consistently predicted greater PTSD severity (Gorman et al., 2021; Gross et al., 2018; Luterek et al., 2011; Wolfe et al., 1998; Zerach, 2023) and probable PTSD (Street, Stafford, Mahan, & Hendricks, 2008) than MSH (Blais, Brignone, Fargo, Livingston, & Andresen, 2019; Kang, Dalager, Mahan, & Ishii, 2005; Street et al., 2008; Surís, Lind, Kashner, Borman, & Petty, 2004), though MSH was linked to probable PTSD in two studies (Hendrikx, Williamson, & Murphy, 2023; Kang et al., 2005). In a study stratified by sexual orientation, MSA increased PTSD symptom severity in the heterosexual sample (Gorman et al., 2021), with the small sexual minority sub-sample perhaps lacking statistical power to detect an association. Four studies reported no association between MSA and probable PTSD (Gorman et al., 2021; Hendrikx et al., 2023; Kearns et al., 2016; Moreau et al., 2022) and severity (Kearns et al., 2016).

Interpersonal support appeared to influence these associations. During deployment, reduced interpersonal support from military networks (Laws, Mazure, McKee, Park, & Hoff, 2016; Webermann et al., 2023) mediated links with PTSD symptoms, and concerns surrounding relationships at home exacerbated the impact of MST on PTSD severity (Banducci et al., 2019). Post-deployment, detriments to non-military networks mediated the association between MST and PTSD symptoms (Fontana & Rosenheck, 1998; Smith, Brady, Hammer, Carlson, & Mohr, 2020; Smith, Wang, Vaughn-Coaxum, Di Leone, & Vogt, 2017; Webermann et al., 2023), and intimate partner violence (IPV) experiences mediated the association between MST and PTSD-avoidance and PTSD-negative cognitions/mood symptoms (Mahoney et al., 2024).

Compared to other traumas, MST predicted higher risk of PTSD (Blais et al., 2023) and symptom severity (Surís et al., 2004) than civilian sexual assault. Comparisons of perpetrator identity (MST perpetrated by intimate partners versus perpetrators who were not intimate partners) revealed no significant differences (Mercado et al., 2015), suggesting perpetrator identity cannot explain these differences. Considered alongside other trauma exposures, MST and IPV cumulatively predicted higher PTSD symptoms (Esopenko et al., 2023). Although one study did not find an interaction between MSA and combat exposure in PTSD symptom severity (Gross et al., 2018), another study found MST interacted with combat exposure to increase combat-related PTSD symptoms (Cobb Scott et al., 2014), suggesting MST may exacerbate existing vulnerabilities.

This review found limited evidence linking MST and Complex PTSD (CPTSD) (Luterek et al., 2011; Zerach, 2023). CPTSD, characterized by PTSD symptoms plus affective dysregulation, negative self-concept and relationship disturbances, often arises from prolonged or repeated trauma exposure (Herman, 1992) and may be influenced by additional factors.

#### Depression outcomes (n = 23)

Most studies reported associations with MST and probable depression (Gibson et al., 2019; Gorman et al., 2021; Gradus, Street, Kelly, & Stafford, 2008; Kearns et al., 2016; Kimerling et al., 2010; Maguen et al., 2012; Moreau et al., 2022; Rønning et al., 2024) and higher symptom severity (Blais et al., 2023; Gorman et al., 2021; Kearns et al., 2016; Murray-Swank et al., 2018). Null findings from a small, high-risk-of-bias study in US serving women (Dutra et al., 2011) and in study with sexual minority US ex-servicewomen (Gorman et al., 2021) may have reflected low statistical power.

When MST sub-types were examined separately, MSA predicted probable depression (Blais et al., 2019; Gibson et al., 2019; Gross, Kroll-Desrosiers, & Mattocks, 2020; Hankin et al., 1999; Skinner et al., 2000; Street et al., 2008) and higher depression severity more consistently than MSH (Blais et al., 2019). Religious service attendance buffered the impacts of MSA on depression in one study with US ex-servicewomen (Chang, Skinner, & Boehmer, 2001).

Compared with interpersonal trauma experienced outside of military service, MST was associated with higher depression severity (Newins et al., 2021) but not higher risk of probable depression (Blais et al., 2023). Comparisons of perpetrator identities (intimate partners versus not intimate partners) revealed no significant differences in depressive symptoms (Mercado et al., 2015). Two studies suggested that cumulative exposures (e.g. non-military sexual trauma) (Blais et al., 2023) and IPV (Esopenko et al., 2023) alongside MST may elevate depression risk (Blais et al., 2023) and symptom severity (Esopenko et al., 2023).

#### Anxiety outcomes (n = 8)

Five studies reported significant associations between MST and probable anxiety (Gibson et al., 2019; Kimerling et al., 2010; Maguen et al., 2012; Murray-Swank et al., 2018; Sumner et al., 2021) and one with higher anxiety symptom severity (Rønning et al., 2024). One study found that this relationship lost significance when adjusting for age and race (Kimerling et al., 2007) and another found no association in a sample of US transgender ex-servicewomen (Lindsay et al., 2016), highlighting the potential influence of demographic factors.

#### Suicidality outcomes (n = 17)

Most studies reported associations between MST and suicidality (Murray-Swank et al., 2018), including suicidal behaviours (Wilson et al., 2020) and ideation (Blais et al., 2019; Blais et al., 2023; Blais & Geiser, 2019; Decker et al., 2021; Esopenko et al., 2023; Gibson et al., 2019; Gradus, King, Galatzer-Levy, & Street, 2017; Gross et al., 2020; Hoffmire et al., 2021; Stefanovics, Potenza, Tsai, Nichter, & Pietrzak, 2023). Both MSH and MSA increased risk of suicidal ideation (Gross et al., 2020; Monteith et al., 2023), with MSA more consistently reported as a predictor (Blais et al., 2019; Monteith et al., 2023). One study identified depressive symptom severity and PTSD-related anhedonia as mediators in the association between MSA and suicidal ideation. No significant pathways were observed for MSH (Blais & Geiser, 2019), indicating potentially distinct mechanisms.

Comparisons of MST with other interpersonal traumas did not find unique effects of MST. MSA was associated with suicidal ideation in US serving and ex-servicewomen, but this association did not differ significantly from civilian adulthood sexual assault (Newins et al., 2021). Similarly, suicidal ideation and behaviour did not differ among US ex-servicewomen who had experienced MST only, IPV only, or both MST and IPV (Esopenko et al., 2023).

One study with university-enrolled US serving and ex-servicewomen did not identify an association between MST and suicidality (Bryan, Bryan, & Clemans, 2015), perhaps reflecting potentially distinct support resources available to participants in this sample. Accordingly, another study found that perceived post-deployment support mediated the relationship between deployment MST and suicidal ideation (Monteith et al., 2018). Together, findings implicate psychological distress and access to social support as influential in the relationship between MST and suicidality.

#### Harmful substance use outcomes (n = 11)

Most studies observed associations between MST and probable harmful substance use (Gibson et al., 2019; Hankin et al., 1999; Kimerling et al., 2010; Maguen et al., 2012; Surís, Lind, Kashner, & Borman, 2007; Yalch, Hebenstreit, & Maguen, 2018), as well as greater symptom severity (Yalch et al., 2018). Two studies identified PTSD (Banducci et al., 2019) and depression (Gradus et al., 2008) symptoms as potential mediators of this relationship. One small study did not find an association between MST and past year alcohol problems in US serving women (Fillo, Goodell, Homish, & Homish, 2023), perhaps reflecting limited statistical power.

Studies found MSA was more consistently associated with MSA than MSH. Specifically, MSA (but not MSH) predicted harmful alcohol use in UK ex-servicewomen (Hendrikx et al., 2023) and was associated with both higher harmful alcohol use scores and risk for harmful alcohol use in US ex-servicewomen (Surís et al., 2007). Despite positive associations between higher MST frequency and alcohol use severity (Banducci et al., 2019), one study found high exposure to any military stressors increased probable substance use disorder (SUD) risk, whilst exposure to MSA alone was associated with higher SUD symptoms but not greater SUD risk (Yalch et al., 2018). Taken together, findings suggest that trauma exposure generally, rather than MST itself, may influence substance use.

#### Disordered eating outcomes (n = 5)

Findings related to MST and disordered eating were mixed. Two studies found that MST was associated with probable eating disorders in US ex-servicewomen (Breland, Donalson, Dinh, & Maguen, 2018) and with a comorbid eating disorder amongst those with PTSD (Maguen et al., 2012). The role of PTSD in this association remained unclear. One high-risk-of-bias study found PTSD mediated the relationship between MSA with bulimia nervosa and food preoccupation symptoms in US ex-servicewomen (Sandhu, Dougherty, & Haedt-Matt, 2022). Contrastingly, another study did not find a mediating role of PTSD, but suggested an indirect pathway via shape and weight concerns (Zelkowitz et al., 2022). In US serving women, psychological distress fully mediated the relationship between MST and disordered eating symptoms (Harned & Fitzgerald, 2002), with the focus on recent workplace MST potentially reflecting a unique context where participants may have remained in contact with perpetrators.

#### General mental health and functioning outcomes (n = 10)

Findings indicated that MST negatively impacted general mental health in both serving (Harned, Ormerod, Palmieri, Collinsworth, & Reed, 2002; Kim, Lee, Lee, Han, & Park, 2017) and ex-servicewomen (Mercado et al., 2015; Skinner et al., 2000), with one study observing greater negative impacts of MST than non-military sexual assault (Surís et al., 2007). Religious service attendance was identified as a potential protective factor (Chang et al., 2001). With regards to somatic symptoms specifically, a positive association with MST was observed in US ex-servicewomen (Murray-Swank et al., 2018), with MSH and MSA each identified as predictors (Street et al., 2008). Contrastingly, only MSH was associated with high physical somatisation in UK ex-servicewomen (Hendrikx et al., 2023).

##### **Combined outcomes (**n = 2)

Neither study examining mental health as a combined variable observed significant associations with either MSH or MSA (Hendrikx et al., 2023) (Murdoch et al., 2010).

#### Other psychiatric conditions (*n* **= 4)**

MST was associated with impulse-control disorders, dissociative disorders, bipolar disorder (Kimerling et al., 2007; Sumner et al., 2021), and personality disorders (Sumner et al., 2021) in US ex-servicewomen. In one study with transgender US ex-servicewomen, MST predicted bipolar disorder and personality disorder but not schizophrenia (Lindsay et al., 2016).

### Qualitative findings

Qualitative synthesis yielded 27 findings (Table 3). One unsupported finding was excluded, leaving 26 unequivocally (*n* = 9) and equivocally supported (*n* = 17) findings, which were grouped into 13 categories and then collated into four synthesized findings (Table 4). Although themes like maladaptive coping and negative changes to self-perception overlapped with mental health impacts, they were analyzed separately due to being interpreted as secondary responses to primary outcomes like PTSD and depressive symptoms.

**Table 3.** Qualitative findings and supporting evidence

Finding(s)	Evidence	Credibility
1) Trauma-related difficulties consistent with PTSD (intrusion) (Reinhardt et al., 2023)	*‘There would be flashbacks but I didn’t get it… little things that would come through my head, like oh my god I can’t believe I went through that. Or “wow that reminds me of something”’* (Reinhardt et al., 2023, p. 728)	Unequivocal
2) Trauma-related difficulties consistent with PTSD (avoidance)(Reinhardt et al., 2023)	*‘I try very hard to block it out because it [memories of the MST] makes me ill’* (Reinhardt et al., 2023, p. 728) *‘I have a tendency to suppress things and convince myself they don’t really bother me’* (Reinhardt et al., 2023, p. 728)	Unequivocal
3) Trauma-related difficulties consistent with PTSD (mood-related difficulties) (Reinhardt et al., 2023)	Authors discussed how participants described *‘experiencing extremes: anger and reckless behaviour, as well as numbness’* and *‘increased feelings of shame and fear, as well as guilt and anger’* (Reinhardt et al., 2023, p. 729)	Equivocal
4) Trauma-related difficulties consistent with PTSD (arousal) (Reinhardt et al., 2023)	*‘I did not realise how angry I was… I didn’t realize I would talk loud and yell at people… it just grew and grew’* (Reinhardt et al., 2023, p. 729)	Equivocal
5) Minimisation/denial to cope with intense emotions post-MSA (Katz et al., 2017)	*‘I didn’t say anything, just tried to deal with it’.* (Katz et al., 2017, p. 187)	Equivocal
6) Internalisation of self-blame linked to shame (Brownstone et al., 2018)	*‘I hate myself… can’t help but think that if I looked different, none of this would have happened. If I was super skinny or didn’t look like I do, nobody would have found me attractive, I wouldn’t have been harassed as much’.* (Brownstone et al., 2018, p. 407) *‘I thought it was my fault for drinking … too immature, too stupid to know that could have happened’* (Brownstone et al., 2018, p. 407) *‘I saw myself as being naive, ignorant … got into situation that I should have known better’* *‘Had to keep it all inside … felt dirty, shamed, I thought it was my fault … I felt like I was this unclean leper, damaged thing’.* (Brownstone et al., 2018, p. 407) *‘I felt shame and embarrassment for being in that situation’.* (Brownstone et al., 2018, p. 407)	Unequivocal
7) Sleep disturbances/nightmares (Katz et al., 2017)	*‘I had nightmares of people crawling through my bedroom window to get me’. ‘I dreamt I had cockroaches crawling in my womb’.* (Katz et al., 2017, p. 187)	Equivocal
8) Paranoia/ fear of reoccurrence (Katz et al., 2017)	*‘I felt really paranoid all of the time’.* (Katz et al., 2017, p. 187) *‘I felt really paranoid around men’* (Katz et al., 2017, p. 187)	Equivocal
9) Mood-related difficulties (Reinhardt et al., 2023)	*‘I was depressed a lot and it got worse after I was raped’* (Reinhardt et al., 2023, p. 729) *‘There’s nothing I do day to day, minute by minute that really makes me happy or makes me glad I’m alive’* (Reinhardt et al., 2023, p. 729) *‘[the depression] just left me by myself. Not having any friends, just sitting in a room with a TV’* (Reinhardt et al., 2023, p. 729)	Unequivocal
10) Fear/ anxiety (Katz et al., 2017)	*‘I felt uncomfortable in my own room’.* (Katz et al., 2017, p. 187)	Equivocal
11) Anxiety-related difficulties (Reinhardt et al., 2023)	*‘I just can’t see me getting any better…I’m still going to stay… with anxiety’* (Reinhardt et al., p730) *‘I go through… a lot of anxiety. I cry a lot, I isolate a lot, and I’m just comfortable with me and my cat in my apartment and that’s where I feel safe’* (Reinhardt et al., 2023, p. 730)	Unequivocal
12) Suicidal ideation/ attempt post-MSA (Katz et al., 2017)	*‘I tried to overdose on prescription medication’.* (Katz et al., 2017, p. 187) *‘I took different kinds of pills—muscle relaxers and sleeping pills, hoping I wouldn’t wake up’* (Katz et al., 2017, p. 187) *‘I wanted to die, I tried to jump off a cliff but a NCO (non-commissioned officer) stopped me’.* (Katz et al., 2017, p. 187) *‘After the military I jumped out of a window, tried to suffocate myself and cut my leg open to find an artery’* (Katz et al., 2017, p. 187)	Equivocal
13) Suicidal behaviour (Reinhardt et al., 2023)	*‘I was asking God to help me because I couldn’t do that anymore, and I lined up all my pills because I was going to take them and then I started praying’* (Reinhardt et al., 2023, p. 730) *‘… and just remembering I don’t care. I can’t be sad anymore. I don’t want to spend another night crying myself to sleep. And the next day I tried to overdose on ecstasy’. (Reinhardt et al., 2023*, p. 730)	Unequivocal
14) Substance use difficulties (Reinhardt et al., 2023)	*‘a lot of stuff I suppressed with drugs and alcohol so that’s why… things don’t really bother me because I was suppressing everything’* (Reinhardt et al., 2023, p. 729) *‘I think my alcoholism was because of what happened to me, because I relied on alcohol to block everything and the more it was coming, the more I was drinking’* (Reinhardt et al., 2023, p. 730) *‘I don’t know if my coping strategies grew from that to be increasingly more getting myself into trouble with substance. That, if that [the MST] hadn’t happened, if I would have truly been in a better way… I don’t know that’* (Reinhardt et al., p. 729)	Equivocal
15) Substance use for numbing (Katz et al., 2017)	‘*I got in trouble 2–3 times for drinking—to numb the events’.* (Katz et al., 2017, p. 187) *‘I got drunk to not think about the event’* (Katz et al., 2017, p. 187)	Equivocal
16) Posttraumatic growth: relating to others (Reinhardt et al., 2023)	*‘it’s okay to let somebody else know that they’re not alone… I find myself sending that message because I was open to let somebody send that message to me’* (Reinhardt et al., 2023, p. 733)	Equivocal
17) Posttraumatic growth: appreciation of life (Reinhardt et al., 2023)	*‘I wouldn’t take away chapter 1…it all flows from the first chapter…it all flows and it’s all part of the end of the book that I haven’t come to yet…I’m more peaceful now because of the chapters in the book and the way they flow together that have made sense of chapter 1 that was pretty drastic and dramatic… I feel more at peace now’* (Reinhardt et al., 2023, p. 733) *‘not all bad either though, cuz it makes me what I am today’* (Reinhardt et al., 2023, p. 733)	Equivocal
18) Posttraumatic growth: personal strength (Reinhardt et al., 2023)	*‘I have to live with these experiences. The impact of it [the MST] affects me to this present day if I allow it to and I choose not to. I knew…that I have to live. I have to take care of me’* (Reinhardt et al., 2023, p. 733) *‘I believe in myself now … I have been through so much, girl … My life has been a roller-coaster, but I can say at this stage of my life that I believe in me’.* (Reinhardt et al., 2023, p. 733) *‘I fight now. I fight it. You’re not going to walk all over me, you’re not [going to] do this behaviour and get away with it’* (Reinhardt et al., 2023, p. 733) *‘And building my strength back and fighting again… I’m the strongest person I’ve ever been’ (Reinhardt et al., 2023*, p. 733)	Equivocal
19) Coping methods for escaping memories/ emotions related to MST (Brownstone et al., 2018)	*‘I put my head in the sand and hoped it would go away’* (Brownstone et al., 2018, p. 407) *‘I definitely numb out more than I should’* (Brownstone et al., 2018, p. 408) *‘I used to use alcohol a lot to suppress thoughts’* (Brownstone et al., 2018, p. 408) *‘I will be more likely to turn toward addictive behaviours, I developed eating disorders after that…’* (Brownstone et al., 2018, p. 408) Other behaviours such as cannabis use, risky sexual behaviours, risky driving and compulsive shopping were also cited by authors as discussed by participants	Unequivocal
20) Health risk behaviours (Reinhardt et al., 2023)	*‘I got into a car accident before I ended up in the psych ward because I was just in a rage’* (Reinhardt et al., 2023, p. 732) *‘I didn’t care who I slept with after that [the MST] … I felt like, doesn’t matter I blew it already’* (Reinhardt et al., 2023, p. 732)	Equivocal
21) Support from others as influential in the recovery process (Brownstone et al., 2018)	‘One participant explained that meeting her husband was a *“turning point,”* after which she began to seek friendships and returned to being a *‘genuinely happy person’.* (Brownstone et al., 2018, p. 408) *‘I would talk to people like my friends, other people that would hear everything going on …they would just tell me ‘yeah, I know’ … I would talk to my husband about it’.* (Brownstone et al., 2018, p. 408)	Equivocal
22) Positive impacts of formal help-seeking post MSA (Katz et al., 2017)	*‘I am more at peace with myself and others’.* (Katz et al., 2017, p. 187) *‘I’m a lot happier in life now’.* (Katz et al., 2017, p. 187) *‘I feel stronger and more focused’.* (Katz et al., 2017, p. 187) *‘I appreciate life more’.* (Katz et al., 2017, p. 187)	Equivocal
23) Interpersonal challenges (Reinhardt et al., 2023)	*‘But it’s a trust issue and that experience [the MST] caused me to not trust. You can’t trust nobody, you gotta trust yourself …’* (Reinhardt et al., 2023, p. 730) *‘… it [the MST] changed me. And it made me feel powerless against everyone’* (Reinhardt et al., 2023, p. 730) *‘After the rape I lost my, I lost everything. My pride… you know’* (Reinhardt et al., 2023, p. 730)	Equivocal
24) Loss of trust (Katz et al., 2017)	*‘Afterwards, it was hard to have relationships’* (Katz et al., 2017, p. 187) *‘I couldn’t trust him’* (Katz et al., 2017, p. 187) *‘didn’t know how to feel love anymore’* (Katz et al., 2017, p. 187) *‘I wouldn’t let people get close to me’* (Katz et al., 2017, p. 187)	Equivocal
25) Lowered self-esteem and self-worth (Brownstone et al., 2018)	Participants described that MST *‘lowered [their] self-esteem’* and ‘*took confidence away from [them]’* (Brownstone et al., 2018, pp. 406–407) *‘I think initially it made me feel like there was no value to anything I said … like my saying no … it didn’t matter … felt as though everything I said or did didn’t matter’* (Brownstone et al., 2018, p. 407)	Unequivocal
26) Body dissatisfaction following MST (Brownstone et al., 2018)	*‘I hate my body now’* (Brownstone et al., 2018, p. 407) *‘very self-conscious about [my body], don’t think I could ever be skinny enough…I don’t like my body. I have a bad body image… my husband can tell me I look beautiful and I think “yeah right.”* (Brownstone et al., 2018, p. 407) *‘Always think I’m either underweight or overweight … still not okay with my image, how I look, how I talk, how I act’.* (Brownstone et al., 2018, p. 407)	Unequivocal
27) Guilt/Shame (Katz et al., 2017)	N/A	Unsupported

*Note:* N/A, not applicable.

**Table 4.** Synthesized findings and categories

Synthesized finding	Category	Finding(s)
(1) Mental health impacts	PTSD symptoms	1) Trauma related difficulties consistent with PTSD (intrusion symptoms) (Reinhardt et al., 2023)
		2) Trauma related difficulties consistent with PTSD (avoidance symptoms) (Reinhardt et al., 2023)
		3) Trauma related difficulties consistent with PTSD (mood-related difficulties) (Reinhardt et al., 2023)
		4) Trauma related difficulties consistent with PTSD (arousal) (Reinhardt et al., 2023)
		5) Minimisation/ denial to deal with intense emotions post-MSA (Katz et al., 2017)
		6) Internalisation of self-blame linked to shame (Brownstone et al., 2018)
		8) Paranoia/ fear of reoccurrence (Katz et al., 2017)
	Negative changes to mood	9) Mood related difficulties (Reinhardt et al., 2023)
		3) Trauma related difficulties consistent with PTSD (mood related difficulties) (Reinhardt et al., 2023)
	Anxiety	10)Fear/ anxiety (Katz et al., 2017)
		11) Anxiety related difficulties (Reinhardt et al., 2023)
	Suicidality	12) Suicidal ideation/ attempt post-MSA (Katz et al., 2017)
		13) Suicidal behaviour (Reinhardt et al., 2023)
	Substance use	14) Substance use difficulties (Reinhardt et al., 2023)
		15) Substance use for numbing (Katz et al., 2017)
	Posttraumatic growth	16) Posttraumatic growth: Relating to others (Reinhardt et al., 2023)
		17) Posttraumatic growth: Appreciation of life (Reinhardt et al., 2023)
		18) Posttraumatic growth: Personal strength (Reinhardt et al., 2023)
(2) Maladaptive coping	Avoidance coping mechanisms	19) Coping methods for escaping memories/ emotions related to MST (Brownstone et al., 2018)
		15) Substance use for numbing (Katz et al., 2017)
		14) Substance use difficulties (Reinhardt et al., 2023)
	Health risk behaviours as coping mechanisms	20) Health risk behaviours (Reinhardt et al., 2023)
		15) Substance use for numbing (Katz et al., 2017)
		14) Substance use difficulties (Reinhardt et al., 2023)
(3) Support from others is important for mental health	Help-seeking/ support	21) Support from others as influential in the recovery process (Brownstone et al., 2018)
		22) Positive impacts of formal help-seeking post MSA (Katz et al., 2017)
	Negative changes to interpersonal connections	23) Interpersonal challenges (Reinhardt et al., 2023)
		24) Loss of trust (Katz et al., 2017)
(4) Negative changes to self-perception	Self-blame	6) Internalisation of self-blame linked to shame (Brownstone et al., 2018)
		3) Trauma related difficulties consistent with PTSD (mood related difficulties) (Reinhardt et al., 2023)
	Lowered self esteem	25) Lowered self-esteem and self-worth (Brownstone et al., 2018)
		26) Body dissatisfaction following MST (Brownstone et al., 2018)

#### Mental health impacts

MST was linked to a range of mental health symptoms, including depression (Reinhardt, McCaughey, Vento, & Street, 2023) and PTSD (Brownstone, Gerber, Holliman, & Monteith, 2018) (Katz, Huffman, & Cojucar, 2017; Reinhardt et al., 2023). Anxiety was linked to safety concerns around people and revictimisation fears (Katz et al., 2017; Reinhardt et al., 2023), leading to isolation (Reinhardt et al., 2023). Suicidal ideation and attempts appeared linked to difficulty coping with MST-related distress (Katz et al., 2017; Reinhardt et al., 2023). Whilst some participants experienced posttraumatic growth (Katz et al., 2017; Reinhardt et al., 2023), this appeared contingent on mental healthcare or effective social support.

#### Maladaptive coping

Motivations to escape distressing symptoms (Brownstone et al., 2018) appeared to promote risky behaviours like reckless driving (Brownstone et al., 2018; Reinhardt et al., 2023), physical altercations (Reinhardt et al., 2023), and risky sexual behaviours (Brownstone et al., 2018; Reinhardt et al., 2023). Disordered eating (Brownstone et al., 2018) and substance use (Katz et al., 2017; Reinhardt et al., 2023) also emerged as coping methods, with levels of substance use described as proportional to the salience of negative memories (Reinhardt et al., 2023).

#### Negative changes to self-perception

Self-blame was linked to disordered eating aimed at weight gain to prevent sexual attention (Reinhardt et al., 2023). Self-blame and feelings of powerlessness (Brownstone et al., 2018; Reinhardt et al., 2023) also related to disordered eating through lowered self-esteem, including body dissatisfaction (Brownstone et al., 2018).

#### Support from others is important for mental health

Although social support emerged as beneficial for coping with poor mental health (Brownstone et al., 2018; Katz et al., 2017), decreased interpersonal trust (Reinhardt et al., 2023) appeared to prevent engagement with informal networks. Participants in one study reported positive psychotherapy treatment experiences (Katz et al., 2017), however, evidence was limited by potential bias arising from participants via the psychotherapy service.

## Discussion

This review identified 63 papers (58 US-based) investigating mental health outcomes associated with MST in serving and ex-servicewomen. Quantitative studies identified associations between MST and adverse mental health, with qualitative studies adding contextual insight. MSA was most strongly linked with poor mental health. Experiencing additional traumas often amplified mental health impacts, whilst social support appeared to mitigate poor outcomes.

### Mental health outcomes

Consistent with wider sexual violence literature (Chen et al., 2010; Dworkin, 2020) and MST-specific research (Surís & Lind, 2008), PTSD and depression were commonly identified outcomes. Quantitative and qualitative studies identified them as contributors to additional adverse outcomes, including substance use (Banducci et al., 2019; Brownstone et al., 2018; Gradus et al., 2008; Katz et al., 2017), disordered eating (Brownstone et al., 2018; Sandhu et al., 2022), and suicidality (Blais & Geiser, 2019).

The mediating roles of PTSD and depression in the relationship between MST and substance use (Banducci et al., 2019; Gradus et al., 2008) were illustrated in qualitative studies as motivators for substance use to suppress symptoms. In line with self-medication models (Khantzian, 1997) (Hawn, Cusack, & Amstadter, 2020), quantitative and qualitative studies suggested a dose–response relationship, with higher severity and frequency of traumatic experiences (Banducci et al., 2019; Yalch et al., 2018) and salience of traumatic memories (Reinhardt et al., 2023) leading to higher substance use to cope with symptoms. This aligns with evidence that sustained disruptions to stress regulation increases risk for maladaptive coping methods like substance use (Sinha, 2024). Addressing PTSD and depression symptoms could therefore deter harmful substance use.

Aligning with general population research on trauma and disordered eating (Hayes, Linardon, Kim, & Mitchison, 2021), this review similarly identified several pathways between MST and disordered eating (Breland et al., 2018; Brownstone et al., 2018; Harned & Fitzgerald, 2002; Sandhu et al., 2022; Breland, Donalson, Dinh, & Maguen, 2018). Military-specific factors, like fitness and body composition requirements, restrictions on food choices and mealtimes, and potential repercussions for diagnosed disordered eating, may further shape the development and presentation of disordered eating (Gaviria & Ammerman, 2023), beyond the impacts of MST. Understanding how the military context influences disordered eating following MST may streamline the identification and treatment of disordered eating.

Findings from this review align with evidence suggesting trauma increases risk for suicide behaviors in military personnel (Williamson et al., 2024). As in general populations, where PTSD and depression resulting from sexual assault heighten women’s suicide risk (Ullman, 2004), distressing symptoms appeared to play a similar role in suicidality after MST (Blais & Geiser, 2019; Katz et al., 2017; Reinhardt et al., 2023). Addressing military-specific barriers to help-seeking, like stigma and the perceived incongruence between help-seeking and valued military characteristics (Williamson et al., 2025), may help deter the progression from MST-related distress to suicidality.

### The role of social support

Social support appeared to have protective effects, whilst detriments to military and non-military support networks (Banducci et al., 2019; Fontana & Rosenheck, 1998) were linked to PTSD (Laws et al., 2016; Smith et al., 2017; Smith et al., 2020; Webermann et al., 2023). Collectively, quantitative and qualitative studies suggest a cycle, where mental health issues deter social support engagement (Katz et al., 2017; Reinhardt et al., 2023), contributing to poorer mental health.

Institutional Betrayal has previously been highlighted as a barrier to formal mental healthcare (Holliday & Monteith, 2019), where MST may lead to disengagement with military-provided resources due to distrust in the military and feeling unsafe in military-associated healthcare services (Kelly, 2021). Compensatory efforts to overcome barriers to informal or formal support may prevent the development of PTSD and related outcomes (Bryan et al., 2015; Monteith et al., 2018).

### MST and non-military interpersonal trauma

Comparisons between MST and non-military interpersonal trauma highlight specific associations with MST. Unique associations with PTSD (Surís et al., 2004) and depression (Newins et al., 2021) support the idea that MST features, like Institutional Betrayal, may intensify PTSD and depression (Smith & Freyd, 2013). Barriers to reporting MST, like concerns of negative institutional reactions, may deter engagement with healthcare services and compound trauma-related distress (Christl, Pham, Rosenthal, & DePrince, 2024; Kelly, 2021). Although these studies did not directly measure Institutional Betrayal, it may help explain the unique associations observed with PTSD and depression. MST did not show distinct associations with suicidal ideation, which may be more influenced by psychiatric sequalae, like PTSD and depression (Blais & Geiser, 2019; Panagioti, Gooding, & Tarrier, 2009).

Consistent with general population sexual violence research (Classen, Palesh, & Aggarwal, 2005), MST alongside other traumas predicts poorer mental health (Banducci et al., 2019; Blais et al., 2023; Mercado et al., 2015; Yalch et al., 2018). Revictimisation may lead to increased shame and self-blame, as well as avoidance coping (Classen et al., 2005), which may increase propensity to poor mental health (Batchelder et al., 2018; Kline, Berke, Rhodes, Steenkamp, & Litz, 2018; Stewart, Strickland, Noguiera-Arjona, & Wekerle, 2024). High rates of revictimisation in serving and ex-servicewomen (Baca, Crawford, & Allard, 2021; Blais et al., 2023) motivate strategies to address additional risks resulting from multiple interpersonal trauma exposures.

### Limitations

#### Limitations of the review

Excluding grey literature opens the possibility of publication bias. Focusing exclusively on women’s experiences was necessary due to potentially unique outcomes but leaves men’s experiences unexplored. The heterogeneity of mental health outcomes, and correspondingly heterogenous measures, prevented a meta-analysis.

#### Limitations of the literature

Quantitative studies lacked consensus in their conceptualisations of MST, potentially contributing to some contradictory findings (Gorman et al., 2021; Hendrikx et al., 2023; Kearns et al., 2016; Moreau et al., 2022). Broader sexual violence research is similarly characterized by varied operationalisations of sexual harassment and assault (Dworkin et al., 2017) and highlights that using single-item measures, not validated measures, and measures which do not include behavioural definitions of MST may lead to inaccuracies (Dworkin et al., 2017). Patterns of underreporting in formal MST screening settings (Blais, Brignone, Fargo, Galbreath, & Gundlapalli, 2018; Hargrave, Danan, Than, Gibson, & Yano, 2023) also demand caution, particularly in studies using data from Veterans Health Administration screenings or military-administered surveys.

Most studies were US-based. The distinct US healthcare system for military and veteran populations limits the generalizability of this review to other countries. The small pool of qualitative studies limits insight into the lived experiences of women with MST experiences.

Only two studies investigated the experiences of lesbian, gay, bisexual, trans, queer/questioning, and other sexual and gender minorities (LGBTQ+) participants (Gorman et al., 2021; Lindsay et al., 2016), one of which included a sample of transgender women. This review therefore includes findings related to the experiences of both cisgender and transgender women. Experiencing MST alongside challenges specific to transgender women (e.g. related to identity, discrimination and minority status) require further research to understand potentially unique impacts. More broadly, research in LGBTQ+ groups is needed to explore how intersecting marginalized identities and minority stress (Binion & Gray, 2020) influences mental health following MST. Future research should also examine whether similar patterns occur amongst men within these minority groups.

Methodological quality across studies varied. Lower-quality studies were included to comprehensively cover the evidence base but were interpreted with caution.

### Implications

This review identified specific mental health impacts of MST and highlighted groups potentially at heightened risk for adverse mental health. Potentially protective effects of formal and informal support underscore the importance of engaging women with MST experiences in informal support networks and healthcare services. Further qualitative research is needed to understand how to promote this engagement and provide adequate support.

Calls for policy changes (Defence Committee, 2021) and Ministry of Defence taskforces aimed at tackling issues of violence against women in the UK Armed Forces (Ministry of Defence & Carns, 2025) require a research-informed approach. The lack of UK-based research underscores the need for further studies that could enhance existing UK initiatives and training resources for people working with women with potential MST histories (Combat Stress, 2023) to meet the needs of UK serving and ex-servicewomen.

## Conclusion

This large systematic review of qualitative and quantitative studies highlights several adverse mental health outcomes associated with MST. Disordered eating, suicidality, and substance use are linked to PTSD and depression symptoms associated with MST, underscoring the importance of early intervention for PTSD and depression. Serving and ex-servicewomen with MSA experiences and with multiple interpersonal trauma exposures may face heightened risk of poor mental health, motivating targeted support for these groups. Overcoming barriers to informal and formal support is essential to maximise protective effects. Findings underscore the critical need for proactive strategies and policies to prevent MST.

## Supporting information

Obradovic et al. supplementary material Obradovic et al. supplementary material

## Supplementary material

The supplementary material for this article can be found at http://doi.org/10.1017/S003329172510175X.
