# Supplementary material for: Mental health outcomes associated with military sexual trauma in serving and ex-servicewomen: A systematic review
Source: Psychol Med. 2025 Sep 29;55:e287. doi: 10.1017/S003329172510175X (PMC12527520; doi:10.1017/S003329172510175X)
Supplement: Obradovic et al. supplementary material [file S003329172510175Xsup001.docx]

**Supplementary Materials**

**Supplementary Materials Table 1- Search Strategy for MEDLINE**

| Concept | Search # | Search Terms |
| --- | --- | --- |
| Women | 1 | exp Women/ |
|  | 2 | exp Female/ |
|  | 3 | Wom?n.mp |
|  | 4 | Female*.mp |
|  | 5 | Service-Wom?n.mp |
|  | 6 | feminin*.mp |
|  | 7 | 1-6 (OR) |
| Military | 8 | exp Military Personnel/ |
|  | 9 | exp Veterans/ |
|  | 10 | Armed Force*.mp |
|  | 11 | Military Personnel.mp |
|  | 12 | Service Personnel.mp |
|  | 13 | Veteran*.mp |
|  | 14 | Soldier*.mp |
|  | 15 | Navy.mp |
|  | 16 | Coast Guard.mp |
|  | 17 | Army.mp |
|  | 18 | National Guard.mp |
|  | 19 | Marine*.mp |
|  | 20 | Air Force.mp |
|  | 21 | Ex-Service*.mp |
|  | 22 | Military.mp |
|  | 23 | 8-22 (OR) |
| Sexual Trauma | 24 | exp Sexual Trauma/ |
|  | 25 | exp Sex Offenses/ |
|  | 26 | Military Sexual Trauma.mp |
|  | 27 | MST.mp |
|  | 28 | MSA.mp |
|  | 29 | MSH.mp |
|  | 30 | Sexual* Assault*.mp |
|  | 31 | Sexual* Harass*.mp |
|  | 32 | Sexual Violence.mp |
|  | 33 | Sexual* Abuse*.mp |
|  | 34 | Rape*.mp |
|  | 35 | Sexual Coercion.mp |
|  | 36 | Sexual Victimi?ation.mp |
|  | 37 | Sexual Trauma.mp |
|  | 38 | 24-37 (OR) |
| Mental Health Outcomes | 39 | exp Mental Health/ |
|  | 40 | exp Mental Disorders/ |
|  | 41 | exp Self-Injurious Behavior/ |
|  | 42 | Mental Health.mp |
|  | 43 | Wellbeing.mp |
|  | 44 | Well?being.mp |
|  | 45 | Mental* Ill*.mp |
|  | 46 | Mental Disorder*.mp |
|  | 47 | Distress.mp |
|  | 48 | Psychiatric.mp |
|  | 49 | Psychological.mp |
|  | 50 | Posttraumatic Stress Disorder*.mp |
|  | 51 | Post-traumatic Stress Disorder*.mp |
|  | 52 | PTSD.mp |
|  | 53 | Posttraumatic Stress Symptom*.mp |
|  | 54 | Post-Traumatic Stress Symptom*.mp |
|  | 55 | PTSS.mp |
|  | 56 | C-PTSD.mp |
|  | 57 | CPTSD.mp |
|  | 58 | Traumatic Stress.mp |
|  | 59 | CMD*.mp |
|  | 60 | Common Mental Disorder*.mp |
|  | 61 | Depress*.mp |
|  | 62 | DD.mp |
|  | 63 | MD.mp |
|  | 64 | MDD.mp |
|  | 65 | Anxiety.mp |
|  | 66 | AD.mp |
|  | 67 | GAD.mp |
|  | 68 | Substance Us*.mp |
|  | 69 | Substance Misuse.mp |
|  | 70 | Substance Abuse.mp |
|  | 71 | SUD.mp |
|  | 72 | Substance Dependenc*.mp |
|  | 73 | Addict*.mp |
|  | 74 | Drug Us*.mp |
|  | 75 | Drug Misuse.mp |
|  | 76 | Drug Abuse.mp |
|  | 77 | DUD.mp |
|  | 78 | Drug Dependenc*.mp |
|  | 79 | Alcohol Us*.mp |
|  | 80 | Alcohol Misuse.mp |
|  | 81 | Alcohol Abuse.mp |
|  | 82 | Alcohol*.mp |
|  | 83 | AUD.mp |
|  | 84 | Alcohol Dependenc*.mp |
|  | 85 | Hazardo?s Drinking.mp |
|  | 86 | Hazardo?s Alcohol.mp |
|  | 87 | Opioid Us*.mp |
|  | 88 | Opioid Abuse.mp |
|  | 89 | Opioid Misuse.mp |
|  | 90 | Opioid Dependenc*.mp |
|  | 91 | Heroin Us*.mp |
|  | 92 | Morphine Dependenc*.mp |
|  | 93 | Substance-related disorder*.mp |
|  | 94 | Drug-related disorder*.mp |
|  | 95 | Alcohol-related disorder*.mp |
|  | 96 | Eating Disorder*.mp |
|  | 97 | Disordered Eating.mp |
|  | 98 | Anorexi*.mp |
|  | 99 | Bulimi*.mp |
|  | 100 | BN.mp |
|  | 101 | Binge Eating Disorder.mp |
|  | 102 | BED.mp |
|  | 103 | Suicid*.mp |
|  | 104 | Self-Injur*.mp |
|  | 105 | Self-Harm*.mp |
|  | 106 | 39-105 (OR) |
| Women + Military + Sexual Trauma + Mental Health Outcomes | 107 | 7 AND 23 AND 38 AND 106 |

**Supplementary Materials Table 2- Measures and Definitions of MST Employed by Quantitative Studies**

| Author(s), Year  *Country* | Study Information | Construct | Definition | Measure | Psychometric Properties | Scoring | Rate |
| --- | --- | --- | --- | --- | --- | --- | --- |
| (Banducci et al., 2019)  *United States* | Cross-sectional study design;  Male and female veterans were recruited from the Veterans Health Administration (VHA) Environmental Epidemiology Service's roster of OEF/OIF Veterans. Participants completed self-report surveys. | MST  (MST as an overarching construct used in main analyses; MSA and MSH rates reported separately) | *“unwanted sexual behaviors[…]from military personnel […] during deployment” (*Banducci et al., 2019, p2) | DRRI-1 (King et al., 2003) | α =0.84 | Likert scale measuring frequency (1= “never” to 4= “many times”); higher scores indicate higher frequency. | **MSA:** 25%  **MSH:** 50% |
| (Blais et al., 2019)  *United States* | Cross-sectional;  Data were extracted from a larger dataset of partnered female serving and ex-serving military personnel, who had complete data for MST experiences, dependent variables (PTSD, depression, sexual satisfaction and function, suicidal ideation) and covariates. Recruitment for this was through Facebook advertisements and online listservs. | Harassment-only MST and Assault MST | *“psychological trauma, which[…]resulted from a physical assault of a sexual nature, battery of a sexual nature, or sexual harassment which occurred while the Veteran was serving on active duty or active duty for training” (*US Government, 2014, p. 285) | VA 2- item MST screening tool (modified). Assault MST was assessed using the following item: *“Did someone ever use force or threat of force to have sexual contact with you against your will?”.* Harassment-only MST was assessed with a checklist of possible experiences. | N.R. | Dichotomous (Harassment-only and Assault MST scored separately. Endorsement of relevant items was coded positively). | **Any MST:** 82.5%  **Harassment-only MST:** 58.23%  **Assault MST:** 41.77% |
| (Blais & Geiser, 2019)  *United States* | Cross-sectional.  Data were extracted from a larger dataset of partnered serving and ex-serving personnel. | Harassment-only MST and Assault MST | *“psychological trauma, which[…]resulted from a physical assault of a sexual nature, battery of a sexual nature, or sexual harassment which occurred while the Veteran was serving on active duty or active duty for training” (US Government, 2014, p. 285)* | VA 2-item MST screening tool (modified). Assault MST was assessed using the following item: *“Did someone ever use force or threat of force to have sexual contact with you against your will?”* (Blais & Geiser, 2019, p149). Harassment-only MST was assessed with a checklist of possible experiences. | N.R. | Dichotomous (Harassment-only and Assault MST scored separately. Endorsement of relevant items was coded positively). | **Harassment-only MST:** 47.6%  **Assault MST:**  34.0% |
| (Blais et al., 2023)  *United States* | Cross-sectional.  Data for participants who were aged 18-65 years and partnered at the time of participation were extracted from two gender-specific parent studies with samples of partnered men and women service-members/ veterans. These parent studies employed convenience sampling using Facebook advertisements and online listservs. | Military Sexual Violence and Revictimisation (pre-military sexual violence and Military Sexual Violence) | Sexual harassment (defined as experiences including cornering, pressure for sexual favours) and/or sexual assault (e.g., unwanted touching, rape) during military service. | VA 2-item MST screening tool (modified) | N.R. | Dichotomous | **Military Sexual Violence only:** 29.79%  **Revictimisation**: 52.01% |
| (Breland et al., 2018)  *United States* | Cross-sectional.  Women veterans were recruited via an urban outpatient VA medical centre and associated community outpatient clinics. Eligible participants (aged 18-70, no history of a psychotic disorder or suicide attempt in the past 5 years) completed self-report surveys. | MST | “*sexual harassment (unwanted sexual attention, like verbal remarks, touching, or pressure for sexual favours) and sexual assault (use of force, threat of force, or coercion used for sex against one’s will) (Breland et al., 2018, p2)” during military service.* | VA 2-item MST screening tool | N.R. | Dichotomous | 66% |
| (Bryan et al., 2015)  *United States* | Cross-sectional.  Military personnel or veteran enrolled in college or university classes in the US completed an online survey. | MST (any), sexual assault during military service and/ or other unwanted  sexual experiences during military service. | *“sexual assault […] and/or other wanted sexual experiences that occurred during military service” (Bryan et al., 2015, p248)* | Sexual victimization items from the LEC (Gray et al., 2004) | N.R | Dichotomous | **Any MST**: 39.10%  **Unwanted sexual experiences:** 36.8%  **Sexual assault:** 18.3% |
| (Chang et al., 2001)  *United States* | Cross-sectional.  A random sample, recruited from the VA Women’s Health Project, completed self-report surveys. Eligible participants were women veterans who had received ambulatory care from any of the 158 included hospitals between July 1, 1994, to June 30, 1995. | MSA | Sexual assault, “*where someone used force or the threat of force to have sexual relations with [someone] against [their] will while in the military” (Chang et al., 2001, p80)* | Single item: *“Did you ever have an experience where someone used force or the threat of force to have sexual relations with you against your will while in the military?” (Chang et al., 2001, p80)* | N.R. | Dichotomous | 23% |
| (Cobb Scott et al., 2014)  *United States* | Cross-sectional.  Female veterans were recruited from an ongoing prospective cohort study of OEF/OIF veterans and completed self-report surveys. Eligible participants were females who had separated from OEF/OIF military service and enrolled in VA health care between October 1, 2001, and April 30, 2008, and who lived within 300 miles of the VA New England and VA Indiana regions. | MST | Sexual assault or threatening sexual harassment during military service. | VA 2 item MST screening tool | N.R. | Dichotomous | 49.4% |
| (Decker et al., 2021)  *United States* | Cross-sectional.  Data were extracted from the OEF/OIF/OND Roster. Eligible participants were OEF/OIF/OND era veterans who first used the VHA in 2008–2013, completed the PHQ-9, who did not have evidence of a mental health condition on or before the PHQ-9 administration and who receive care at VHA facilities which reported depression screening rates below 75%. | MST | Sexual assault or sexual harassment during military service. | VA 2 item MST screening tool | N.R. | Dichotomous | 27.9% |
| (Dutra et al., 2011)  *United States* | Cross-sectional.  Convenience sampling was employed by recruiting participants from waiting rooms of an army medical clinic during a post-deployment wellness visit in Oahu, Hawaii following deployment to Iraq. Consenting participants completed assessment interviews before or after their medical appointments. | MSH | *“sexual harassment or abuse by their unit leaders or other unit members during deployment” (Dutra et al., 2011, p30)* | DRRI-1 (King et al., 2003) | N.R. | Continuous (Participants rate frequency of behaviours (0 (never) – 3 (many times) on a Likert scale) | 57.4% |
| (Esopenko et al., 2023)  *United States* | Cross-sectional  Data were from a larger study of clinical outcomes of trauma-focused care within a 4-week VA acute psychiatric inpatient program (participants were enrolled between December 2009 and March 2013). Eligible participants were female veterans with PTSD diagnoses who had been identified as having limited progress following a less intensive outpatient PTSD treatment. | MST | Sexual harassment or non-partnered sexual violence during military service. | VA 2 item MST screening tool | N.R. | Dichotomous | 80.5% |
| (Fillo et al., 2023)  *United States* | Longitudinal.  Data extracted from an ongoing longitudinal study (Soldiers and Families Excelling Through the Years (SAFETY), which recruited participants from units across New York State and involved a baseline survey and two annual follow-up surveys. To be eligible, participants were required to be currently serving (at baseline), US army and national guards reserves, part of a couple (married/ living as married) in which one partner was a current US Army Reserve/ National Guard soldier, the soldier was 18-45 yrs old and both partners were required to speak and understand English and to have had at least one alcoholic drink in the previous year. | MST (Time 1- MST during deployment; Time 2 and Time 3- MST during military service generally) | *“Unwanted physical contact (e.g., touching, assault, rape) and verbal remarks (e.g., comments, coercion, threats) of a sexual nature*” (Filo et al., 2023, p737) during military service. | DRRI-2 (Vogt et al., 2013) (modified at Time 2 and Time 3). | N.R. | Dichotomous | 61.4% |
| (Fontana & Rosenheck, 1998)  *United States* | Cross-sectional.  Participants were recruited from VA women's stress disorder treatment outpatient clinics between May 1, 1994, and Jan 30, 1997. Eligible participants were women veterans treated in one of the four selected VA clinical programs for women with stress disorders who had complete data for the variables included in the model | Military Sexual Stress (investigated as an overarching construct in main analyses; rates of sexual harassment and attempted/ completed rape reported separately) | Verbal or physical sexual harassment. | Military Stress Inventory for Women (4-item sub-scale) (Wolfe et al., 1993) | α = .72 | Sum of 5-point Likert scale items assessing frequency | **Sexual harassment:**  63%  **Attempted/ completed rape:**  43% |
| (Gibson et al., 2019)  *United States* | Cross-sectional.  Analyses used International Classification of Diseases, Ninth Revision, Clinical Modification  (ICD-9, CM) and VA MST codes from clinical visits, obtained from the VA National Patient Care Database electronic medical records. Eligible participants were women veterans aged 55 years or older, enrolled in the VA since 2005, with at least one VA clinical encounter and documented response to MST screening in fiscal years 2005-2015. Only those whose frequency of VA encounters was in top 10% of the sample were included in analyses. | MST | Sexual harassment or sexual assault whilst on active duty. | VA 2 item MST screening tool | N.R. | Dichotomous | 13.4% |
| (Gorman et al., 2021)  *United States* | Cross-sectional  Participants were recruited from a longitudinal study (Veterans After-discharge Longitudinal Registry; Project VALOR). Data were collected using self-report surveys and telephone structured clinical interviews. Eligible participants were OIF/OEF-deployed army or marine veterans, who were deployed to combat and who received a VHA mental health evaluation between Jun 2008- Dec 2009 | MSA | Attempted and/or completed rape whilst on deployment. | MSA items from the DRRI-2 (Vogt et al., 2013) | α = .84 | Participants rated items on a 4-point scale (1 (never) to 4 (many times)). Responses were summed, with higher scale scores indicating higher severity | **Sexual minority female veterans:**  39%  **Heterosexual female veterans:**  47% |
| (Gradus et al., 2008)  *United States* | Cross-sectional.  Former reservists were recruited via the Defense Manpower Data Center and completed telephone interviews | Sexual harassment during military service | Sexual harassment during military service. | SEQ-DoD (Fitzgerald et al., 1999) | α = .81 | N.R. | 72.5% |
| (Gradus et al., 2017)  *United States* | Cross-sectional.  Participants were recruited from the VHA Environmental Epidemiology Service roster and completed self-report surveys. Eligible participants were those deployed in support of the conflicts in Iraq and Afghanistan. Participants were excluded if they reported suicidal ideation with no or few current mental health symptoms or reported more severe suicidal behaviour. | Sexual harassment during deployment | Sexual harassment/ assault during deployment. | DRRI-1 (King et al., 2003) | α = .84 | Dichotomous (any endorsement of DRRI items) | N.R. |
| (Gross et al., 2018)  *United States* | Cross-sectional.  Data collected as part of the VA Mid-Atlantic Mental Illness Research, Education and Clinical Center Post Deployment Mental Health Study. Participants were recruited from four VA sites in North Carolina and Virginia and completed self-report surveys and diagnostic interviews. Eligible participants were female Iraq/Afghanistan Veteran who completed the MSA assessment. | Deployment-related MSA | *“unwanted sexual activity as a result of force, threat of harm, or manipulation”* (Gross et al., 2018, p5) during deployment. | Single item: participants were asked if they had experienced *“unwanted sexual activity as a result of force, threat of harm, or manipulation”* (Gross et al., 2018, p5) during deployment | N.R. | Dichotomous | 12.7% |
| (Gross et al., 2020)  *United States* | Longitudinal.  Secondary data analysis of a 3-year longitudinal study. Eligible participants were pregnant women veterans enrolled at one of the selected 15 VA medical centres from which participants were recruited. Data collected using telephone interviews. Prenatal interviews took place from January 2016. Postnatal interviews took place between from July 201. | MST-harassment and MST-assault | Sexual harassment or sexual assault whilst in military service. | VA 2 item MST screening tool | N.R. | Dichotomous  (MST-harassment and MST-assault scored separately) | **MST-harassment:**  51.5%  **MST- assault:** 29.8% |
| (Hankin et al., 1999)  *United States* | Cross-sectional.  Participants were recruited using a random sampling frame from lists provided by 158 VA hospitals and completed self-report surveys. To be eligible participants were required to be female veterans, VA patients who had made at least one outpatient visit between July 1, 1994, and June 30, 1995, had made at least one outpatient visit between July 1, 1994, and June 30, 1995, community residing, and have complete addresses. | Sexual assault during military service | Sexual assault during military service. | Single item: *“Did you ever have an experience where someone used force or the threat of force to have sexual relations with you against your will while you were in the military?’* (Hankin et al., 1999, p604) | N.R. | Dichotomous | 23% |
| (Harned & Fitzgerald, 2002)  *United States* | Cross-sectional  Eligible participants (active-duty military women, who were posted at one of two U.S. military installations and selected to participate in a DoD gender issues pilot survey) completed self-report surveys | Sexual harassment in the military workplace. | *“unwanted sex-related behaviours in the previous 12 months from military personnel or civilian employees or contractors employed in the military workplace”* (Harned & Fitzgerald, 2002, p1175) | SEQ- DoD (short form) (Fitzgerald et al., 1999) | α = .89 | 5-point scale (0 (never) – 4 (many times)) | 48% |
| (Harned et al., 2002) *United States* | Cross-sectional  Data from the 1995 DoD Gender Issues Survey. Eligible participants were women DoD and Coast Guard members, except flag rank officers, with at least 6 months of active-duty service. | Sexual assault/ sexual harassment by military workplace personnel. | **Sexual harassment: *“****unwanted sex-related experiences in the military during the previous 12 months that were perpetrated by military personnel (on or off duty, or on or off base/post) or civilian employees/contractors employed in the military workplace.”* (Harned et al., 2002, p177).  **Sexual assault*:*** Attempted and/or completed by military workplace personnel. | SEQ-DoD (Fitzgerald et al., 1999) | α = 0.83-0.94 | Participants respond using a 5-point Likert-type scale (0 (never) – 4 (very often)). | **Sexual harassment:** 72.4%  **Sexual assault:** 4.2% |
| (Hendrikx et al., 2023)  *United Kingdom* | Cross-sectional.  Surveys were completed by women connected with a women veteran’s charity between August and October 2020. Eligible participants were veterans connected with the charity, who consented to be contacted and provided an email address | Sexual harassment and sexual assault | Sexual victimisation during military service. | N.R. | N.R. | Dichotomous (sexual harassment and sexual assault scored separately) | **Sexual harassment:** 22.5%  **Sexual assault:** 5.1% |
| (Himmelfarb et al., 2006)  *United States* | Cross-sectional.  Eligible participants (female veterans) were recruited via the Women’s Comprehensive Healthcare Program at the VA West Los Angeles Healthcare Center or via postal invite to female veterans in the metropolitan area (between December 2000 and December 2002). Participants completed self-report questionnaires and structured interviews | MST | *“sexual trauma that occurred during a woman’s military service, including forced intercourse, anal or oral sex, insertion of objects […] verbal harassment without threats of force [was not considered] to be sexual trauma”* (Himmelfarb et a;., 2006, p838). | Stressful Life Events Questionnaire (Goodman et al., 1998) and interviewer follow up. | Good test-retest reliability (median χ = .73); Acceptable convergent validity (median χ = .64 | Dichotomous (based on interviewer severity ratings of participant descriptions, with experiences with scores of 8 or higher being coded as MST). | 41% |
| (Hoffmire et al., 2021)  *United States* | Cross-sectional  Analyses used baseline data from a longitudinal study (collected between 2012 and 2015) focused on post-deployment experiences and coping among  previously deployed post-9/11 era veterans. Recruitment targeted who had separated from the military in the past 5 years. Participants completed self-report surveys. | Deployment Sexual Trauma | N.R. | DRRI-2 (Vogt et al., 2013) | N.R. | Continuous scale scores (higher scale scores indicating higher severity and frequency) | 11.2% |
| (Kang et al., 2005)  *United States* | Nested case–control analysis.  Analysis of data collected for the National Health Survey of Gulaf War Era Veterans and Their Families (a population-based survey collected in 1996). Eligible participants were US veterans who served during the Gulaf War. Gulf War veterans with current PTSD (n= 1381) were compared with Gulf veteran controls without PTSD (n= 10,060) | In-theatre experiences of sexual harassment and sexual assault | Sexual assault and harassment during deployment. | *‘‘While in the Persian Gulf, do you believe you were exposed to or did you experience any of the following... (1) ‘‘suffered forced sexual relations or a sexual assault’’*; and (2) ‘‘*experienced sexual harassment.’’* | N.R. | Dichotomous (sexual harassment and sexual assault scored separately) | **Sexual assault:** 3.3%  **Sexual harassment:**  24% |
| (Kearns et al., 2016) *United States* | Cross-sectional.  Self-report questionnaires and interviews completed as follow up for Veterans After-discharge Longitudinal Registry; Project VALOR. Eligible participants were army or marine veterans, who had completed a mental health evaluation at a VA facility. Veterans with probable PTSD were oversampled to create a 3:1 (probable PTSD: no PTSD). Participants were recruited from a roster provided by the VA Environmental Epidemiology Service. Participants completed self-report questionnaires | MSA | *“intentional sexual contact characterized by the use of force, threats, intimidation, or abuse of authority or when the survivor does not or cannot consent that has occurred at any point during active-duty military service”* (Kearns et al., 2016, p2) | DRRI-2 (Vogt et al., 2013) | α = .86 | Participants rate frequency on a 4-point Likert scale 1 (never) to 4 (many times).  Higher total scores indicated higher severity. A score > 4 was the cut off for having experienced MSA. | 45.7% |
| (Kim et al., 2017) *Republic of Korea* | Cross-sectional  Data from a Military Health Survey conducted in 2014 | Sexual Harassment during military service | *“(a) gender harassment, generalised sexist remarks and behaviour; (b) seductive behaviour, inappropriate and offensive, but essentially sanction-free, sexual advances; (c) sexual bribery, solicitation of sexual activity or other sex-linked behaviour with promise of rewards; (d) sexual coercion, pressure for sexual activity by threat of punishment and (e) sexual assault, gross sexual imposition or assault”* during the period of military service. (Kim et al., 2017, p107)` | N.R. | N.R. | Dichotomous | 5.7% |
| (Kimerling et al., 2007) *United States* | Cross-sectional.  Data analysis of VHA administrative data (ICD-9 codes) from a national sample of VHA outpatients. Eligible participants were VHA outpatients who had at least 1 outpatient visit to a VHA health care facility that reported valid MST monitoring data during fiscal year 2003. | MST | *“severe or threatening forms of sexual harassment and sexual assault sustained in military service”* (Kimerling et al., 2007, p2160) | VA 2 Item MST screening tool | **MSH item:**  *sensitivity*=.92; *specificity= .*89  **MSA item:** *sensitivity*= .89; *specificity=* .90 | Dichotomous | 22% |
| (Kimerling et al., 2010) *United States* | Cross-sectional.  Data were extracted from VA electronic medical records. Eligible participants were veterans deployed in service of Operation Enduring Freedom and Operation Iraqi Freedom and separated from military service by September 30, 2006, who used Veterans Health Administration mental health or primary care services between October 1, 2001, and September 30, 2007, and screened for MST. | MST | N.R. | VA 2 Item MST screening tool | N.R. | Dichotomous | 15.1% |
| (Laws et al., 2016)  *United States* | Cross-sectional.  Data from the Survey of Experiences of Returning Veterans’ structured phone interviews collected within 5 years of separation from service. | MST during deployment | N.R. | DRRI-2 (Vogt et al., 2013) | α = .835 | Participants rated frequency of experiences (0 (Never) to 3 (Many times). | N.R. |
| (Lindsay et al., 2016) *United States* | Cross-sectional.  Analysis of VA administrative data (ICD-9 codes) from the VHA Medical Statistical Analysis System file, covering VHA inpatient and outpatient medical and mental health treatment (from October 1, 2000, and September 30, 2013). Eligible participants were Transgender veterans in the VHA, who served during the Iraq and Afghanistan conflicts. | MST | N.R. | VA 2 item MST screening tool | N.R. | Dichotomous | 13.5% |
| (Luterek et al., 2011) *United States* | Cross-sectional.  Participants were recruited from the VA Puget Sound Health Care System outpatient general mental health clinic and specialized mental health clinic for PTSD (50%) and completed self-report surveys and interviews Eligible participants were enrolled in VA Puget Sound Health Care System for at least 12 months, at least two mental health visits in the past year, being actively followed in the general mental health or PTSD clinic at the time of assessment, determined as stable by clinicians, not engaged in addictions treatment or the outpatient clinic serving veterans with severe chronic mental illness. | MSA | Sexual assault whilst on active duty. | TLEQ (Kubany et al., 2000). Participants specified time period of the experience. | N.R. | Dichotomous | 63.5% |
| (Maguen et al., 2012) *United States* | Cross-sectional.  Retrospective data analyses conducted using VA administrative data. Eligible participants were Iraq and Afghanistan veterans, with at least one primary care or mental health visit to a VA facility between April 1, 2002 - October 1, 2008, and who were new users of VA health care. | MST | N.R. | VA 2 item MST screening tool | N.R. | Dichotomous | 31% |
| (Mahoney et al., 2024) *United States* | Longitudinal.  Self-report surveys completed at Time 1 and Time 2 (12 months later). Participants were recruited from a larger survey study. To be eligible, participants had to be females, aged at least 18 years of age, and using VHA care in the New England region within the prior year (determined by VHA administrative data). | MST | *“experiencing sexual harassment, unwanted sexual contact, attempted rape, or completed rape during active duty”.* (Mahoney et al., 2024, p17) | VA 2 item MST screening tool  (assessed at Time 1) | N.R. | Dichotomous | 54.1% |
| (Mercado et al., 2015) *United States* | Cross-sectional.  Participants were recruited (~ fiscal year 2011) from a random sample of female veterans who were VA patients in the New England region (N= 700) completed self-report paper-and-pencil mail survey. Eligible participants were women, aged at least 18 years, enrolled as a veteran patient (not dependent) in the VA New England Healthcare System, and had attended one or more VA medical or mental health appointments within the last year. | IPV-related MST and non-IPV-related MST | Sexual assault or repeated, threatening sexual harassment that occured during military service. | VA 2-Item MST screening tool | N.R. | Dichotomous | 49% |
| (Monteith et al., 2018) *United States* | Cross-sectional  Secondary analysis of baseline interview data from the Survey of Experiences of Returning Veterans. Eligible participants were veterans separated from the US military, who served in OEF/OIF/OND, aged 18 or older, English-speaking, and currently living in the US. | Deployment Sexual Trauma | *“psychological trauma due to sexual assault or sexual harassment while on active duty, active duty for training, or inactive duty training (U.S. Government, 2014)”,* which occurred during deployment. | DRRI-2 (Vogt et al., 2013) | α = 0.91 | Participants rate frequency of experiences (1 (never) – 4 (many times). Responses are summed, with higher scores indicating more frequent and severe DST. | N.R. |
| (Monteith et al., 2023) *United States* | Cross-sectional.  Secondary analysis of survey data. To be eligible, participants had to: be women veterans, separated from military service between October 1, 2009, and September 30, 2018, be of reproductive age (18–44 years) at separation, and used VHA provided reproductive healthcare in fiscal year 2018. | MSH and MSA | *“psychological trauma that was caused by sexual assault and/or sexual harassment that occurred during one’s military service (U.S. Government, 2021)”* | VA 2 item screening tool | N.R. | Dichotomous (MSH and MSA scored separately) | **MST:** 68.7%  **Military sexual harassment:**  67.2%  **Military sexual assault:** 42.5% |
| (Moreau et al., 2022) *France* | Cross-sectional.  Analysis used data from a national sexual health survey in the French military (conducted in 2014-2015). Recruitment employed two-stage random sampling strategy, where 18 military units were randomly selected. 120 individuals per unit were randomly selected and invited by the unit commander to attend an information session | MST in the last 12 months (separated into (1) sexual oppression, (2) sexual coercion, (3) repeated verbal unwanted sexual attention, (4) sexual assault) | *“unwelcomed sexual advances, requests for sexual favours and other verbal, behavioural and or physical conduct of a sexual nature […]as well as attempted or forced sexual contact”* (Moreau et al., 2022, p2) during military service. | Composite measure using items from the SEQ-DoD (Fitzgerald et al., 1999)and the French National Survey on Sexual Violence. | N.R. | Dichotomous and severity scoring based on a cumulation of MST experiences, including: 1) no MST 2) MST in the form of repeated sexual comments alone, 3) MST involving 1 type of sexual oppression (either coercion or repeated verbal unwanted sexual attention or sexual assault), 4) MST involving several types of sexual stressors including at least one form of sexual oppression.) | **Any MST:** 35.8%  **Sexual comments alone:**  20.9%  **Sexual oppression:** 14.9%  **Multiple sexual stressors:**  10.3% |
| (Murdoch et al., 2006) *United States* | Cross-sectional.  Veterans who had previously filed VA PTSD disability benefit claims were sent self-report surveys approximately 2 years after filing claims. | In-service sexual harassment | Sexual harassment during military service. | 21-Item SHI (Murdoch & McGovern, 1998) | α=0.92–0.95 | Continuous; severity weighted scores (0 (no harassment) to 39.52 (greatest severity/all behaviours endorsed) | 93% |
| (Murdoch et al., 2010) *United States* | Cross-sectional  Participants were recruited from the VA enrolment database and invited to complete self-report questionnaire Eligible participants were those confirmed as active duty between January 1998 and June 2002 in a VA database. | Military sexual stress (three categories: sexual harassment, sexual assault, and sexual identity challenges. ) | Occupational sexual stressors (in the military). | 21-Item SHI (Murdoch & McGovern, 1998) and Gender role enforcement subscale of the Sexual Harassment of Men scale (modified) | *α* = 0.92–0.95 | 3 Categories based on hypothesised severity ((*1*) sexual identity challenges or sexual harassment only, (*2)* sexual identity challenges plus sexual harassment, (3) sexual assault plus sexual harassment with or without sexual identity challenges) | **Sexual identity challenges/ sexual harassment only:**  40.6%  **Both sexual identity challenges/ sexual harassment**  27.5%,  **Sexual harassment and assault:**  11.8%, |
| (Murray-Swank et al., 2018)  *United States* | Cross-sectional.  Participants were sent self-report surveys. Eligibility: Women veterans who had a “rural” or “highly rural” zip code, availability to attend one wellness retreat, and psychological capacity to participate in a residential, wellness-based program. No acute medical health conditions (e.g., need for oxygen, severe heart condition), acute suicidality (within past month), and current drug and/or alcohol abuse. | MST (sexual harassment and sexual assault) | Sexual harassment/ assault whilst in the military. | VA 2-item screening tool | N.R. | Dichotomous (sexual harassment and sexual assault scored separately) | **Any MST:** 68%  **Sexual harassment:** 67%  **Sexual assault:** 39% |
| (Newins et al., 2021)  *United States* | Cross-sectional.  Participants were recruited for the Post-Deployment Mental Health Study (Brancu et al., 2017) via mailings, VA clinician referrals, and advertisements. Data collection involved surveys and took place in four VA medical centres in Virginia and North Carolina between 2005 and 2015. Eligible participants were Iraq/Afghanistan-era U.S. military veterans, Active-Duty military personnel, and members of the Reserves and National Guard who served post-September 11th, 2001, whose primary language was English, could comprehend informed consent form and process, ability to travel to one of the data-collection sites. Individuals were not required to  be seeking mental health treatment (or any health care services). | MSA | Sexual assault during military service. | TLEQ sexual assault item. (Kubany et al., 2000) | N.R. | Dichotomous | 13.4% |
| (Rønning et al., 2024)  *Norway* | Cross-sectional  Secondary analysis using data from a post-deployment survey. Eligible participants were Norwegian military personnel deployed to Afghanistan between 2001 and 2020. | Workplace sexual harassment during deployment to Afghanistan or another military setting. | *“unwelcome verbal or physical contact of a sexual nature that is hostile in character and interferes with work performance”* (Rønning et al., 2024, p3). | Single item assessing sexual harassment during deployment to Afghanistan or in another military setting. | N.R. | Dichotomous | 14.3% |
| (Sandhu et al., 2022) *United States* | Cross-sectional  Self-report surveys. Recruitment conducted via Prolific. Eligible participants were US veterans who identified as female | MSA | Sexual assault in the military | Attempted rape and rape subscales of the modified SES | *α* =.93 | Dichotomous | 61% |
| (Skinner et al., 2000) *United States* | Cross-sectional  Data taken from the VA women's health project. Eligible participants (female veterans who had at least one ambulatory visit at any VA facility between July 1, 1994, and June 30, 1995) were sent a letter invitation. | Sexual harassment and sexual assault during military service | **Sexual harassment:** unwelcome sexual behaviour or hostile, offensive or degrading sexual behaviour.  **Sexual assault:** unwanted sexual experiences with the use of threat/ force. | Items consistent with the VA 2-Item MST Screening Tool | N.R. | Dichotomous (Sexual harassment and sexual assault scored separately) | **Sexual harassment:** 55%  **Sexual assault:** 23% |
| (Smith et al., 2017)  *United States* | Cross-sectional  Participants were recruited from a roster from the Defense Manpower Data Center, stratified by component of service (50% Active-Duty component and 50% National Guard/Reservist) and gender, with oversampling of women to comprise 50% of the roster. Eligible participants (veterans who had returned from deployment to Iraq or Afghanistan within the previous two years (2007–2009)) competed self-report surveys. | Sexual harassment during deployment | *“Unwanted sexual contact or verbal conduct of a sexual nature from other unit members, commanding officers, or civilians in the war zone.”* (Smith et al., 2017, p44). | DRRI-1 (King et al., 2003) | *α* = .84 | 4-point Likert type scale assessing frequency (1 = Never;4 = Many times), summed to create a total score | N.R. |
| (Smith et al., 2020)  *United States* | Longitudinal.  Self-report surveys completed at baseline and two additional follow-ups (3-months and 9-months). Participants were recruited from a larger Randomised Control Trial. Eligible participants worked at one of the 35 organizations participating in the Randomised Control Trial for at least 20 hours per week and had served in the US military post-9/11 era. | Harassment MST and Assault MST | *“psychological trauma . . . result[ing] from a physical assault of a sexual nature, battery of a sexual nature, or sexual harassment which occurred while the Veteran was serving on active duty, active duty for training, or inactive duty training” (*US Government, 2014, p. 285). | VA 2 Item Screening Tool, measured at Time 1, Time 2, Time 3. | N.R. | Dichotomous (Harassment MST and Assault MST scored separately) | N.R. |
| (Stefanovics et al., 2023)  *United States* | Cross-sectional  Self-report surveys were completed by participants. Participants were required to be US veterans to be eligible. Participants recruited from the National Health and Resilience in Veterans Study | MST | N.R. | VA 2-item screening tool | N.R. | Dichotomous | N.R. |
| (Street et al., 2008)  *United States* | Cross-sectional  Participants were recruited through a stratified random sampling design from a list of former reservists provided by the Defense Manpower Data Center. Sampling was stratified based on gender and reserve component. Eligible participants (former reservists who had not also served in the active-duty forces) completed self-report, computer-assisted telephone interviews. | Sexual harassment and  sexual assault during service (in an occupational context). | Sexual harassment and  sexual assault during service, where a participant was considered to have experienced sexual harassment if they endorsed at least four separate sexual harassment experiences or one experience considered as more severe, and sexual assault if they endorsed at least one experience of attempted/ completed rape or *“coerced genital fondling*” (Street et al., 2008, p413). | SEQ-DoD (modified) (Fitzgerald et al., 1999) | *α* = .81 | Dichotomous (sexual harassment and sexual assault scored separately) | **Sexual harassment:** 60.0% **Sexual assault:** 13.1% |
| (Sumner et al., 2021)  *United States* | Cross-sectional.  Analyses of VHA Electronic Medical records. Eligible participants were women veterans aged 18 years or older, who enrolled in the VHA between January 1, 2000, and December 31, 2017, had at least one VHA clinical encounter during the observation period, and completed the MST screen. | MST | Sexual harassment or sexual assault during military service. | VA 2-item screening tool | N.R. | Dichotomous | 26% |
| (Surís et al., 2004)  *United States* | Cross-sectional  Eligible participants (female veterans enrolled in a medical and/or mental health clinic within the VA North Texas Health Care System who were seen for at least one outpatient appointment during the 5 years before contact) were recruited via the Dallas VA Medical Center and mental health clinical within the centre (1997-2000). Analyses used data from participant interviews and medical records | MSA | *“Any type of sexual conduct, including vaginal, anal, or oral sex, achieved or attempted without the person’s consent and with the use of threat or force”* (Suris et al., 2004, p751) whilst on active duty. | Interview of Sexual Experiences (Surís et al., 2004) | Sensitivity= .97; specificity = .97. | Dichotomous | 33.0% |
| (Surís et al., 2007)  *United States* | Cross-sectional.  Eligible participants (female veterans who were enrolled in a medical or mental health clinic within the Veterans Administration North Texas and attended at least one outpatient appointment during the 5 years before contact) were recruited through a VA outpatient medical centre via clinician referrals, responses to in-hospital advertisements and appointment schedules. Participants completed self-report questionnaires and interviews. | MSA | *“Any type of sexual conduct, including vaginal, anal, or oral sex, achieved or attempted without the person’s consent and with the use of threat or force”* (Suris et al., 2007, p183). | Interview of Sexual Experiences (Surís et al., 2004) | Sensitivity= .97; specificity = .97. | Dichotomous | 33.0% |
| (Webermann et al., 2023)  *United States* | Longitudinal  Participants were recruited from a DoD roster between February 11, 2016, and October 28, 2019, and completed self-report surveys. Eligible participants were veterans with English literacy and who served in post-9/11 conflicts, who completed both baseline and follow-up surveys and had no missing data for the primary outcome variable (PTSD symptoms at follow-up) | MST (any), sexual harassment, sexual assault | Sexual trauma during military service. | VA 2-Item MST Screening tool (measured at Time 1) | N.R. | Dichotomous (sexual harassment and sexual assault scored separately) | **MST:** 30.4%  **Sexual harassment:** 29.1%  **Sexual assault:** 13.6% |
| (Wilson et al., 2020)  *United States* | Cross-sectional  Eligible participants (veterans who served in the U.S. military post-September 11, 2001, and completed MSA measures) completed self-report surveys and clinician interviews Data from the VA Mid-Atlantic Post-Deployment Mental Health study | MSA during post-9/11 deployment | “*unwanted sexual contact or sexual penetration during their post-9/11 deployment”* (Wilson et al., 2020, p4). | Single self-report item | N.R. | Dichotomous | 12.7% |
| (Wolfe et al., 1998)  *United States* | Cross-sectional  Participants were recruited from the Ft. Devens ODS Reunion Survey and completed additional self-report surveys | Sexual assault, physical sexual harassment and verbal sexual harassment during Persian Gulf deployment | **Sexual assault:**  *“a sexual experience that was unwanted and involved the use or threat of force (e.g., attempted rape or completed rape)”* (Wolfe et al., 1998, p44)  **Physical sexual harassment:**  *“unwanted sexual touching, fondling, cornering, or brushing against you”* (Wolfe et al., 1998, p44)  **Verbal sexual harassment:**  *“sexual remarks; sexually suggestive looks, gestures, or body language; pressure for sexual favours”* (Wolfe et al., 1998, p44) | Three items which asked participants about their experiences of sexual assault, physical sexual harassment and verbal sexual harassment during Persian Gulf deployment. | N.R. | Participants rated the frequency of experiences on a 4-point Likert scale (never- four or more times) | **Sexual assault:**  7.3%  **Physical sexual harassment:**  33.1%  **Verbal sexual harassment:**  66.2% |
| (Yaeger et al., 2006)  *United States* | Cross-sectional.  Participants were recruited from the Women’s Comprehensive Healthcare Center at the VA West Los Angeles or via letter (between December 2000 and December 2002. Eligible participants (presenting to the clinic for treatment, veteran. Did not have dementia, psychosis or suicidality) completed self-report surveys and interviews. | MST | *“forced intercourse or anal sex, forced oral sex, forced insertion of objects, or threats of forced sex”* (Yaeger et al., pS66) *during* military service. Verbal sexual harassment was not considered as MST. | Stressful Life Events Questionnaire (SLEQ) (Goodman et al., 1998) | Good test-retest reliability (median κ=0.73) and acceptable convergent validity (median κ=0.64) | Dichotomous.  Participants who rated the intensity of “their fear, horror, or helplessness at the time of the stressful event with a score of 8/10 or higher, they were considered to have experienced MST. | 41% |
| (Yalch et al., 2018)  *United States* | Cross-sectional.  Participants were recruited from a VHA hospital and surrounding outpatient clinics as part of a larger study of female veterans' health. Eligible participants (female with an address within VHA catchment area, age between 18 and 70 years, no current psychosis or history of psychosis, and no suicide attempt within the last five years) completed self-report surveys. | MSA | Sexual assault during military service. | Single item asking participants if anyone used “*force, threat of force, or coerce[d] you to have sex against your will”* (Yalch et al., 2018, p29) during military service. | N.R. | Dichotomous | 33% |
| (Zelkowitz et al., 2022)  *United States* | Cross-sectional  Participants were recruited from the VA DoD Identity Repository and completed self-report surveys. Eligible participants were veterans with valid addresses. | MST | Sexual harassment or sexual assault during miliary service. | DRRI-2 (Vogt et al., 2013) | N.R. | Dichotomous | 55.0% |
| (Zerach, 2023)  *Israel* | Cross-sectional  Eligible participants (18 years of age, have served mandatory service in the Israeli Defense Force, and have been released from military service within the last 20 years) completed self-report surveys.  Participants recruited between January and April 2022 via combat veterans' websites and communities, Ariel University, and online adverts and social media campaigns | MST (any), MST-harassment and MST- assault | Sexual harassment and/or sexual assault during military service | VA 2-item MST screening tool | N.R. | Dichotomous (MSH and MSA scored separately) | **MST:**  *Combat veterans:* 30.2%  *Non-combat veterans:* 23.1%  **MST-harassment:**  *Combat veterans:* 29.1%  *Non-combat veterans:* 21.8%  **MST-assault:**  *Combat veterans:* 9.1%  *Non-combat veterans:* 6.4% |
|  |  |  |  |  |  |  |  |

*N.R.= ‘Not Reported’,*

*DoD= Department of Defense, DRRI-1 Deployment Risk and Resilience Inventory-1, DRRI-2 Deployment Risk and Resilience-2, IPV-related MST= Military Sexual Trauma perpetrated by an intimate partner, LEC= Life Events Checklist, MSA= Military Sexual Assault, MSH= Military Sexual Harassment, MST= Military Sexual Trauma, Non-IPV-related MST= MST perpetrated by someone who was not an intimate partner, OEF= Operation Enduring Freedom, OIF= Operation Iraqi Freedom, OND= Operation New Dawn, ODS= Operation Desert Storm, PHQ-9= Patient Health Questionnaire, PTSD= Posttraumatic Stress Disorder, SEQ-DoD= Sexual Experiences Questionnaire- Department of Defense, SES= Sexual Experiences Survey, SHI= Sexual Harassment Inventory, TLEQ= Traumatic Life Events Questionnaire, VHA= Veterans Health Administration, VA= Department of Veterans Affairs,*

**Supplementary Materials Table 3- Supporting Information Related to Mental Health Outcomes Reported in Quantitative Studies**

| Author, Year | Outcome | Measure | Scoring | Cut-off for caseness | Mean score (SD) | | | Rate (%) | | |
| --- | --- | --- | --- | --- | --- | --- | --- | --- | --- | --- |
|  |  |  |  |  | Full sample | MST+ | MST- | Full sample | MST+ | MST- |
| Banducci et al., 2019 | PTSD symptom severity | PCL-M | Likert scale (1-5); higher scores indicate higher symptom severity (range= 17-85) | 50 | 33.82 (18.10) * | N.R. | N.R. | 20.7* | N.R. | N.R. |
|  | Alcohol use severity | CAGE Questionnaire | 4 yes/no questions, totalled and scored continuously (range= 0-4) | 1 | 0.67 (1.11) * | N.R. | N.R. | 32.6* | N.R. | N.R. |
| Blais et al., 2019 | PTSD severity and probable PTSD | PCL-5 | Likert scale (0 – 4); higher scores indicate higher symptom severity (range= 0-80) | ≥ 31 | N.R. | **MSH:** 18.89 (19.77)  **MSA:** 38.22 (23.60) | 13.06 (18.73) | 37.04 | **MSH:** 27.30  **MSA:** 60.18 | 18.26 |
|  | Depression severity and probable depression | Patient Health Questionnaire-8 (PHQ-8) | 4-point frequency scale (0=not at all; 3= nearly every day) summed and continuously scored (range= 0-24) | >9 | N.R. | **MSH:** 8.52 (6.93)  **MSA:** 12.29(7.26) | 7.12 (6.35) | 44.05 | N.R. | N.R. |
|  | Suicidal Ideation | Item 9 of PHQ-9 | Participants reported frequency of suicidal ideation (0= ‘not at all’ – 3= ‘nearly every day’). Scored dichotomously | 1 | N/A | N/A | N/A | 22.10 | **MSH:** 17.46  **MSA:** 33.3 | 12.17 |
| Blais & Geiser | Suicidal Ideation | Item 9 of PHQ-9 | Participants reported frequency of suicidal ideation (0= ‘not at all’ – 3= ‘nearly every day’). Scored on an ordinal scale (higher scores indicate higher suicidal ideation) | 1 | 0.37 (0.79) | N.R. | N.R. | 21.9 | N.R. | N.R. |
| Blais et al., 2023 | PTSD severity and probable PTSD | PCL-5 | Likert scale (1 – 5); higher scores indicate higher symptom severity (range= 0-80) | N. R | N.R. | **MSV Only**= 29.30 (23.75)  **Revictimisation=** 26.29 (22.83) | 12.35 (17.47) | N.R. | N.R. | N.R. |
|  | Depression severity and probable depression | PHQ-8 | 4-point scale (0=not at all; 3= nearly every day) summed and continuously scored (range= 0-24) | N. R | N.R. | **MSV only=** 10.52 (7.50)  **Revictimisation=** 9.95 (7.11) | 7.17 (6.77) | N.R. | N.R. | N.R. |
|  | Suicidal ideation | Item 9 of PHQ-9 | Participants reported frequency of suicidal ideation (0= ‘not at all’ – 3= ‘nearly every day’). Scored on an ordinal scale ((higher scores indicate higher suicidal ideation) | N. R | N.R. | N.R. | N.R. | N.R. | **MSV=** 31.56  **Revictimisation=** 29.58 | 19.05 |
| Breland et al., 2018 | Probable eating disorder | SCOFF Clinical Prediction Guide (Anorexia and Bulimia) and Eating Disorder Examination Questionnaire (EDE-Q) (Binge Eating Disorder) | 5 items scored dichotomously (0=no; 1=yes) and summed (SCOFF); Participants who endorsed excessive concern about shape and/or weight and an average or 1+ bingeing episodes per week (without compensatory behaviours) | N.R. | N.R. | N.R. | N.R. | 15 | N.R. | N.R. |
| Bryan et al., 2015 | Suicidal ideation, | Self-Injurious Thoughts and Behaviors Interview (self-report version) | Items assessing suicidal ideation, suicide planning and suicide attempts; scored dichotomously | N.R. | N/A | N/A | N/A | 30.9 | N.R. | N.R. |
|  | Suicide planning, |  |  | N.R. | N/A | N/A | N/A | 11.4 | N.R. | N.R. |
|  | Suicide attempts |  |  | N.R. | N/A | N/A | N/A | 4.0 | N.R. | N.R. |
| Chang et al., 2001 | General mental health status | SF-36 (mental component summary) | Continuous (range= 0-100); higher scores indicate better mental health | N/A | 35.5 (12.5) | 42.6 (12.6) | N/A | N/A | N/A | N/A |
|  | Depressive symptoms | CES-D | Continuous; higher scores indicate more depressive symptoms | N/A | 10.7 (5.5) | 7.2 (5.3) | N/A | N/A | N/A | N/A |
| Cobb-Scott et al., 2014 | Military-related PTSS | PCL-M | Continuous (range= 17-85); higher scores indicate higher symptom severity | Scores ≥ 50 and endorsing the 3 DSM-IV criteria for PTSD | N.R. | N.R. | N.R. | N.R. | N.R. | N.R. |
| Decker et al., 2021 | Suicidal ideation | Item 9 of the PHQ-9 | Participants reported frequency of suicidal ideation (0= ‘not at all’ – 3= ‘nearly every day’). Coded dichotomously | N. R | N.R. | N.R. | N.R. | 16.4 | 20.6 | 16.1 |
| Dutra et al., 2011 | Past week PTSD symptoms | 4-item Primary Care PTSD Screen | Participants respond to each item dichotomously (yes/no). The number of items endorsed are summed. | 3 | 0.98 (1.20) | N.R. | N.R. | 11.2 | N.R. | N.R. |
|  | Past week depression symptoms | CES-D (2-item abbreviated scale) | Respondents report the frequency of symptoms on a Likert-type scale (1 (rarely/none of the time) – 4 (most of the time)). | N. R | ‘feeling down, depressed, or hopeless’ item score M= 1.72 (SD = 0.74)  anhedonia item score M = 1.76, (SD = 0.85) | N.R. | N.R. | 9.3% reported ‘feeling down, depressed, or hopeless’; 17.4% reported “at least a moderate amount of the time.” | N.R. | N.R. |
| Esopenko et al., 2023 | PTSD symptoms | PCL-C | Likert-type scale; higher scores indicate higher symptom severity (range= 17 – 85) | 37-44 (decided by clinical interviews) | 63.9 (13.8) | **MST-only:** 65.0 (13.2)  **MST+ IPV:** 65.6 (12.8) | 58.7 (12.6) | N.R. | N.R. | N.R. |
|  | Depressive symptoms (in the past 2 weeks) | BDI-II | Participants rate severity of symptoms on a 4-point scale (no symptoms - severe symptom interference) (range= 0 to 63) | >21 | 35.5 (12.2) | **MST-only:** 34.8 (11.8)  **MST+IPV:** 37.2 (12.1) | 30.3 (11.5) | N.R. | N.R. | N.R. |
|  | Suicidality in the week prior to admission | BSSI | Participants rate severity of current suicidal ideation on a 3-point scale (none – greater severity). Responses to 19 items are summed (Range= 1-38) | N.R. | 10.7 (7.6) | **MST-only:** 8.7 (7.3)  **MST+IPV:** 12.2 (7.7) | 9.2 (6.9) | N.R. | N.R. | N.R. |
| Fillo et al., 2023 | Past-year alcohol problems | AUDIT | Participants rate frequency of symptoms on a 4-point scale (Never – Daily or almost daily) and responses are summed (range= 0-40) | N.R. | 3.7 (3.6) | 3.32 (N.R.) | 3.76 (N.R.) | N.R. | N.R. | N.R. |
| Fontana et al., 1998 | Current PTSD Symptom Severity | 17-item PTSD Checklist | Participants rate items (0-5) corresponding to DSM-4 criteria | N.R. | N.R. | N.R. | N.R. | N.R. | N.R. | N.R. |
| Gibson et al., 2019 | PTSD | ICD-9 Codes | Medical diagnoses | N.R. | N.R. | N.R. | N.R. | N. R | 35.3 | 5.6 |
|  | depressive disorders, |  |  |  | N.R. | N.R. | N.R. | N.R. | 56.7 | 30.6 |
|  | Anxiety disorders |  |  |  | N.R. | N.R. | N.R. | N.R. | 35.4 | 18.3 |
|  | Substance Use Disorder |  |  |  | N.R. | N.R. | N.R. | N.R. | 5.4 | 2.0 |
|  | Opioid Use Disorder |  |  |  | N.R. | N.R. | N.R. | N.R. | 1.4 | 0.6 |
|  | Alcohol use disorder |  |  |  | N.R. | N.R. | N.R. | N.R. | 9.0 | 4.0 |
|  | Suicidal Ideation |  |  |  | N.R. | N.R. | N.R. | N.R. | 2.9 | 0.9 |
| Gorman et al., 2022 | PTSD diagnosis and symptoms the last 30 days | SCID-4 | Assessed by doctoral level psychologists |  | N.R. | N.R. | N.R. | **Sexual minority participants:** 60  **Heterosexual participants:** 63 | N.R. | N.R. |
|  |  | 17-item PCL | Participants rate items on a 5-point scale (not at all – extremely). Responses are summed (range= 17- 85), with higher scores indicating greater severity |  |  |  |  |  |  |  |
|  | Major Depressive Disorder diagnosis and symptoms over the last 30 days | SCID-IV | Assessed by doctoral level psychologists |  | N.R. | N.R. | N.R. | **Sexual minority participants:** 28 **Heterosexual participants***:* 26 | N.R. | N.R. |
|  |  | PHQ-9 | Participants rate items on a 4-point scale (not at all – nearly every day). Responses are summed, with higher scores indicating higher severity (range= 0 -27) |  |  |  |  |  |  |  |
| Gradus et al., 2008 | Current depression symptoms | CES-D | N.R. | N.R. | 5.4 (6.0) | N.R. | N.R. | 8 | N.R. | N.R. |
|  | Current problem drinking behaviour | AUDIT | N.R. | >7 | N.R. | N.R. | N. R | 4.1 | N.R. | N.R. |
| Gradus et al., 2017 | Post-military suicidal ideation | SBQ-SF | Dichotomous | Answering ‘yes’ to the suicidal ideation question | N.R. | N.R. | N.R. | 17.4 | N.R. | N.R. |
| Gross et al., 2018 | PTSD symptom severity, | Davidson Trauma Scale | Participants rate frequency (5-point scale; not at all -every day) and severity of symptoms (5-point scale; not at all distressing – extremely distressing) |  | 37.44 (38.98) |  |  | N/A | N/A | N/A |
|  | Lifetime and current PTSD diagnosis | SCID-IV |  |  | N/A | N/A | N/A | **Lifetime:** 42.7%  **Current:** 26.4% | N.R. | N. R |
| Gross et al., 2020 | Past week prenatal depression and symptom severity, | Edinburgh Postnatal Depression Scale (administered verbally at the prenatal interview).  Edinburgh Postnatal Depression Scale (administered verbally at the postdelivery interview) | Participants respond to items on a 3-point scale | N.R. | 6.55 (6.10) | **Harassment-MST:** 8.42 (6.61)  **Assault-MST:** 9.55 (7.10) | **No harassment MST:** 4.55 (4.82)  **No assault MST:** 5.27 (5.12) | N.R. | N.R. | N.R. |
|  | Past week postnatal depression and symptom severity |  |  | N.R. | 5.06 (5.58) | **Harassment MST:** 6.38 (6.22)  **Assault MST**: 7.14 (6.86) | **No harassment MST:** 3.49 (4.25)  **No assault MST:** 4.09 (4.60) | N.R. | N.R. | N.R. |
|  | Pre-natal suicidal ideation | Final item of the Edinburgh Postnatal Depression Scale | Participants respond on a 3-point scale (never – in the past week) | N.R. | N.R. | N.R. | N.R. | 8.1% | N.R. | N.R. |
|  | Post-natal suicidal ideation |  |  | N.R. | N.R. | N.R. | N.R. | 2.7% | N.R. | N.R. |
| Hankin et al., 1999 | Current symptoms (prior month) of depression | Short-form Iowa (1 I-item) version of the CES-D | N.R. | 16 | N.R. | N.R. | N.R. | 40% | 60% | 33% |
|  | Current alcohol use | TWEAK | N.R. | 3 | N.R. | N.R. | N.R. | 5% | 7% | 4% |
| Harned & Fitzgerald, 2002 | Eating disorder symptoms during the past 4 weeks | EDE-Q | Participants rate items on a 5-point scale (never – very often) | N.R. | N.R. | N.R. | N.R. | N.R. | N.R. | N.R. |
| Harned et al., 2002 | Psychological wellbeing (past 4 weeks) | Five items from the Mental Health Index | Participants rate items on a 6-point scale (none of the time - all of the time). Higher total scores indicate higher levels of psychological well-being (Range: 0-25) | N.R. | 17.52 (4.58) | N.R. | N.R. | N.R. | N.R. | N.R. |
| Hendrikx et al., 2023 | Common Mental Health Difficulties | 12-item General Health Questionnaire | N.R. | 4 | N.R. | N.R. | N.R. | N.R. | N.R. | N.R. |
|  | PTSD | PCL-5 | N.R. | 34 | N.R. | N.R. | N.R. | N.R. | N.R. | N.R. |
|  | Harmful alcohol use | 20-item AUDIT | N.R. | 8 | N.R. | N.R. | N.R. | N.R. | N.R. | N.R. |
|  | Somatisation | 15-item Patient Health Questionnaire | N.R. | 15 | N.R. | N.R. | N.R. | N.R. | N.R. | N.R. |
| Himmelfarb et al., 2006 | PTSD presence and severity | PSSI | Determined by interview responses to semi-structured | N.R. | N.R. | N.R. | N.R. | N.R. | 60 | N.R. |
| Hoffmire et al., 2021 | Recent active Suicidal Ideation (past 3 months | C-SSRS | Dichotomous | N.R. | N.R. | N.R. | N.R. | N.R. | N.R. | N. R |
| Kang et al., 2005 | PTSD presence | 17-item PCL | Participants rate 17 items on a 5-point Likert scale | 50 | N.R. | N.R. | N.R. | 15.8 | **Sexual harassment only:** 26.0  **Sexual assault only:** 12.5  **Both sexual harassment and assault:** 49.2 | 11.7 |
| Kearns et al., 2016 | PTSD diagnostic statuses (past 30 days) | PTSD module of the SCID-5 | DSM–5 algorithm | N.R. | N.R. | N. R | N. R | 62.7 | N. R | N.R. |
|  | PTSD symptom severity | PCL-5 | Participants rate items on a 5-point ordinal scale (not at all - extremely), with higher scores indicating higher symptom severity (range= 0-80) | N. R | 39.6 (20.1) | N.R. | N.R. | N.R. | N.R. | N.R. |
|  | MDD diagnostic status (past 30 days) | MDD module of the SCID-5 | Doctoral-level clinician interviewers carried out interviews and employed the DSM–5 algorithm was used to calculate DSM–5 MDD diagnostic status. | N.R. | N.R. | N.R. | N.R. | 26.4 | N. R | N.R. |
|  | MDD Symptom severity | PHQ-9 | Participants rate items on a 4-point scale (not at all – nearly every day), with higher scores indicating higher symptom severity (range= 0-27) | N.R. | 12.7 (6.8) | N.R. | N.R. | N.R. | N.R. | N.R. |
| Kim et al., 2017 | Psychological Distress | Kessler Psychological Distress Scale 10 (K-10) | Participants rate items on a 5-point scale (all of the time – none of the time). Higher total scores indicate higher psychological distress (range= 10-50) | 24 | 15.09 (5.45) | 20.86 (10.38) | 14.81 (6.16) | N.R. | N.R. | N.R. |
| Kimerling et al., 2007 | PTSD | Electronic VHA Medical records | ICD-9 Codes | N.R. | N.R. | N.R. | N.R. | N.R. | N.R. | N.R. |
|  | Depressive disorders |  |  | N.R. | N.R. | N.R. | N.R. | N.R. | N.R. | N.R. |
|  | Anxiety disorders |  |  | N.R. | N.R. | N.R. | N.R. | N.R. | N.R. | N.R. |
|  | Adjustment disorders |  |  | N.R. | N.R. | N.R. | N.R. | N.R. | N.R. | N.R. |
|  | Attention-deficit/ conduct/ disruptive |  |  | N.R. | N.R. | N.R. | N.R. | N.R. | N.R. | N.R. |
|  | Impulse-control disorders |  |  | N.R. | N.R. | N.R. | N.R. | N.R. | N.R. | N.R. |
|  | Eating disorders |  |  | N.R. | N.R. | N.R. | N.R. | N.R. | N.R. | N.R. |
|  | Somatoform disorders |  |  | N.R. | N.R. | N.R. | N.R. | N.R. | N.R. | N.R. |
|  | Alcohol disorders |  |  | N.R. | N.R. | N.R. | N.R. | N.R. | N.R. | N.R. |
|  | Drug abuse |  |  | N.R. | N.R. | N.R. | N.R. | N.R. | N.R. | N.R. |
|  | Suicide and intentional self-inflicted injury |  |  | N.R. | N.R. | N.R. | N.R. | N.R. | N.R. | N.R. |
|  | Bipolar disorders |  |  | N.R. | N.R. | N.R. | N.R. | N.R. | N.R. | N.R. |
|  | Schizophrenia and psychoses |  |  | N.R. | N.R. | N.R. | N.R. | N.R. | N.R. | N.R. |
|  | Dissociative disorders |  |  | N.R. | N.R. | N.R. | N.R. | N.R. | N.R. | N.R. |
| Kimerling et al., 2010 | PTSD | Electronic VHA Medical records | ICD-9 Codes | N/A | N.R. | N.R. | N.R. | N.R. | 51.5% | 21.5% |
|  | Depressive disorders |  |  |  | N.R. | N.R. | N.R. | N.R. | 56.2% | 30.3% |
|  | Anxiety disorders |  |  |  | N.R. | N.R. | N.R. | N.R. | 29.1% | 16.6% |
|  | Alcohol and substance use disorders |  |  |  | N.R. | N.R. | N.R. | N.R. | 13.9% | 5.2% |
|  | Adjustment disorders |  |  |  | N.R. | N.R. | N.R. | N.R. | 20.6 | 13.4 |
| Laws et al., 2016 | PTSD symptoms in the past 30 days | PCL-C | Participants rated items on a 5-item scale (not at all – extremely) (Range =17-85) | N/A | 45.60 (18.1) | N.R. | N.R. | N/A | N/A | N/A |
| Lindsay et al., 2016 | Depressive Disorder | ICD-9 Codes | Derived from the VHA Medical Statistical Analysis System file | N/A | N/A | N/A | N/A | 70.5 | N.R. | N.R. |
|  | Bipolar Disorder |  |  |  | N/A | N/A | N/A | 10.6 | N.R. | N.R. |
|  | Anxiety Disorder |  |  |  | N/A | N/A | N/A | 50.8 | N.R. | N.R. |
|  | Schizophrenia |  |  |  | N/A | N/A | N/A | 5.5 | N.R. | N.R. |
|  | PTSD |  |  |  | N/A | N/A | N/A | 49.2 | N.R. | N.R. |
|  | Alcohol use disorder |  |  |  | N/A | N/A | N/A | 15 | N.R. | N.R. |
|  | Drug use disorder |  |  |  | N/A | N/A | N/A | 16.9 | N.R. | N.R. |
|  | Personality disorder |  |  |  | N/A | N/A | N/A | 15.4 | N.R. | N.R. |
| Luterek et al., 2011 | PTSD | PCL-C | Participants rate items on a 5-point Likert scale (not at all – extremely). Higher total scores indicate higher symptom severity (range= 17-85) | N.R. | N.R. | N.R. | N.R. | N.R. | N.R. | N.R. |
|  | DESNOS | Structured Interview for Disorders of Extreme Stress (SIDES) | Items are rated on a 4-point scale (no problem – extreme problems). Higher total scores indicate higher severity (range= 0 -135) | N.R. | N.R. | N.R. | N.R. | N.R. | N.R. | N.R. |
|  | Depression | PHQ-9 | 9 items assessing symptom severity (range= 0-27), with higher total scores indicating greater depression | N.R. | N.R. | N.R. | N.R. | N.R. | N.R. | N.R. |
| Maguen et al., 2012 | Depression | ICD-9 Diagnoses | Diagnoses associated with VA clinic visits | N.R. | N/A | N/A | N/A | 70 | 75 | 67% |
|  | Anxiety |  |  | N.R. | N/A | N/A | N/A | 39 | 42 | 37% |
|  | adjustment disorders |  |  | N.R. | N/A | N/A | N/A | 29 | 29 | 29% |
|  | alcohol use disorders, |  |  | N.R. | N/A | N/A | N/A | 13 | 16 | 12% |
|  | substance use disorders, |  |  | N.R. | N/A | N/A | N/A | 7 | 9 | 6% |
|  | eating disorders |  |  | N.R. | N/A | N/A | N/A | 2 | 4 | 1% |
| Mahoney et al., 2024 | PTSD symptoms according to DSM-5 clusters (intrusions, and hyperarousal, avoidance, negative alterations in cognitions and mood, hyperarousal) | 17-item PCL | Participants rate the extent to which they were bothered by symptoms on a 5-point scale (not at all – extremely), with higher scores indicating higher severity (range= 17-85). Scores were converted to PTSD Checklist for DSM–5 (PCL-5; Weathers et al., 2013) scores using conversions obtained from a validated crosswalk procedure | 33 | N.R. | N.R. | N.R. | 24.2 | N.R. | N.R. |
| Mercado et al., 2015 | Overall mental health status | Mental Component Scale of the SF-36 | Weighted summary of mental health functioning (weighted to a mean of 50 (SD=10), based on a probability sample of the US population. Higher scores indicate better mental health (range= 0-100) | N.R. | N.R. | **Non- IPV-MST:** 41.7 (95% CI [39.5 -43.9])  **IPV-MST:** 43.2(95% CI [38.0 -48.4]) | 48.2 (95% CI [ 46.2 -50.2]) | N.R. | N.R. | N.R. |
|  | Past week depressive symptoms | CES-D | Participants rate 20 items assessing the frequency of depressive symptoms in the past week on a 4-point scale (“None of the time or less than one day” to “5-7 days”). Higher total scores indicate higher symptoms | N.R. | N.R. | **Non- IPV-MST:** 21.9 (95% CI [19.8 -24.1])  IPV-MST: 19.7 (95% CI [14.4 -25.0]) | 14.5 (95% CI [ 12.6–16.5]) | N.R. | N.R. | N.R. |
|  | Past month PTSD symptoms | 17-item PCL | Participants rate 17 items assessing the extent to which symptoms bothered them in the past month on a 5-point Likert scale (not at all – extremely). Higher total scores indicate higher symptom severity | N.R. | N.R. | Non- IPV-MST: 42.0 (95% CI [39.2 -44.8])  IPV-MST: 41.7(95% CI [35.0 -48.5]) | 29.7 (95% CI [ 27.2–32.3]) | N.R. | N.R. | N.R. |
| Monteith et al., 2018 | Suicidal ideation severity and behaviour in the past 3 months | The C-SSRS interview | Coded as an ordinal variable with 6 levels of suicidal ideation severity (no suicidal ideation- active suicidal ideation with plan and intent) | N.R. | 0.38 (0.97) | N.R. | N.R. | N.R. | N.R. | N.R. |
| Monteith et al., 2023 | Past month suicidal ideation | C-SSRS self-report screener | Coded dichotomously | N.R. | N/A | N/A | N/A | 29.89 | N.R. | N.R. |
|  | post-military suicidal ideation, |  |  | N.R. | N/A | N/A | N/A | 9.88 | N.R. | N.R. |
|  | Post military suicide attempt |  |  | N.R. | N/A | N/A | N/A | 12.32 | N.R. | N.R. |
| Moreau et al., 2022 | Depression symptoms | CESD-10 | Scored dichotomously (presence/ absence of depression symptoms) | 10 | N/A | N/A | N. A | 31.3 | **Repeated sexual comments alone:** 38.6  **MST including 1 form of sexual oppression:** 50.8  **MST including several sexual stressors:** 66.5 | 21.9 |
|  | PTSD symptom severity | PCL-M | 3- level ordinal category variable  based symptom severity PCL-M score (no PTSD, subthreshold PTSD, PTSD positive screen) | PTSD Positive =>50  subthreshold PTSD= 35 - 49 | N/A | N/A | N/A | *Subthreshold PTSD:* 15.3  *PTSD positive:* 4.0 | **Repeated sexual comments alone:** *Subthreshold PTSD:* 24.1  *PTSD positive:* 3.7  **MST including 1 form of sexual oppression:** *Subthreshold PTSD:* 22.2  *PTSD positive:* 7.5  **MST including several sexual stressors:**  *Positive PTSD: 19.1*  *Subthreshold PTSD:* 24.5 | *Subthreshold PTSD:* 10.5  *PTSD positive:* 1.3 |
| Murdoch et al., 2006 | Current PTSD symptom severity | Penn Inventory for PTSD | Higher scores indicate higher severity (range= 0-78) | >35 | N. R | **SHI range 0-6.26:** 33.4 (1.2)  **SHI Range 6.29-13.43:**33.6 (1.2)  **SHI Range 13.47-20.01:** 34.8 (1.3)  **SHI range 20.04-29.32:** 38.4 (1.3)  **SHI range 29.34-39.52:** 40.8 (1.3) | N.R. | N.R. | N.R. | N.R. |
| Murdoch et al., 2010 | PTSD | Penn Inventory for PTSD | Higher scores indicate higher severity | ≥35 | 15.5 (10.5) * | **Sexual harassment or sexual identity challenges:** 17.8 (1.0) *  **Both sexual harassment and sexual identity challenges:** 21.9 (11.5) *  **Sexual harassment, sexual identity challenges and sexual assault:** 23.7 (12.1) ***** | 14.9 (9.4) * | 7.4 *** | N.R. | N. R |
|  | Depression | 5-item RAND Mental Health Battery | Higher scores indicate higher severity | ≥17 | 10.6 (3.7) * | **Sexual harassment or sexual identity challenges:** 10.7 (3.5) *  **Both sexual harassment and sexual identity challenges:** 11.9 (3.8) *  **Sexual harassment, sexual identity challenges and sexual assault**: 12.5 (4.0) *** | 9.8 (3.5) * | 8.6 *** | N. R | N.R. |
|  | Anxiety | 3-item PRIME-MD screeners | N.R. | N.R. | 1.0 (1.0) * | **Sexual harassment or sexual identity challenges:** 0.9 (0.9) *  **Both sexual harassment and sexual identity challenges:** 1.2 (1.0) *  **Sexual harassment, sexual identity challenges and sexual assault**:1.5 (1.0) * | 0.8 (0.9) * | ~59%*** | N.R. | N.R. |
|  | Somatisation | 16-item PRIME-MD screeners | N.R. | N.R. | 3.9 (3.4) * | **Sexual harassment or sexual identity challenges:** 3.8 (3.2) *  **Both sexual harassment and sexual identity challenges:** 5.3 (3.8) *  **Sexual harassment, sexual identity challenges and sexual assault**:6.4 (3.9) * | 3.0 (3.0) * | 45* | N.R. | N.R. |
| Murray-Swank et al., 2018 | Psychological distress | Brief Symptom Inventory-18 (BSI-18) | N. R | 63 | 18.87 (15.32) | 21.21 (15.61) | 12.50 (9.87) | 35 | N. R | N.R. |
|  | PTSD | PCL-5 | N. R | 33 | 29.07 (18.95) | 33.08 (18.35) | 19.47 (15.51) | 40 | N.R. | N. R |
|  | Suicide risk | SBQ-R | N.R. | 7 | 5.97 (3.39) | 6.62 (3.74) | 4.63 (2.03) | 36 | N.R. | N.R. |
| Newins et al., 2021 | PTSD Symptom Severity | Davidson Trauma Scale | Participants rate the frequency and severity of PTSD symptoms on a 5-point scale, with higher total scores indicating greater symptom severity (range= 0-136) | N.R. | N.R. | 62.74 (39.89) | **No adulthood sexual assault:** 35.88 (39.49)  **Civilian Sexual Assault only:** 41.89 (37.24) | N.R. | N.R. | N. R |
|  | Depression symptoms | BDI-II | Participants rate items on a 4 points scale, with higher total scores greater depression symptoms (range= 0-63) | N.R. | N.R. | 24.79 (13.85) | **No adulthood sexual assault:** 13.82 (12.94)  **Civilian Sexual Assault only:** 16.34 (12.72) | N.R. | N.R. | N. R |
|  | Past week suicidal ideation | BSSI | Participants rate items on a 3-point scale, with higher total scores indicating greater suicidal ideation (range= 0-63) | 3 | N.R. | 1.16 (2.52) | **Civilian Sexual Assault only:** 0.48 (1.32)  **No adulthood sexual assault:** 0.77 (2.65) | N.R. | 15.9% | **Civilian Sexual Assault only**: 6.8  **No adulthood sexual assault:** 7.6 |
|  | Alcohol misuse | AUDIT | N.R. | N.R. | N.R. | 4.21 (6.26) | **Civilian Sexual Assault only:** 4.44 (5.48)  **No adulthood sexual assault:** 2.50 (3.79) | N.R. | N.R. | N. R |
|  | Drug use in last 12 months | DAST-20 | Participants respond dichotomously to items (Yes/No) (range = 0-20) | N.R. | N.R. | 1.16 (2.52) | **No adulthood sexual assault:** 0.51 (1.77)  **Civilian Sexual Assault only:** 0.77 (2.62) | N.R. | N.R. | N.R. |
| Rønning et al., 2024 | Past week posttraumatic stress | 10-item Posttraumatic Symptom Scale | Participants rate items on a 7-point Likert scale (never/ rarely- very often) (range = 10-70) | N.R. | 16.9 (8.4) | N.R. | N.R. | N.R. | N.R. | N.R. |
|  | Current anxiety symptoms | 14-item Hospital Anxiety and Depression Scale | Participants rate items on a 3-point scale, with higher total scores indicating higher symptom severity (range= 0-21) | N.R. | 3.6 (3.2) | N.R. | N.R. | N.R. | N.R. | N.R. |
|  | Current depression symptoms |  |  | N.R. | 2.1 (2.9) | N.R. | N.R. | N.R. | N.R. | N.R. |
| Sandhu et al., 2022 | Disordered eating | EAT-26 | Participants rate items from 3 subscales (Dieting, Bulimia and Food Preoccupation & Oral Control) on a 6-point Likert scale (never- always). Higher sum of total scores from the 3 subscales indicates higher disordered eating symptoms | 20 | N.R. | 10.92 (11.42) | N.R. | 15.3% | N.R. | N.R. |
| Skinner et al., 2000 | Mental health | SF-36 (mental health subscale) | Higher total scores indicate better mental health (range= 0-100) | N.R. | N.R. | N.R. | N.R. | N.R. | **Sexual harassment:** 59.5  **Sexual assault:** 53.7 | **No sexual harassment:** 65.3  **No Sexual assault:** 64.6 |
|  | Depression | CES-D | N.R. | N.R. | N.R. | N.R. | N.R. | N.R. | **Sexual harassment:** 46.3  **Sexual assault:** 60.2 | **No sexual harassment:** 29.7  **No sexual assault:** 32.4 |
| Smith et al., 2017 | Current PTSD symptom severity | PCL-M | Participants were asked to think about events which were most disturbing during deployment and rate the extent to which symptom bothered them on a 5-point scale (not at all – extremely) (range = 17-85) | N.R. | 34.56 (17.34) | N.R. | N.R. | N.R. | N.R. | N.R. |
| Smith et al., 2020 | PTSD Symptoms | 4-item PTSD screener (measured at 3 time points) | Participants were asked to rate items on a 4-point scale e (not at all – extremely) based on the following: “Below is a list of reactions that Veterans have in response to stressful military experiences. Please read each one carefully and select how much you have been bothered by that problem in the past 30 days” | N.R. | **Time 1:** 1.89 (1.08)  **Time 2:** 1.93 (1.04)  **Time 2:** 1.74 (0.95) | N.R. | N.R. | N.R. | N.R. | N.R. |
| Stefanovics et al., 2023 | Past year suicidal ideation | Question 2 of the SBQ-R | Dichotomous | Positive endorsement | N.R. | N.R. | N.R. | 18.1 | N.R. | N.R. |
| Street et al., 2008 | Current symptoms of depression, | CES-D | Participants rated the frequency with which they experienced symptoms on a 4-point scale (none of the time to less than 1 day - 5 to 7 days). Item responses were summed to provide a total scale score | 10 | N.R. | N.R. | N.R. | N.R. | **Sexual harassment only:** 20.7  **Sexual harassment and sexual assault:** 39.3 | 13.2 |
|  | Current (past month) and lifetime PTSD | 17-item PCL (in reference to sexual harassment during military service) | Participants rated item on a 5-point Likert scale ranging (not at all to (extremely). Items were summed to create a total scale score. | 44 | N.R. | N.R. | N.R. | N.R. | **Sexual harassment only:**  *Lifetime PTSD:* 8.1  *Current PTSD:* 2.1  **Sexual harassment and sexual assault:**  *Lifetime PTSD:* 37.9  *Current PTSD:* 12.1 | N/A |
|  | Past 6-month somatic symptoms | Participants were asked about 4 symptom categories corresponding to somatization disorder in the DSM-IV edition Text Revision (pain, gastrointestinal, psychoneurological and sexual) and about menstrual symptoms, | Participants rated each item on a 4-point Likert scale (not at all – extremely). Item responses were summed to create a total score. | Participants were classified as high and low somatization based on a median split (median = 4.7) | N.R. | N.R. | N.R. | N.R. | **Sexual harassment only:** 53.2  **Sexual harassment and sexual assault:** 69.1 | 39.5 |
| Sumner et al., 2021 | PTSD | ICD-9 and ICD-10 codes from VHA inpatient, outpatient, and purchased care data | Dichotomous | N/A | N/A | N/A | N/A | 22.3 | 48.5 | 13.0 |
|  | Depression |  |  |  | N/A | N/A | N/A | 27.1 | 44.1 | 21.1 |
|  | Bipolar Disorder |  |  |  | N/A | N/A | N/A | 3.3 | 6.7 | 2.1 |
|  | Anxiety |  |  |  | N/A | N/A | N/A | 21.4 | 33.0 | 17.4 |
|  | Suicidal Ideation |  |  |  | N/A | N/A | N/A | 4.3 | 9.3 | 2.6 |
|  | Alcohol Dependence |  |  |  | N/A | N/A | N/A | 4.4 | 9.0 | 2.8 |
|  | Substance Dependence |  |  |  | N/A | N/A | N/A | 3.5 | 6.9 | 2.2 |
| Surís et al., 2004 | PTSD | Clinician Administered PTSD Scale (CAPS) | Dichotomous | N.R. | N.R. | N.R. | N.R. | N.R. | 41.6 | 10.4 |
| Surís et al., 2007 | Psychological symptom patterns | Brief Symptom Inventory (BSI) | N.R. | N.R. | N.R. | 46.05 |  | N/A | N/A | **Civilian sexual assault only:** 42.24  **No sexual assault:** 7.40 |
|  | Depression | CES-D Short Form–Iowa | N.R. | N.R. | N.R. | 9.9 (3.2) | **Civilian sexual assault only:** 8.9 (3.1)  **No sexual assault:** 8.1 (3.3) | N.R. | 52.5 | **Civilian sexual assault only:** 37.5  **No sexual assault:** 34.4 |
|  | Harmful alcohol use | TWEAK | N.R. | N.R. | N.R. | 0.6 (1.4) | **Civilian sexual assault only:** 0.5 (1.3)  **No sexual assault:** 0.2 (0.9) | N.R. | 14.0 | **Civilian sexual assault only:** 6.0  **No sexual assault:** 5.5 |
|  | Mental health | SF-36 | N.R. | N.R. | N.R. | 62.23 | **Civilian sexual assault only:** 58.62  **No sexual assault:** 67.19 | N/A | N/A | N/A |
| Weberman et al., 2023 | Past month PTSD symptom severity (measured at T2) | PCL-C | Participants rate items on a 5-point scale (not at all – extremely). Item responses are summed to create a total scale score (range= 17-85) | 44 | 37.48 (17.69) | N.R. | N.R. | N.R. | N.R. | N.R. |
| Wilson et al., 2020 | Self-directed violence | BSSI | Dichotomous | Self-report of attempting suicide | N/A | N/A | N/A | 10.6 | N.R. | N.R. |
| Wolfe et al., 1998 | PTSD Symptomology | Mississippi Scale for Combat-Related PTSD | Participants respond to 35 items on a 5-point Likert scale (higher scores indicate higher symptomology) | N.R. | N.R. | **Sexual assault:** 91.83 (22.69)  **Physical sexual harassment:**77.79 (24.41)  **Verbal sexual harassment:** 73.39 (16.17) | 71.36 (17.53) | N.R. | N.R. | N.R. |
| Yaeger et al., 2006 | Current PTSD | PSSI | Participant responses to 17 semi-structured interview items are summed to calculate a total PTSD severity score | N.R. | N.R. | N.R. | N.R. | **Participants reporting any kind of trauma exposure:** 43 | **Participants reporting MST and other trauma exposure:** 60  **Participants reporting MST only:** 25 | **Participants reporting trauma exposure which was not MST:** 29 |
| Yalch et al., 2018 | Hazardous alcohol use, | AUDIT | Participants respond to 10 items on a 3–5-point Likert-type scale assessing frequency and number of drinks | 8 | N.R. | N.R. | N.R. | 7 | N.R. | N.R. |
|  | hazardous drug use over the past 12 months | DAST-10 | Participants respond dichotomously to items (true/false) | 3 | N.R. | N.R. | N.R. | 13 | N.R. | N.R. |
|  | PTS symptomology | PCL-5 | Participants rate the extent to which each symptom bothered them over the past month using a 5-point scale (not at all – extremely) | 33 | N.R. | N.R. | N.R. | 30 | N.R. | N.R. |
| Zelkowitz et al., 2022 | Disordered eating symptoms | EDDS-5 | Participants responded to 17 items. Responses were summed to provide a total disordered eating score. | N.R. | 16.97 (18.07) | N.R. | N.R. | 18.3 | N.R. | N.R. |
|  | PTSD | PCL-5 | Participants rate the extent to which PTSD bother them on a 5-point scale (not at all – extremely). Higher total scores indicate higher symptom severity (range= 0-80) | 33 | 21.23 (20.18) | N.R. | N.R. | 25.1 | N.R. | N.R. |
| Zerach et al., 2023 | PTSD | ITQ | Participants rate the severity of symptoms on items 1-6 (range = 0-24) | Endorsement of 1/2 symptoms from each PTSD symptom cluster and an additional functional impairment | 7.29 (6.54) | **MST-harassment:** 8.84 (6.54)  **MST-assault:** 11.90 (7.00) | 6.61 (6.43) | 6.1 | N.R. | N.R. |
|  | CPTSD |  | Participants rate the severity of symptoms on items 7-12 (range= 0-24) | Endorsement a severity rating of 2 or higher for at least 1 out of 2 symptoms from each PTSD symptom cluster, additional functional impairment, and the additional CPTSD symptom clusters | 7.46 (5.78) | **MST-harassment:** 8.17 (5.86)  **MST-assault:**  9.65 (6.43) | 7.15 (5.73) | 8.7 | N.R. | N.R. |

**indicates values not stratified by sex/ gender in studies with mixed samples.*

*N.R.= ‘Not Reported’; N/A= ‘Not Applicable’*

*AUDIT= Alcohol Use Disorders Identification Test,* BDI-II*= Beck Depression Inventory- 2, BSSI= Beck Scale for Suicide Ideation, CES-D= Center for Epidemiologic Studies Depression, CPTSD= Complex Posttraumatic Stress Disorder, C-SSRS= Columbia Suicide Severity Rating Scale, DAST-10= Drug Abuse Screening Test (10 item version, DAST-20= Drug Abuse Screening Test (20 item version), DSM-IV= Diagnostic and Statistical Manual of Mental Disorders, Fourth Edition, DSM-V= Diagnostic and Statistical Manual of Mental Disorders, Fifth Edition, EAT-26= Eating Attitudes Test-26, , EDE-Q= Eating Disorder Examination Questionnaire , EDDS-5= Eating Disorder Diagnostic Scale, ICD-9= International Classification of Diseases, Ninth Revision ICD-10= International Classification of Diseases, 10th Revision IPV-MST = Military Sexual Trauma perpetrated by an intimate partner, ITQ-= International Trauma Questionnaire, MDD= Major Depressive Disorder, MSA= Military Sexual Assault, MSH= Military Sexual Harassment, MST= Military Sexual Trauma , MST+= Participants who endorsed/ experienced MST, MST-= Participants who endorsed/ experienced MST, Non-IPV-MST= MST perpetrated by someone who was not an intimate partner, PCL-5= PTSD Checklist for DSM-5, PCL-C= PTSD Checklist-Civilian PCL-M= PSTD Checklist, Military Version, PHQ-8= Patient Health Questionnaire (8-items), PHQ-9= Patient Health Questionnaire (9-items), PRIME-MD= Primary Care Evaluation of Mental Disorders, PSSI= PTSD Symptom Scale-Interview, SBQ-SF= 4-item Suicidal Behaviors Questionnaire-Short Form, SD= Standard Deviation SCID-4=* Structured Clinical Interview for DSM-IV, *SCID-5= Structured Clinical Interview for DSM-V, SF-36= Medical Outcomes Study Short Form, SHI= Sexual Harassment Inventory, VHA= Veterans Health Administration*

**References**

Banducci, A., McCaughey, V., Gradus, J., & Street, A. (2019). The associations between deployment experiences, PTSD, and alcohol use among male and female veterans. *Addictive Behaviors*, *98*. <https://doi.org/https://doi.org/10.1016/j.addbeh.2019.106032>

Blais, R., Brignone, E., Fargo, J., Livingston, W., & Andresen, F. (2019). The importance of distinguishing between harassment-only and assault military sexual trauma during screening. *Military Psychology*, *31*(3), 227-232. <https://doi.org/https://doi.org/10.1080/08995605.2019.1598218>

Blais, R., & Geiser, C. (2019). Depression and PTSD-related anhedonia mediate the association of military sexual trauma and suicidal ideation in female service members/veterans. *Psychiatry Research*, *279*, 148-154. <https://doi.org/https://doi.org/10.1016/j.psychres.2018.12.148>

Blais, R., Livingston, W., Barrett, T., & Tannahill, H. (2023). Sexual violence in military service members/veterans individual and interpersonal outcomes associated with single and multiple exposures to civilian and military sexual violence. *JOURNAL OF INTERPERSONAL VIOLENCE*, Article 08862605221101197. <https://doi.org/10.1177/08862605221101197>

Breland, J. Y., Donalson, R., Yongmei, L., Hebenstreit, C. L., Goldstein, L. A., & Maguen, S. (2018). Military sexual trauma is associated with eating disorders, while combat exposure is not. *Psychological Trauma: Theory, Research, Practice & Policy*, *10*(3), 276-281. <https://doi.org/10.1037/tra0000276>

Bryan, C. J., Bryan, A. O., & Clemans, T. A. (2015). The association of military and premilitary sexual trauma with risk for suicide ideation, plans, and attempts. *Psychiatry Research*, *227*(2-3), 246-252. <https://doi.org/doi.org/10.1016/j.psychres.2015.01.030>

Chang, B.-H., Skinner, K. M., & Boehmer, U. (2001). Religion and mental health among women veterans with sexual assault experience [Behavior Disorders & Antisocial Behavior 3230

Military Psychology 3800]. *Special Issue: Biopsychosocial aspects of patient care*, *31*(1), 77-95. <https://doi.org/https://dx.doi.org/10.2190/0NQA-YAJ9-W0AM-YB3P>

Cobb Scott, J., Pietrzak, R. H., Southwick, S. M., Jordan, J. B., Silliker, N., Brandt, C. A., & Haskell, S. G. (2014). Military sexual trauma interacts with combat exposure to increase risk for posttraumatic stress symptomatology in female Iraq and Afghanistan veterans. *Journal of Clinical Psychiatry*, *75*(6), 637-643. <https://doi.org/https://doi.org/10.4088/JCP.13m08808>

Decker, S. E., Ramsey, C. M., Ronzitti, S., Kerns, R. D., Driscoll, M. A., Dziura, J., Skanderson, M., Bathulapalli, H., Brandt, C. A., Haskell, S. G., & Goulet, J. L. (2021). Military sexual trauma and suicidal ideation in VHA-care-seeking OEF/OIF/OND veterans without mental health diagnosis or treatment. *Psychiatry Research*, *303*. <https://doi.org/> <https://doi.org/10.1016/j.psychres.2021.114089>

Dutra, L., Grubbs, K. M., Greene, C. J., Trego, L. L., McCartin, T. L., Kloezeman, K., & Morland, L. A. (2011). Women at war: implications for mental health. *Journal of Trauma and Dissociation*, *12*(1), 25-37. <https://doi.org/https://doi.org/10.1080/15299732.2010.496141>

Esopenko, C., De Souza, N., Wilde, E. A., Dams-O'Connor, K., Teng, E. L., & Menefee, D. S. (2023). Characterizing the influence of exposure to military sexual trauma and intimate partner violence on mental health outcomes among female veterans. *JOURNAL OF INTERPERSONAL VIOLENCE*. <https://doi.org/10.1177/08862605231156193>

Fillo, J., Goodell, E. A. M., Homish, D. L., & Homish, G. G. (2023). Sex differences in the relation between military sexual trauma and risk for alcohol misuse among US Army Reserve and National Guard soldiers. *ALCOHOL-CLINICAL AND EXPERIMENTAL RESEARCH*. <https://doi.org/10.1111/acer.15045>

Fitzgerald, L. F., Magley, V. J., Drasgow, F., & Waldo, C. R. (1999). Measuring Sexual Harassment in the Military: The Sexual Experiences Questionnaire (SEQ—DoD). *Military Psychology*, *11*(3), 243-263. <https://doi.org/10.1207/s15327876mp1103_3>

Fontana, A., & Rosenheck, R. A. (1998). Duty-related and sexual stress in the etiology of PTSD among women veterans who seek treatment. *Psychiatric Services*, *49*(5), 658-662. <https://www.proquest.com/scholarly-journals/duty-related-sexual-stress-etiology-ptsd-among/docview/42414898/se-2?accountid=28179>

<http://www.ptsd.va.gov/professional/articles/article-pdf/id20021.pdf>

Gibson, C. J., Maguen, S., Xia, F., Barnes, D. E., Peltz, C. B., & Yaffe, K. (2019). Military sexual trauma in older women veterans: Prevalence and Comorbidities. *Journal of general internal medicine*. <https://doi.org/https://dx.doi.org/10.1007/s11606-019-05342-7>

Goodman, L. A., Corcoran, C., Turner, K., Yuan, N., & Green, B. L. (1998). Assessing traumatic event exposure: General issues and preliminary findings for the Stressful Life Events Screening Questionnaire. *Journal of Traumatic Stress*, *11*(3), 521-542. <https://doi.org/https://doi.org/10.1023/A:1024456713321>

Gorman, K., Kearns, J., Pantalone, D., Bovin, M., Keane, T., & Marx, B. (2021). The impact of deployment-related stressors on the development of PTSD and depression among sexual minority and heterosexual female veterans. *Psychological Trauma: Theory, Research, Practice, and Policy*. <https://doi.org/https://doi.org/10.1037/tra0001102>

Gradus, J. L., King, M. W., Galatzer‐Levy, I., & Street, A. E. (2017). Gender differences in machine learning models of trauma and suicidal ideation in veterans of the Iraq and Afghanistan wars. *Journal of Traumatic Stress*, *30*(4), 362-371. <https://doi.org/10.1002/jts.22210>

Gradus, J. L., Street, A. E., Kelly, K. A., & Stafford, J. A. (2008). Sexual harassment experiences and harmful alcohol use in a military sample: differences in gender and the mediating role of depression. *Journal of Studies on Alcohol and Drugs*, *69*(3), 348-351. <https://www.proquest.com/scholarly-journals/sexual-harassment-experiences-harmful-alcohol-use/docview/42447552/se-2?accountid=28179>

<http://www.ptsd.va.gov/professional/articles/article-pdf/id32354.pdf>

Gray, M. J., Litz, B. T., Hsu, J. L., & Lombardo, T. W. (2004). Psychometric Properties of the Life Events Checklist. *Assessment*, *11*(4), 330-341. <https://doi.org/10.1177/1073191104269954>

Gross, G. M., Cunningham, K. C., Moore, D. A., Naylor, J. C., Brancu, M., Wagner, H. R., Elbogen, E. B., Calhoun, P. S., & Kimbrel, N. A. (2018). Does deployment-related military sexual assault interact with combat exposure to predict posttraumatic stress disorder in female veterans? *Traumatology*, *May 2018*. <http://ovidsp.ovid.com/ovidweb.cgi?T=JS&PAGE=reference&D=pmnm4&NEWS=N&AN=30202245>

Gross, G. M., Ronzitti, S., Combellick, J. L., Decker, S. E., Mattocks, K. M., Hoff, R. A., Haskell, S. G., Brandt, C. A., & Goulet, J. L. (2020). Differences in military sexual trauma and severe self-directed violence. *American jounal of preventative medicine 58*(5), 675-682. <https://doi.org/10.1016/j.amepre.2019.12.006>

Hankin, C. S., Skinner, K. M., Sullivan, L. M., Miller, D. R., Frayne, S. M., & Tripp, T. J. (1999). Prevalence of depressive and alcohol abuse symptoms among women VA outpatients who report experiencing sexual assault while in the military. *Journal of Traumatic Stress*, *12*(4), 601-612. <https://doi.org/https://doi.org/10.1023/A:1024760900213>

Harned, M. S., & Fitzgerald, L. F. (2002). Understanding the link between sexual harassment and eating disorder symptoms: a mediational analysis. *Journal of Consulting and Clinical Psychology*, *70*(5), 1170-1181. <https://doi.org/https://doi.org/10.1037//0022-006X.70.5.1170>

Harned, M. S., Ormerod, A. J., Palmieri, P. A., Collinsworth, L. L., & Reed, M. E. (2002). Sexual assault and other types of sexual harassment by workplace personnel: a comparison of antecedents and consequences. *Journal of Occupational Health Psychology*, *7*(2), 174-188. <https://doi.org/https://doi.org/10.1037//1076-8998.7.2.174>

Hendrikx, L. J., Williamson, V., & Murphy, D. (2023). Adversity during military service: the impact of military sexual trauma, emotional bullying and physical assault on the mental health and wellbeing of women veterans. *BMJ Military Health 169*(5), 419-424. <https://doi.org/10.1136/bmjmilitary-2021-001948>

Himmelfarb, N., Yaeger, D., & Mintz, J. (2006). Posttraumatic stress disorder in female veterans with military and civilian sexual trauma. *Journal of Traumatic Stress: Official Publication of The International Society for Traumatic Stress Studies*, *19*(6), 837-846. <https://doi.org/doi.org/10.1002/jts.20163>

Hoffmire, C., Monteith, L., Denneson, L., Holliday, R., Park, C., Mazure, C., & Hoff, R. (2021). A sex-stratified analysis of suicidal ideation correlates among deployed post-9/11 veterans: results from the Survey of Experiences of Returning Veterans. *Journal of Affective Disorders*, *294*, 824-830. <https://doi.org/https://doi.org/10.1016/j.jad.2021.07.015>

Kang, H. K., Dalager, N. A., Mahan, C. M., & Ishii, E. K. (2005). The role of sexual assault on the risk of PTSD among Gulf War veterans. *Annals of Epidemiology*, *15*(3), 191-195. <https://doi.org/https://doi.org/10.1016/j.annepidem.2004.05.009>

Kearns, J. C., Gorman, K. R., Bovin, M. J., Green, J. D., Rosen, R. C., Keane, T. M., & Marx, B. P. (2016). The effect of military sexual assault, combat exposure, postbattle experiences, and general harassment on the development of PTSD and MDD in Female OEF/OIF veterans. *Translational Issues in Psychological Science*, *2*(4), 418-428. <https://doi.org/doi.org/10.1037/tps0000098>

Kim, T. K., Lee, H. C., Lee, S. G., Han, K. T., & Park, E. C. (2017). The influence of sexual harassment on mental health among female military personnel of the Republic of Korea Armed Forces. *Journal of the Royal Army Medical Corps*, *163*(2), 104-110. <https://doi.org/doi.org/10.1136/jramc-2015-000613>

Kimerling, R., Gima, K., Smith, M. W., Street, A., & Frayne, S. (2007). The Veterans Health Administration and military sexual trauma. *American Journal of Public Health*, *97*(12), 2160-2166. <https://doi.org/doi.org/10.2105/AJPH.2006.092999>

Kimerling, R. E., Street, A. E., Pavao, J. R., Smith, M. W., Cronkite, R. C., Holmes, T. H., & Frayne, S. M. (2010). Military-related sexual trauma among Veterans Health Administration patients returning from Afghanistan and Iraq. *American Journal of Public Health*, *100*(8), 1409-1412. <https://doi.org/https://doi.org/10.2105/AJPH.2009.171793>

King, D. W., King, L. A., & Vogt, D. S. (2003). *Manual for the deployment risk and resilience inventory (DRRI): A collection of measures for studying deployment-related experiences of military veterans.* National Center for PTSD.

Kubany, E. S., Leisen, M. B., Kaplan, A. S., Watson, S. B., Haynes, S. N., Owens, J. A., & Burns, K. (2000). Development and preliminary validation of a brief broad-spectrum measure of trauma exposure: The Traumatic Life Events Questionnaire. *Psychological Assessment*, *12*(2), 210-224. <https://doi.org/10.1037/1040-3590.12.2.210>

Laws, H., Mazure, C. M., McKee, S. A., Park, C. L., & Hoff, R. A. (2016). Within-unit relationship quality mediates the association between military sexual trauma and posttraumatic stress symptoms in veterans separating from military service. *Psychological Trauma: Theory, Research, Practice, and Policy*, *8*(5), 649-656. <https://doi.org/https://doi.org/10.1037/tra0000118>

Lindsay, J. A., Keo‐Meier, C., Hudson, S., Walder, A., Martin, L. A., & Kauth, M. R. (2016). Mental health of transgender veterans of the Iraq and Afghanistan conflicts who experienced military sexual trauma. *Journal of Traumatic Stress*, *29*(6), 563-567. <https://doi.org/10.1002/jts.22146>

Luterek, J. A., Bittinger, J. N., & Simpson, T. L. (2011). Posttraumatic sequelae associated with military sexual trauma in female veterans enrolled in VA outpatient mental health clinics. *Journal of Trauma & Dissociation*, *12*(3), 261-274. <https://doi.org/10.1080/15299732.2011.551504>

Maguen, S., Cohen, B. E., Ren, L., Bosch, J. O., Kimerling, R. E., & Seal, K. H. (2012). Gender differences in military sexual trauma and mental health diagnoses among Iraq and Afghanistan veterans with posttraumatic stress disorder. *Women's Health Issues*, *22*(1), e61-e66. <https://doi.org/https://doi.org/10.1016/j.whi.2011.07.010>

Mahoney, C. T., Shayani, D. R., & Iverson, K. M. (2024). Differential indirect effects of military sexual trauma on posttraumatic stress disorder symptom clusters via past-year intimate partner violence experiences. *Traumatology*, *30*(1). <https://doi.org/doi.org/10.1037/trm0000242>

Mercado, R., Ming Foynes, M., Carpenter, S. L., & Iverson, K. M. (2015). Sexual intimate partner violence as a form of MST: An initial investigation [Behavior Disorders & Antisocial Behavior 3230

Military Psychology 3800]. *Special Issue: Military Sexual Trauma*, *12*(4), 348-356. <https://doi.org/https://dx.doi.org/10.1037/ser0000056>

Monteith, L. L., Hoffmire, C. A., Holliday, R., Park, C. L., Mazure, C. M., & Hoff, R. A. (2018). Do unit and post-deployment social support influence the association between deployment sexual trauma and suicidal ideation? *Psychiatry Research*, *270*, 673-681. <https://doi.org/doi.org/10.1016/j.psychres.2018.10.055>

Monteith, L. L., Kittel, J. A., Schneider, A. L., Miller, C. N., Holliday, R., Katon, J. G., Brenner, L. A., & Hoffmire, C. A. (2023). Military sexual trauma among women veterans using Veterans Health Administration reproductive health care: screening challenges and associations with post-military suicidal ideation and suicide attempts. *JOURNAL OF INTERPERSONAL VIOLENCE*, *38*(11-12), 7578-7601. <https://doi.org/doi.org/10.1177/08862605221145725>

Moreau, C., Duron, S., Bedretdinova, D., Bohet, A., Panjo, H., Bajos, N., & Meynard, J. B. (2022). Mental health consequences of military sexual trauma: Results from a national survey in the French military. *BMC public health*, *22*(1). <https://doi.org/10.1186/s12889-022-12545-x>.

Murdoch, M., & McGovern, P. G. (1998). Measuring sexual harassment: Development and validation of the Sexual Harassment Inventory. *Violence and Victims*, *13*(3), 203-216.

Murdoch, M., Polusny, M. A., Hodges, J., Cowper, D., Murdoch, M., Polusny, M. A., Hodges, J., & Cowper, D. (2006). The association between in-service sexual harassment and post-traumatic stress disorder among Department of Veterans Affairs disability applicants. *Military Medicine*, *171*(2), 166-173. <https://doi.org/10.7205/milmed.171.2.166>

Murdoch, M., Pryor, J. B., Polusny, M. A., Wall, M. M., Cowper Ripley, D. C., & Gackstetter, G. D. (2010). The association between military sexual stress and psychiatric symptoms after controlling for other stressors. *Journal of Psychiatric Research*, *44*(16), 1129-1136. <https://doi.org/https://doi.org/10.1016/j.jpsychires.2010.09.009>

Murray-Swank, A., N., Dausch, B. M., & Ehrnstrom, C. (2018). The mental health status and barriers to seeking care in rural women veterans [Military Psychology 3800]. *Journal of Rural Mental Health*, *42*(2), 102-115. <https://doi.org/https://dx.doi.org/10.1037/rmh0000095>

Newins, A. R., Glenn, J. J., Wilson, L. C., Wilson, S. M., Kimbrel, N. A., Beckham, J. C., Workgroup, V. A. M.-A. M., & Calhoun, P. S. (2021). Psychological outcomes following sexual assault: Differences by sexual assault setting [Criminal Behavior & Juvenile Delinquency 3236

Military Psychology 3800]. *Psychological Services*, *18*(4), 504-511. <https://doi.org/https://dx.doi.org/10.1037/ser0000426>

Rønning, L., Shor, R., Anyan, F., Hjemdal, O., Jakob Bøe, H., Dempsey, C. L., & Espetvedt Nordstrand, A. (2024). The prevalence of sexual harassment and bullying among Norwegian Afghanistan veterans: Does workplace harassment disproportionately impact the mental health and life satisfaction of female soldiers? *JOURNAL OF INTERPERSONAL VIOLENCE*. <https://doi.org/10.1177/08862605241248432>.

Sandhu, D., Dougherty, E., & Haedt-Matt, A. (2022). PTSD symptoms as a potential mediator of associations between military sexual assault and disordered eating. *Eating Disorders*. <https://doi.org/https://doi.org/10.1080/10640266.2022.2133586>

Skinner, K. M., Kressin, N. R., Frayne, S. M., Tripp, T. J., Hankin, C. S., Miller, D. R., & Sullivan, L. M. (2000). The prevalence of military sexual assault among female Veterans' Administration outpatients. *JOURNAL OF INTERPERSONAL VIOLENCE*, *15*(2), 291-310. <https://doi.org/https://doi.org/10.1177/088626000015003005>

Smith, B. N., Wang, J. M., Vaughn-Coaxum, R. A., Di Leone, B. A., & Vogt, D. (2017). The role of postdeployment social factors in linking deployment experiences and current posttraumatic stress disorder symptomatology among male and female veterans. *Anxiety, stress, and coping*, *30*(1), 39-51. <http://ovidsp.ovid.com/ovidweb.cgi?T=JS&PAGE=reference&D=emed18&NEWS=N&AN=620875356>

Smith, N. A., Brady, J. M., Hammer, L. B., Carlson, K. F., & Mohr, C. D. (2020). Military sexual trauma among women Veterans: The buffering effect of coworker support. *Military Psychology*, *32*(6), 441-449. <https://doi.org/10.1080/08995605.2020.1806635>

Stefanovics, E. A., Potenza, M. N., Tsai, J., Nichter, B., & Pietrzak, R. H. (2023). Sex-specific risk and resilience correlates of suicidal ideation in U.S. military veterans [Behavior Disorders & Antisocial Behavior 3230

Military Psychology 3800]. *Journal of Affective Disorders*, *328*, 303-311. <https://doi.org/https://dx.doi.org/10.1016/j.jad.2023.02.025>

Street, A. E., Stafford, J. A., Mahan, C. M., & Hendricks, A. M. (2008). Sexual harassment and assault experienced by reservists during military service: prevalence and health correlates. *Journal of Rehabilitation Research and Development*, *45*(3), 409-419. <https://doi.org/https://doi.org/10.1682/JRRD.2007.06.0088>

Sumner, J. A., Lynch, K. E., Viernes, B., Beckham, J. C., Coronado, G., Dennis, P. A., ..., & Ebrahimi, R. (2021). Military sexual trauma and adverse mental and physical health and clinical comorbidity in women veterans. *Women's Health Issues*, *31*(6), 586-595. <https://doi.org/doi.org/10.1016/j.whi.2021.07.004>

Surís, A., Lind, L., Kashner, T. M., Borman, P. D., & Petty, F. (2004). Sexual assault in women veterans: an examination of PTSD risk, health care utilization, and cost of care. *Psychosom Med*, *66*(5), 749-756. <https://doi.org/10.1097/01.psy.0000138117.58559.7b>

Surís, A. M., Lind, L., Kashner, T. M., & Borman, P. D. (2007). Mental health, quality of life, and health functioning in women veterans: differential outcomes associated with military and civilian sexual assault. *JOURNAL OF INTERPERSONAL VIOLENCE*, *22*(2), 179-197. <https://doi.org/https://doi.org/10.1177/0886260506295347>

Vogt, D., Smith, B. N., King, L. A., King, D. W., Knight, J., & Vasterling, J. J. (2013). Deployment risk and resilience inventory-2 (DRRI-2): an updated tool for assessing psychosocial risk and resilience factors among service members and veterans. *Journal of Traumatic Stress*, *26*(6), 710–717. <https://doi.org/https://doi.org/10.1002/jts.21868>

Webermann, A., Relyea, M., Portnoy, G., Martino, S., Brandt, C., & Haskell, S. (2023). The role of unit and interpersonal support in military sexual trauma and posttraumatic stress disorder symptoms. *JOURNAL OF INTERPERSONAL VIOLENCE*. <https://doi.org/https://doi.org/10.1177/08862605231165764>

Wilson, L. C., Newins, A. R., Wilson, S. M., Elbogen, E. B., Dedert, E. A., Calhoun, P. S., Beckham, J. C., Workgroup, V. A. M.-A. M., & Kimbrel, N. A. (2020). Self- and other-directed violence as outcomes of deployment-based military sexual assault in Iraq/Afghanistan-era veteran men and women. *J Aggress Maltreat Trauma*, *29*(6), 714-724. <https://doi.org/10.1080/10926771.2020.1725213>

Wolfe, J., Brown, P. J., Furey, J., & Levin, K. B. (1993). Development of a wartime stressor scale for women. *Psychological Assessment*, *5*(3), 330-335. <https://doi.org/10.1037/1040-3590.5.3.330>

Wolfe, J., Sharkansky, E. J., Read, J. P., Dawson, R., Martin, J. A., & Ouimette, P. C. (1998). Sexual harassment and assault as predictors of PTSD symptomatology among U.S. female Persian Gulf War military personnel. *Journal of Interpersonal Violence 13*(1), 40-57. <https://doi.org/https://doi.org/10.1177/088626098013001003>

Yaeger, D., Himmelfarb, N., Cammack, A., & Mintz, J. (2006). DSM-IV diagnosed posttraumatic stress disorder in women veterans with and without military sexual trauma. *Journal of general internal medicine*, *21*(3), S65-S69. <https://doi.org/doi>.org/10.1111/j.1525-1497.2006.00377.x

Yalch, M. M., Hebenstreit, C. L., & Maguen, S. (2018). Influence of military sexual assault and other military stressors on substance use disorder and PTS symptomology in female military veterans. *Addict Behav*, *80*, 28-33. <https://doi.org/10.1016/j.addbeh.2017.12.026>

Zelkowitz, R. L., Sienkiewicz, M. E., Vogt, D. S., Smith, B. N., & Mitchell, K. S. (2022). Gender differences in direct and indirect associations of trauma types with disordered eating in a national U.S. veteran sample. *Psychol Trauma*. <https://doi.org/10.1037/tra0001353>

Zerach, G. (2023). The contribution of childhood adversity and potentially traumatic events during military service to PTSD and complex PTSD symptoms among Israeli women veterans. *European Psychiatry*, *66*(S1), S179–S179. <https://doi.org/doi:10.1192/j.eurpsy.2023.430>
